# Social support and internalizing psychopathology among sexual and gender minority individuals: A meta-analysis

**DOI:** 10.1016/j.cpr.2025.102686

**Published:** 2025-12-05

**Authors:** Benjamin A. Katz, Mertcan Mutlu, Atulya Arya Kharbanda, Palomi Kurade, Geva Shenkman

**Affiliations:** a School of Psychological Sciences, Haifa University, Israel; b Department of Psychology, Stony Brook University, United States of America; c Faculty of Behavioural and Social Sciences, University of Groningen, the Netherlands; d Department of Psychology, University of Wisconsin, Milwaukee, United States of America; e Yale School of Medicine, Yale University, United States of America; f Dina Recanati School of Medicine, Reichman University, Israel

**Keywords:** Sexual minority, Gender minority, Social support, Minority stress

## Abstract

Sexual and gender minority (SGM) individuals often suffer from a myriad of stressors within their social environments due to stigma and its outcomes (Meyer, 2003). Conversely, social support may impact SGM individuals’ psychological resilience. To quantify the impact of their interpersonal environments, the current preregistered meta-analysis included 253 studies (*N* = 111,188) that reported associations between social support (i.e., family, peer, partner, school, work) and internalizing psychopathology (i.e., depression, anxiety, nonsuicidal self-injury, suicidality) among SGM samples. Overall, a small, negative association was observed, *r* = − 0.26, with low levels of variance between studies. Larger effects were observed for depression than other measures of psychopathology, *r* = − 0.29, while smaller effects were observed for suicidality, *r* = − 0.17. Smaller effects were observed for samples with a larger share of bisexual individuals, and when a SGM-specific measure was used. Larger effects were observed for European studies performed in areas of greater structural stigma (but not for American studies). The negative association between social support and psychopathology was consistent across an array of demographic factors (e.g., age, gender, % white) and SGM identities (e.g., % gender minority). No evidence of publication bias was observed. These findings suggests that all forms of SGM social support are similarly associated with lower internalizing psychopathology to a small degree, most of all with regards to depression.

Sexual and gender minority (SGM) individuals are at a higher risk for adverse outcomes than cisgender, heterosexual individuals for a range of internalizing psychopathology outcomes, such as increased rates of depression, anxiety, non-suicidal self-injury (NSSI), suicidal ideation, and suicide attempts (Fergusson, Horwood, & Beautrais, 1999; Hatzenbuehler & Pachankis, 2016; Herek & Garnets, 2007; Hottes, Bogaert, Rhodes, Brennan, & Gesink, 2016; King et al., 2008; Russell & Fish, 2016; Wittlin, Kuper, & Olson, 2023). Minority stress theory posits that these disparities are not due to any inherent difference between SGM individuals and their heterosexual, cisgendered peers (Frost & Meyer, 2023; Meyer, 2003; Meyer, Pachankis, & Klein, 2021). Rather, health disparities arise in part from SGM individuals’ heightened exposure to social stressors related to their stigmatized identity.

Stigma impacts mental health through a variety of mechanisms that range from psychological (e.g., internalized homophobia; Cain et al., 2017) to structural (Hatzenbuehler, 2016); from SGM-specific (e.g., discrimination on the basis of SGM identity; Corrigan & Matthews, 2003) to those common across populations (e.g., emotion dysregulation; Burton, Wang, & Pachankis, 2018); from victimization at the hands of the majority population (Doan Van, Mereish, Woulfe, & Katz-Wise, 2019; Watson, Allen, Flores, Serpe, & Farrell, 2019) to victimization at the hands of fellow SGM individuals (Jackson, Mohr, Sarno, Kindahl, & Jones, 2020; Pachankis et al., 2020). SGM individuals must navigate a world in which their sexual orientation or gender identity is prone to be mischaracterized, discriminated against, or outright rejected (Frost & Meyer, 2023).

## 1. SGM social support and mental health

Humans benefit from social support and suffer when ostracized. Subjective experiences of social support are associated with a range of positive outcomes across the lifecycle (Krueger & Upchurch, 2022; Schwartz-Mette, Shankman, Dueweke, Borowski, & Rose, 2020; Shor et al., 2013). Subjective experiences of rejection, on the other hand, negatively impact well-being, even when disconnected from any material implications (Goodwin, Williams, & Carter-Sowell, 2010). Across the continuum of acceptance to rejection, humans are impacted by the social evaluation of others. Indeed, perceived experiences of loneliness play a larger role in internalizing psychopathology than objective metrics of how much time is spent in the company of others (Antonelli-Salgado et al., 2021; Katz, Karalis, Hawes, & Klein, 2024). Previous meta-analyses in general populations have reported moderate associations between social support and internalizing psychopathology (e.g., *r* = 0.35, *r* = 0.26, respectively; Gutiérrez-Sánchez, Orgeta, López-Martínez, & del-Pino-Casado, R., 2023; Rueger, Malecki, Pyun, Aycock, & Coyle, 2016). Yet, given the heightened rates of internalizing psychopathology among SGM populations (King et al., 2008) and their vulnerability to stigma (Meyer, 2003), it is pertinent to examine whether social support serves as a more potent protective factor for them compared to cisgender and heterosexual individuals.

Differences in social support and rejection serve as a key pathway to disparities between SGM and heterosexual, cisgendered individuals in internalizing psychopathology (e.g., Marshal et al., 2011). This is especially true within environments that expose them to greater levels of social stress (Feinstein, Wadsworth, Davila, & Goldfried, 2014; Palmer & Francis, 2024). As such, social support may have a particularly salubrious effect for reducing this disparity (Burton, Bonanno, & Hatzenbuehler, 2014; K. A. Clark et al., 2023; McConnell, Birkett, & Mustanski, 2016). Indeed, the positive associations between SGM individuals’ interpersonal environments and their mental health have been regularly studied and replicated (Krueger & Upchurch, 2022; Zimmerman, Darnell, Rhew, Lee, & Kaysen, 2015). Subjective support has been associated with lower levels of depression, anxiety, non-suicidal self-injury, and suicidality (Christoffersen, Møhl, DePanfilis, & Vammen, 2015; Rueger et al., 2016; Scardera et al., 2020). Social rejection, on the other hand, is associated with greater levels across all these forms of internalizing psychopathology (Cohen, Feinstein, Rodriguez-Seijas, Taylor, & Newman, 2016; Slavich, O’Donovan, Epel, & Kemeny, 2010).

Narrative reviews on this broad area of literature have established the association between SGM social support and SGM internalizing psychopathology (e.g., Hall, 2018; Hatzenbuehler, 2009; Pachankis, 2007; Peplau & Fingerhut, 2007; Russell & Fish, 2016; Valentine & Shipherd, 2018). However, they cannot precisely estimate the size of the effect. Furthermore, only a quantitative meta-analysis may assess the extent to which effects are generally uniform across the literature, or identify potential factors that may moderate the effect. Thus, a quantitative meta-analysis may help identify which sources of social support, and under which conditions, may yield the largest effects. This information is particularly relevant, as limited resources often dictate identifying and prioritizing interventions that focus on associations with the largest effects (Chaudoir et al., 2017).

## 2. Possible moderators of the association between SGM social support and internalizing psychopathology

Some possible moderators include the source of support being offered, the type of disorder, demographic characteristics of the SGM individual, or the social climate in which the support occurs.

### Source of support.

Social support is a multidimensional construct that comes from a variety of sources (e.g., partner, parents, siblings, peers; Zimet, Dahlem, Zimet, & Farley, 1988). A few studies have examined whether different sources of support hold different roles for SGM individuals (Clark et al., 2023; Kiekens & Mereish, 2022; Mustanski & Liu, 2013). However, such studies are an exception. Most either combine support across sources into a single, aggregate variable (e.g., Legate et al., 2012) or only focus on individual domains, such as peer support (e.g., Ybarra et al., 2015), family support (e.g., Ryan et al., 2010), or school support (e.g., Brandon-Friedman & Kim, 2016). Narrative reviews cannot quantitatively differentiate between the effect sizes yielded by these different sources and typically combine them into a single category to describe the importance of social support, in general (e.g., Lick et al., 2013; Russell & Fish, 2016). Indeed, the literature is divided with regards to the extent to which the source of social support would impact its association with psychopathology. Some studies, for example, indicate that family support would be larger than other sources, such as peer or school support (e.g., Hill et al., 2017; Kim et al., 2019; Marsland et al., 2022). Others indicate no difference or even the opposite (e.g., Bartoshuk, 2009; Frank, 2016). A meta-analysis can compare effects across studies to pinpoint which sources of support are associated with the largest effects, even when accounting for the variance of effect sizes that can be found within the literature.

### Disorder.

In both individual studies (e.g., Budge, Adelson, & Howard, 2013; Testa et al., 2017) and narrative reviews (e.g., Russell & Fish, 2016), social support has been linked to reduced levels of depression and anxiety symptoms. However, some disorders are more closely linked to social support than others. Interpersonal theories of depression (Hames, Hagan, & Joiner, 2013) and suicidality (Chu et al., 2017) suggest these aspects of internalizing psychopathology would be strongly linked to social support, or a lack thereof. Indeed, a meta-analysis within the general population indicated that social support is more negatively associated with depression, NSSI, and suicidality than with anxiety (Rueger et al., 2016; Schwartz-Mette et al., 2020). Similarly, among SGM populations, depression has often been found to show largest effect sizes (e.g., Puckett et al., 2019; Rivas-Koehl et al., 2022), but not always (e.g., Takeda et al., 2021). A meta-analysis that summarizes the literature on social support may directly compare which disorders are most strongly associated with social support for SGM individuals. Often, such quantitative analyses provide the basis for precision assessment in the face of multiple risk factors, including those pertaining to lower levels of support (see Feinstein et al., 2014).

### General Support vs SGM-Specific.

SGM individuals contain a range of identities with different standings in their interpersonal environments. For many, their SGM identity is of uncertain standing and therefore particularly salient (Chong et al., 2023). Most well-validated measures of social support assess holistic support within different spheres of an individual’s life (e.g., friends vs family; Zimet et al., 1988). Novel measures have been developed more recently to focus on experiences of support specifically in terms of individuals’ SGM identity (e.g., Gender Minority Stress and Resilience Measure; Testa, Habarth, Peta, Balsam, & Bockting, 2015). Both general and specific measures of support are associated with lower levels of internalizing psychopathology. However, it is not clear whether perceived SGM-specific support yields larger effects for mental health than would more general measures of support.

### Bisexuality/pansexuality.

Bisexual and pansexual individuals consistently report poorer mental health compared to lesbian women and gay men (e.g., Bostwick, Boyd, Hughes, & McCabe, 2010). This may be due to unique forms of biphobic stigma that are not experienced by their monosexual peers (Doan Van et al., 2019; Mereish, Katz-Wise, & Woulfe, 2017). Despite their greater share of the population, bisexuality and pansexuality tend to be less visible, both generally and within LGBTQ+ communities (Steinman, 2000). As such, social support may have an outsized impact for plurisexual individuals by providing a relief from some aspects of minority stress as well as validation of a less represented identity (e.g., Dyar & London, 2018), On the other hand, plurisexual individuals tend to view their sexual identity as less central to their overall identity than their lesbian or gay peers (Dyar, Feinstein, & London, 2015). If so, for those with plurisexual identities, social support may be less impactful (Benau, Jenkins, & Conner, 2017; Chaudoir & Fisher, 2010).

### Gender Identity.

Transgender and nonbinary individuals face an elevated risk of experiencing adverse mental health outcomes, both in comparison to cisgender heterosexual individuals (Guz et al., 2021), as well as cisgender sexual minority individuals (Price-Feeney, Green, & Dorison, 2020). These disparities are related to material stressors such as employment discrimination or loss of housing (Keuroghlian, Shtasel, & Bassuk, 2014; Lefevor, Boyd-Rogers, Sprague, & Janis, 2019). Other stressors occur via interpersonal harm, even without direct material implications. These stressors include exposure to more severe levels of identity invalidation, discrimination, verbal/cyber-bullying, and family rejection (Wittlin et al., 2023). It is thus possible that for transgender and nonbinary individuals, who are exposed to a greater range and intensity of social rejection, social support may yield larger protective effects Durwood et al., 2021; Valentine & Shipherd, 2018).

### Age.

SGM identity and social support play different roles in a SGM individual’s experience, as a function of stage of identity development and independence from their home families (Frost et al., 2020). As adolescents develop their identities in general, they are more sensitive to social feedback than children or young adults (Casey, Jones, & Hare, 2008; Milner, Krnjacki, & LaMontagne, 2016; Rodman, Powers, & Somerville, 2017). Furthermore, adolescent and young adult SGM individuals live under greater levels of parental control and are often more restricted in their self-expression or in their access to LGBTQ+ community activities (Hatzenbuehler & Pachankis, 2016). As such, the support that younger SGM individuals do receive may be particularly important.

### Multiple minoritization.

Minority stressors may be impacted by other minoritized identities related to an SGM individual’s race, ethnicity, or socioeconomic status (McConnell et al., 2018; Shangani, Gamarel, Ogunbajo, Cai, & Operario, 2020). SGM people of color often face the compounding effects of multiple stigmas, including racism from within the SGM community and heterosexism within their racial/ethnic community (Ching, Lee, Chen, So, & Williams, 2018; Everett, Steele, Matthews, & Hughes, 2019; Jackson et al., 2020; Meyer, Schwartz, & Frost, 2008). Individuals who belong to multiple minoritized groups, along with those of lower socioeconomic status, may be more materially impacted by discrimination related to housing, employment, healthcare, or community resources (Cyrus, 2017; Shangani et al., 2020; Ward, 2008). Furthermore, they often have smaller networks of support than SGM individuals who are white or wealthier (Meyer & Ouellette, 2009). Thus, social support may play an outsized role for those who contend with multiple forms of minoritization in comparison to those who are only minoritized on the basis of their SGM identity.

### Structural stigma.

Social support’s impact may also change as a function of structural context (Hatzenbuehler, 2016). Societal factors, such as regional laws, cultural norms, and institutional policies can play key roles in affirming and supporting SGM identity or suppressing it (Katz, 2025; Lattanner et al., 2021; Pachankis & Branstrom, 2019). While social support is likely to have positive effects across contexts, it may be more impactful when it occurs in contrast to higher levels of local structural stigma. Meta-analyses are uniquely equipped to quantify the extent to which statistically significant effect sizes may systematically differ across multiple contexts (Borenstein, 2009).

## 3. Aims and hypotheses

The current meta-analysis quantifies the associations between social support and internalizing psychopathology among SGM adolescents and adults. Social support consisted of perceived interpersonal support from participants’ immediate environments (i.e., family, peer, partner, school/work environment). Internalizing psychopathology consisted of adolescent and adult forms of distress- and mood-related psychopathology consistent with hierarchical taxonomies of psychopathology (i.e., depression, anxiety, NSSI, suicidality; see Ringwald et al., 2021). Studies were drawn from samples that were completely self-identified as SGM.

This study assesses whether associations systematically vary based on the source of social support (e.g., family) or the psychopathology measure (e.g., depression). It then explores whether these relationships are moderated by demographic aspects of the sample (i.e., bisexual/pansexual vs monosexual gender identity; gender minority vs sexual minority; age; race/ethnicity) or the levels of structural stigma in which the support occurs. This meta-analysis represents the first large-scale, quantitative summary of a broadly studied aspect of minority stress and resilience, and can provide insight into which kinds of social support may be most critical for whom in reducing the burden of SGM mental distress.

This meta-analysis was designed with a set of pre-registered hypotheses in mind. First, we expected a negative association between social support and internalizing psychopathology. Second, we expected the type of internalizing psychopathology (e.g., depression vs anxiety) to significantly moderate effect size. Third, we expected the source of support (e.g., peer vs family) to significantly moderate effect size. While we did expect to find other significant moderation effects, the literature is limited with regards to how these differences will occur. As such, post-hoc comparisons of effect sizes for hypothesized moderators, as well as other dimensional moderators (e.g., age, structural stigma, % gender minority), were examined in an exploratory manner.

## 4. Method

Hypotheses, as well as search, screening, and analysis procedures were preregistered with PROSPERO (ID: 332305) and OSF (https://osf.io/fjm8q).

### 4.1. Inclusion/exclusion criteria

SGM status was operationalized as either self-identification with any non-cisgender gender identity, a non-heterosexual sexual orientation, or participation in homosexual behavior in the past 12 months (e.g., men who have sex with men). Self-reported levels of masculinity or femininity that did not account for gender identity were not included.

Social support was operationalized as measures of subjective, interpersonal support (e.g., Multidimensional Scale of Perceived Social Support (MSPSS); Zimet et al., 1988) or interpersonal rejection (e.g., Anti-Bisexual Experiences Scale; Brewster & Moradi, 2010). Social behaviors (e.g., attending a LGBTQ+ center) were not included, as it is possible to behaviorally engage without feeling supported (e.g., Parmenter, Galliher, Wong, & Perez, 2021). Measures that included violence or material discrimination (e.g., Trans Discrimination Scale; Watson et al., 2019) and relationship styles (e.g., Rejection Sensitivity Questionnaire; Downey & Feldman, 1996) were excluded as they assess constructs that are different from interpersonal support. Similarly, the meta-analysis focused on interpersonal support, so relationships with nonspecific sources (e.g., “the gay community”, “people”) were excluded. Internalizing psychopathology was operationalized as validated measures of depression, anxiety, NSSI, or suicidality.

Owing to a lack of prospective and retrospective studies, only cross-sectional associations were included. Similarly, assessments of retrospective social support or lifetime psychopathology (vs current) were not included. Studies that assessed childhood levels of social support (e. g., Dorri, Stone, Salcido Jr, Russell, & Schnarrs, 2023) or psychopathology (e.g., Schnarrs et al., 2023) that did not include current assessments were not eligible for inclusion. Additionally, authors were contacted if potentially eligible data was collected but eligible bivariate associations were not reported. If authors did not furnish such associations, the studies were excluded from analysis. Finally, studies may have been performed in any language, but its report (e.g., journal article, dissertation) was required to be in English.

### 4.2. Search and screening process

**Fig. 1.**
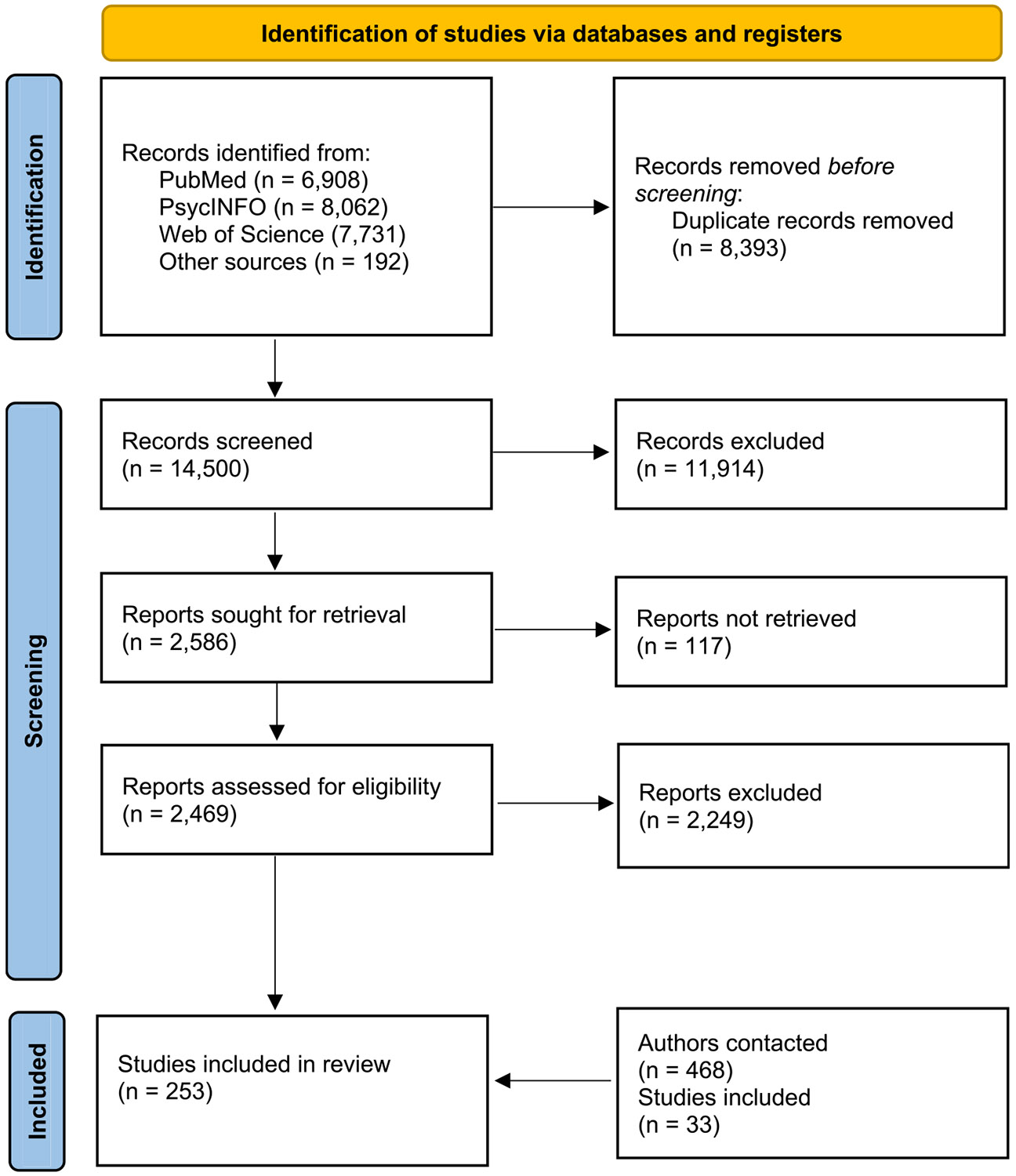
Summary of literature search for meta-analysis *Note*. Figure adapted from PRISMA 2020 guidelines (Page et al., 2021).

Studies were assembled using three strategies: First, PubMed and PsycInfo were searched for published articles and Web of Science for unpublished dissertations related to (a) social support (e.g., “families”, “Social Exclusion”) and (b) internalizing psychopathology (e.g., “depress*”, “NSSI”), among (c) sexual and/or gender minorities (e.g., “pansexual*”, “two-spirit”). Search terms as well as site-specific keywords (e.g., MeSH Terms: “Sexual and Gender Minorities”, “Social Support”) were assembled with the aid of two university librarians. Searches and screenings were performed from June 2022 to November 2023 and again from November 2024 to June 2025. (See Supplemental Material 1 for text of searches and number of references acquired from each database). We reviewed the reference sections of 17 narrative reviews and titles of articles published in 24 related journals (e.g., LGBT Health, Journal of Consulting and Clinical Psychology; see Supplemental Material 1 for full list) for possibly eligible articles. Finally, we posted requests for data and other relevant research on an academic listserv (Association for Behavioural and Cognitive Therapy’s Sexual and Gender Minority Special Interest Group) as well as on Twitter. This process yielded 22,893 references. References were then uploaded to Endnote X.9.3.2 and duplicates were eliminated, leaving 14,500 references approved for abstract screening (see Fig. 1).

References’ titles and abstracts were then reviewed through rayyan. Ai and were included if they mentioned data related to (a) social support and (b) internalizing psychopathology, (c) collected among SGM populations. References that did not indicate measurement of each of these constructs, or clearly indicated that no original data were collected (e.g., review article) were excluded, leaving 2586 references eligible for full-text review. 15 % of references were screened by more than one reviewer with high interrater reliability (kappa = 0.92) any disagreements were discussed in weekly meetings with the first author (BAK)

Full-text review followed the same criteria as the abstract review, but required an identification of specific measures that could yield effects eligible for the meta-analysis. Among the 2469 texts accessible for review, 210 were included in the meta-analysis outright. Authors of 468 studies were contacted up to three times for additional information related to effect sizes. Among these authors, 33 responded, leading to a final total of 243 studies in the meta-analysis. The majority of references (90 %) were screened by more than one reviewer with high interrater reliability (kappa = 0.98). Any disagreements were discussed in weekly meetings with the first author (BAK).

### 4.3. Data coding

Included studies were coded for effect size, sample size, demographic characteristics (i.e., age, gender identity, sexual orientation, race, ethnicity), source of social support, type of psychopathology, and metadata (i.e., publication status, year of publication, time and location of data collection). Effects were coded to represent a Pearson’s correlation between level of support and level of internalizing psychopathology. In cases where the social support measure was coded positively for rejection (as opposed to support), the text of the original effect was recorded and then reverse-scored prior to analysis. Such measures were coded accordingly for effects derived from measures of rejection vs those derived from measures of support. Similarly, odds ratios were recorded as text and then converted to Pearson’s correlations for analysis. In studies that offered multiple effects for the same association (e.g., family support and depression), the effects were averaged together prior to analysis. Multiple effects from the same study that represented different associations (e.g., family support and depression, peer support and depression) were coded separately. Measures of social support were also coded for whether they described SGM-specific support (e.g., Parental Attitudes of Gender Expansiveness Scale for Youth Perceived Parental Non-Affirmation; Hidalgo, Chen, Garofalo, & Forbes, 2017) or more general support (e.g., MSPSS; Zimet et al., 1988).

Structural stigma scores were calculated for American and European studies that reported the location of their participant pool. For studies performed in the United States, a composite score was calculated for the state’s average response to the General Social Survey (GSS; Smith, Marsden, Hout, & Kim, 2012) on six items related to opinions on sexual minority rights (e.g., COLHOMO: “Should a homosexual be allowed to teach in a college or university or not?”) or general social conservatism (e.g., POLVIEWS: a scale of political liberalism/conservatism). For studies performed in Europe, a score was calculated using three items from the European Social Survey (ESS; Jowell, Roberts, Fitzgerald, & Eva, 2007) measuring opinions on sexual minority rights (i.e., FREEHMS: “Gays and lesbians should be free to live life as they wish”) and general social conservatism (e.g., LRSCALE: a scale of political liberalism/conservatism). Calculation of averages followed standard sampling correction procedures for each survey (Jowell et al., 2007; Smith et al., 2012). Due to differences between the ESS and GSS scoring related to regional calculations (i.e., country vs state) and items, the structural variables were not standardized to be comparable to each other. Instead, each structural stigma’s analysis was performed separately.

### 4.4. Analysis plan

Effects were aggregated using a random effects meta-analysis, representing a weighted average as an inverse function of sample size (Borenstein, Hedges, Higgins, & Rothstein, 2021). Most studies offered more than one effect eligible for the meta-analysis, indicating the need for an analytic structure that may account for covariance among effects due to shared participants within studies. Often the covariance among effects were not reported, obviating the use of some multivariate approaches (see Fernández-Castilla et al., 2020; Riley et al., 2008). Rather, a multilevel meta-analytic structure was used, grouped by study (Cheung, 2014; Harrer, Cuijpers, Furukawa, & Ebert, 2021). Qualitative evaluations of effect sizes were preregistered to be: *r* < ∣0.1∣ = very small, *r* = ∣0.11–0.30∣ = small, *r* = ∣0.30–0.50∣ = moderate, *r* > ∣0.50∣ = large, with *p* values below 0.05 deemed significant.

Variance of meta-analytic effect sizes was quantified using standard metrics of homogeneity used in multilevel meta-analyses. Specifically, σ^2^_1_ was used to measure within-study variance and σ^2^_2_ measured between-study variance (equivalent to *τ*^2^ in a simple meta-analysis). Additionally, *I*_*Level 2*_^*2*^ and *I*_*Level 3*_^*2*^ were used to calculate the relative share of variance at the within-study and between-study levels, respectively. Normality of distribution of effects was assessed using a Shapiro-Wilk test (Shapiro & Wilk, 1965; see Razali & Wah, 2011). Evidence of non-normality (i.e., a significant Shapiro-Wilks test) led to a post-hoc visual evaluation of frequency distribution as well as measures of kurtosis and skew.

Moderators were assessed using meta-regressions within the multilevel meta-analytic structure (Harrer et al., 2021). Default estimators associated with the *meta* package (Schwarzer, 2007) were used to estimate between-study variance (i.e., DerSimonian-Laird estimator; DerSimionian & Laird, 1986) and confidence intervals for the summary effect (i.e., Jackson method; D. Jackson, 2013).

Publication bias was considered in multiple steps. First, a moderator analysis for main effects was performed using publication status as a moderator. Next, Egger’s test (Egger et al., 1997; Sterne et al., 2000) and a funnel plot were used to assess the likelihood that significant effects are more likely to be published in this area. To quantify the possible impact of effect size asymmetry, Duval and Tweedie’s (2000) “trim-and-fill” procedure was performed. Finally, we modelled *p* value distribution in order to examine whether effects with non-significant *p* values would be less likely to be included in the meta-analysis. This method of simulating selection models and comparing them to extant data has been lauded for its robustness and for its direct testing of hypothesized distribution biases (McShane, Böckenholt, & Hansen, 2016). In all cases, a significant test would indicate the potential of a biased distribution of effects.

### 4.5. Preregistered hypotheses

The following hypotheses were preregistered prior to data collection:

1. A negative association would be observed between social support and measures of internalizing psychopathology.
2. The type of internalizing psychopathology (e.g., depression vs anxiety) would significantly moderate effect size.
3. The source of support (e.g., peer vs family) would significantly moderate effect size.

Other potential moderators (e.g., age), were analyzed in an exploratory fashion.

## 5. Results

The descriptive information and references of the included studies may be found in Supplemental Material 2.

### 5.1. Description of studies

Two hundred and fifty-three studies were reviewed, including 270 samples, representing 111,118 participants (*M*_sample_ = 411.81, *SD*_sample_ = 631.96, rangesample = 27–6305). Participants were mostly adults (*M*_age_ = 28.26, *SD*_age_ = 9.41, range_age_ = 14.66–61.05) and represented a diverse range of demographics (Table 1). Around a third of participants were bisexual or pansexual (*M_plurisexual_* = 0.32).

Altogether, 689 effects were included. Slightly under two-thirds of included effects were published in peer-reviewed journals (429; 62.3 %). An additional 117 effects (17.0 %) were drawn from dissertations and masters’ theses available online, while 125 effects (18.1 %) were unpublished and attained through direct contact with the researcher. The smallest share of 18 effects (2.6 %) were derived from data shared with the authors and independently analyzed.

### 5.2. Multilevel meta-analysis

**Fig. 2.**
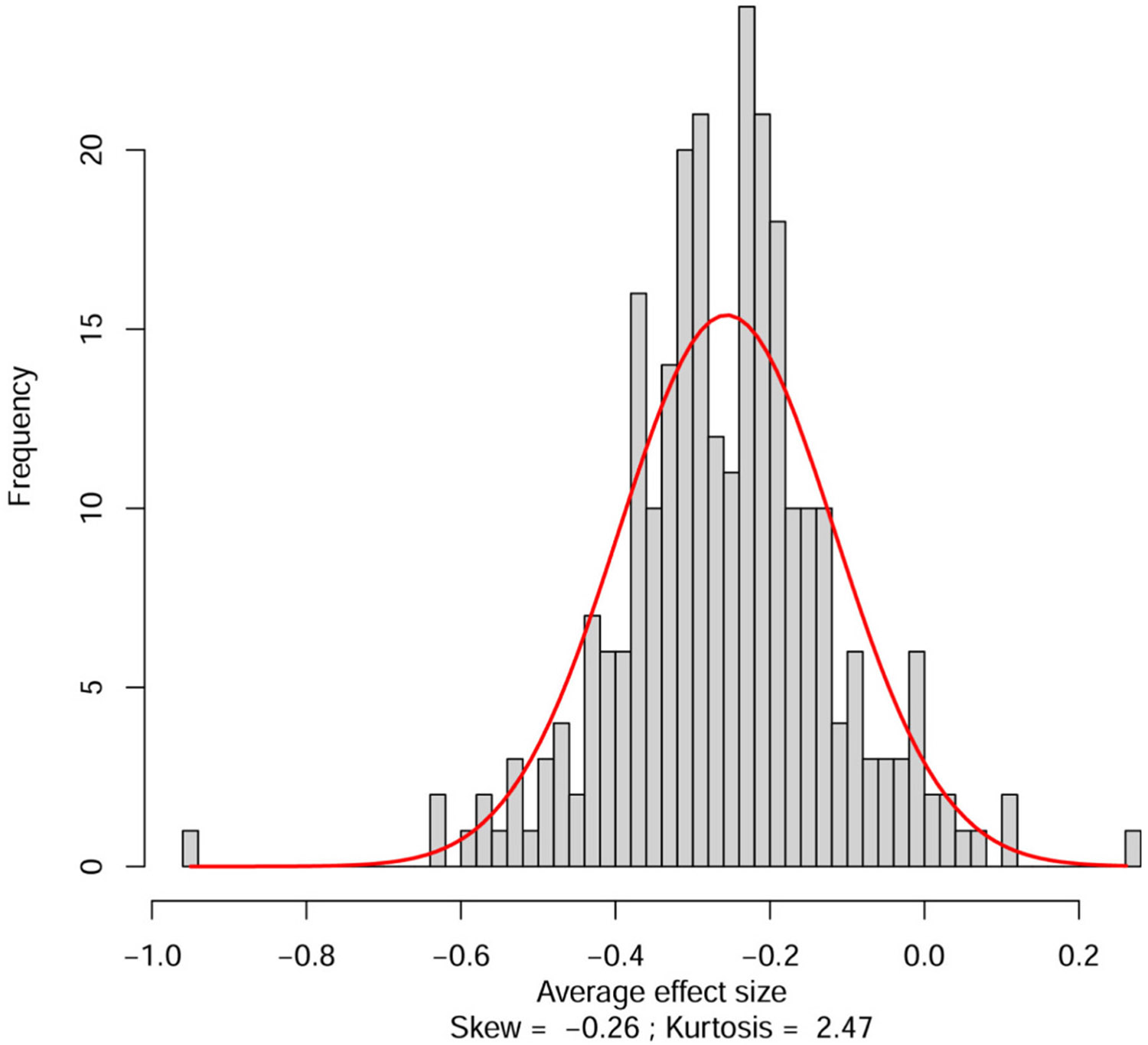
Histogram of effect sizes included in the meta-analysis *Note*. In order to reduce sample caused by repeated sampling, each study’s average effect size was included once.

Consistent with Hypothesis 1, a small, negative association was observed between levels of social support and internalizing psychopathology, *r* = −0.26, 95 % CI [−0.27; −0.24], 95 % PI [−0.55; 0.04]. A Shapiro-Wilk test revealed that the distribution of effects departed significantly from normality, *W* = 0.97, *p* < .001. A post-hoc review of the histogram of effects (Fig. 2) and metrics of distribution revealed that this departure from normality was the result of high kurtosis (= 2.47), as opposed to skew (= −0.26). This markedly low levels of heterogeneity was observed in the breakdown of variance as well. Most differences occurred among effects within the same study, σ^2^_1_ = 0.08, *I*^*LEVEL 2*^*_2_* = 64.62, as opposed to differences between studies, σ^2^_2_ = 0.015 *I*^*Level 3*^*_2_* = 34.67.

The moderation hypotheses were also supported (see Table 2). Consistent with Hypothesis 2, effects were moderated by the type of internalizing psychopathology, *F*(*df* = 4, 682) = 16.98, *p* < .001. Specifically, depression’s association with social support was significantly larger than the rest, *r* = −0.29 [−0.34; −0.24], and suicidality was smaller, *r* = −0.17 [−0.24; −0.11]. Hypothesis 3 was not supported, with source of social support not found to be a significant moderator overall, *F*(*df* = 3, 431) = 2.32, *p* = .075. However, among effects, family support, *r* = −0.26 [−0.28; −0.23] was found to be largest, followed by peer support, *r* = −0.22 [−0.28; −0.17], followed by partner support, *r* = −0.21 [−0.29; −0.14] and school/work environment support, *r* = −0.21 [−0.28; −0.14] Exploratory analyses did not indicate differences among sub-categories of social support either (Supplemental Material 3). For example, among peer support, no differences were found between whether the reference group was SGM peers or non-SGM peers, *F*(*df* = 1, 35) = 0.36, *p* = .551.

Exploratory moderator analyses also reflected the general homogeneity across studies (Table 3). Demographically, only the share of bisexuals within the sample was associated with effect size, where the association between social support and internalizing psychopathology grew more positive (i.e., smaller) as the share of bisexual participants in the sample increased, *B* = 0.07, *p* = .003. While age also significantly moderated effect sizes, it was to a trivially small degree, *B* = 0.00, *p* = .023.

Some research characteristics did moderate effect sizes. SGM-specific measures of social support yielded smaller effect sizes, *r* = 0.22, 95 % CI [−0.27; −0.17], than measures of more general support, *r* = −0.28, 95 % CI [−0.30; −0.26]. No difference was observed between effects derived from measures of support *r* = −0.25, 95 % CI [−0.29; −0.19] and measures derived from rejection, *r* = −*0.27*, 95 % CI 9–0.30; −0.25]. Year of publication also significantly moderated effect sizes, though, again, it was to a trivially small degree, *B* = 0.00, *p* = .037. Structural stigma moderated effect sizes for European studies, wherein greater structural stigma was associated with larger effect sizes, *B* = −0.12, *p* = .051 (Supplemental Material 4a). In American studies, however, this moderating effect was not observed, *B* = −0.06, *p* = .332 (Supplemental Material 4b). Thus, effects remained at similar levels across between-study differences in sample composition, study design, and the context in which the research was performed. However, effects were smaller with a larger share of bisexual participants and when using SGM-specific measures, and were larger in European studies in contexts of greater structural stigma.

### 5.3. Publication bias

**Fig. 3.**
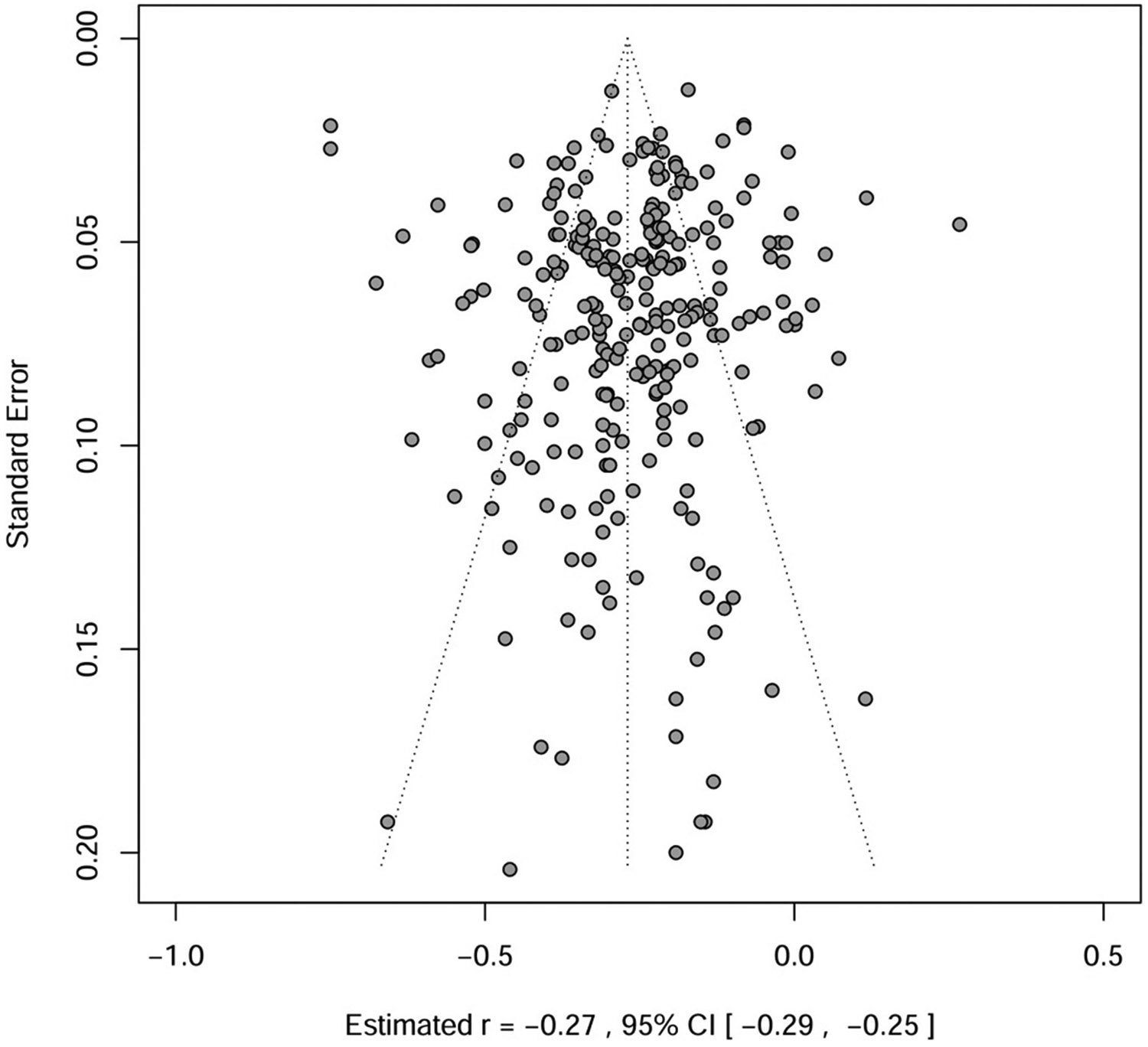
Funnel plot of effects using the trim-and-fill procedure *Note*. Empty circles indicate studies imputed in via the trim-and-fill procedure.

Effect sizes were not moderated by the source of effects (i.e., published article, dissertation, contacted for information), *F*(*df* 3, 685) = 1.69, *p* = .168, or by whether or not the effect was published, *F*(*df* 1, 687) = 3.39, *p* = .066. Similarly, Egger’s test indicated no asymmetry in effects as a function of sample size, *t*(268) = 0.64, *p* = .521, and the results of the trim-and-fill procedure did not impute any effect sizes for the sake of symmetry (Fig. 3). Finally, analysis of selection model parameters indicated that non-significant effects were not more likely to be included in the meta-analysis *F*(1) = 0.0004, *p* = .984. Taken together, results did not indicate a publication bias in the literature.

## 6. Discussion

The current meta-analysis quantitatively summarized the extensive literature on the associations between social support and internalizing psychopathology among SGM individuals. All sources of social support were negatively associated with all internalizing psychopathology measures to a small degree. Associations with depression and with family support were significantly larger than the forms of support and psychopathology. Associations with suicidality, on the other hand, were smaller. Effects were also smaller among samples with a larger share of bisexual participants and when SGM-specific measures of social support were used. Effects were larger within environments that contained greater structural stigma in Europe but not in the United States. Otherwise, no other factors moderated effect sizes to a significant or meaningful degree, including share of gender minority participants, demographic factors (i.e., gender, education level), and publication status did not moderate effect sizes.

One of the more remarkable findings from the current meta-analysis is the consistency of effect sizes across studies, with differences that were observed tending to be quite small. Social support played a significant role in SGM individuals’ levels of internalizing psychopathology across all demographic groups, all forms of internalizing psychopathology, and all sources of support. Indeed, effects across the literature varied less between studies than would be projected within a normal distribution. This relative consistency of effects is all the more notable within the minority stress literature, which often stresses the importance of intersectional factors (e.g., race/gender; Everett et al., 2019) and specific social environments (e.g., schools; Wright, Wachs, & Gámez-Guadix, 2022). Instead, the most parsimonious explanation for the current findings suggests that social support’s association with lower internalizing psychopathology impacts processes that occur beyond the particularities of any given demographic or social context.

Social support’s small associations across all forms of internalizing psychopathology are consistent with multiple theories that link social experiences with well-being, both in the general population (Cacioppo & Cacioppo, 2018; Holt-Lunstad, 2021), and for SGM individuals (Testa et al., 2017). Social support’s larger association with depression than with other psychopathology measures was consistent with theories that highlight interpersonal aspects of depression, such as loneliness, social isolation, and hopelessness (Haas et al., 2010; Hames et al., 2013). Conversely, higher levels of depression and social anhedonia may also lead individuals to be less sensitive to the support that they receive (Banica, Schell, Racine, & Weinberg, 2022; Gooding, Winston, Pflum, & Burgin, 2015; Katz et al., 2024; Mereish et al., 2017).

On the other hand, social support was less associated with suicidality than with other forms of internalizing psychopathology. This finding may be unexpected, considering both suicidality’s close association with depression (Hawton, Comabella, Haw, C. & Saunders, 2013) and feelings of interpersonal burdensomeness (Chu et al., 2017; Kaniuka et al., 2024; Testa et al., 2017). Furthermore, media representation of SGM narratives often depict strong, causal links between social rejection and suicidality (Marshall, 2010). Despite these initial impressions, meta-analyses from the general population also report smaller average effect sizes for the association between social support and suicidality than for that with depression (e.g., Darvishi et al., 2024; Gutiérrez-Sánchez et al., 2023). This may be due to a range of methodological and theoretical factors. Suicidal behaviors occur at a much lower rate than other forms of psychopathology and are often assessed with extremely brief inventories, often as short as only one item (e.g., Salentine et al., 2019). This may limit the amount of variance able to be interpreted. Additionally, correlations may be reduced as a result of a mismatch between the acute, episodic nature of suicidal crises versus the more chronic character of perceived social support (Jobs, 2023; Klonsky & May 2015). The current study is thus consistent with others from the general population in highlighting questions regarding the smaller association between suicidality and social support than among other forms of internalizing psychopathology.

Social support’s negative association with psychopathology was also observed across all sources of support. Support from family, peers, partners, school, and work all showing significant negative associations with psychopathology. This aligns with numerous research programs that have emphasized the importance of social support in SGM mental health, whether from family (Diamond et al., 2012; Mills-Koonce, Rehder, & McCurdy, 2018), peer group (Poteat, Godfrey, Brion-Meisels, & Calzo, 2020), or school environment (Brandon-Friedman & Kim, 2016). It also emphasizes the importance of research that examines the role of social support among multiple contexts, such as the role of family support in mitigating the distress in response to peer rejection (Clark et al., 2023).

The current study also underscores the importance of studying support and rejection experiences that are more unique to SGM populations. SGM individuals, for example, can accurately perceive the levels of safety in their environments due to homophobia – above and beyond nonspecific factors such as general conservatism or religiosity – and adjust their behaviors accordingly (Katz, in press; Lattanner et al., 2021). Based on the level of support in their environments, they may choose to conceal or reveal their identities to non-SGM family and peers (Bränström & Pachankis, 2021). This disclosure may often precipitate emotionally salient reactions from friends and family (Pachankis, Mahon, Jackson, Fetzner, & Bränström, 2020). Additionally, SGM communities and “families of choice” may provide their own unique suite of support and rejection experiences, through community and school support groups (Wright et al., 2022) or through competitiveness within SGM communities (Pachankis et al., 2020). At least in European contexts, these interpersonal reactions are particularly important within environments that maintain greater levels of structural stigma overall. For example, legal and policy reforms such as same-sex marriage laws have been shown to reduce distress and improve community safety among SGM communities of many locations and backgrounds (Hatzenbuehler et al., 2012). Each of these interpersonal experiences are associated with SGM internalizing psychopathology and continue to be potential avenues for fruitful research and intervention work.

All social environments play a role in shaping mechanisms at play in SGM risk and resilience. Social hypervigilance might be a learned adaptation for SGM individuals as a function of previous experiences with rejection (Hollinsaid, Pachankis, Bränström, & Hatzenbuehler, 2023; Pachankis & Jackson, 2023). However, it may also take the form of rejection sensitivity, a mechanism of minority stress (Pachankis et al., 2008). Social support in one context may play a key role in developing engendering resilience to rejection in others. Family support, for example, lowers levels of reactivity to peer-related stress among SGM individuals to a greater extent than their heterosexual peers (Burton et al., 2014; Clark et al., 2023; Kiekens & Mereish, 2022).

The current results do not necessarily indicate that SGM individuals as a group are uniquely sensitive (or insensitive) to social input overall, as compared to their heterosexual peers (cf. K.A. Clark et al., 2023). Effect sizes observed in the current meta-analysis (*r* = −0.17 to −0.26) are similar to those observed in meta-analyses that sample from the general population (*r* = 0.35, *r* = 0.26, respectively; Gutiérrez-Sánchez et al., 2023; Rueger et al., 2016). Indeed, SGM-specific measures were actually smaller than measures of general social support. Thus, the current findings do not indicate that associations between social support and internalizing psychopathology are larger for SGM individuals than for the general population. However, SGM individuals do face greater incidences of social adversity both within their communities (Pachankis et al., 2020) and from the general population (Kaufman et al., 2022), as well as greater levels of internalizing psychopathology (Marshal et al., 2011). Taken together, the current state of the literature indicates that the association of social support and well-being is similar for SGM individuals and for the general population. However, social support may still play an outsized impact on the lives of SGM individuals by virtue of their social environments being more perilous, possibly impacting their well-being accordingly.

The current findings align with interventions that aim to foster more supportive social contexts within families, peer networks, schools, and workplaces (e.g., Diamond et al., 2012; Green, Willging, Ramos, Shattuck, & Gunderson, 2018) – though the cross-sectional nature of the effects summarized cannot directly validate their mechanisms. Programs that help SGM individuals enhance their perceived or actual support (e.g., assertiveness training; Pachankis et al., 2022), or that leverage support in one setting (e.g., SGM peer groups) to buffer rejection in another (e.g., family; Zimmerman et al., 2015), remain theoretically grounded in the present evidence. Indeed, the current findings are consistent with evidence that enhancing SGM youths’ supportive relationships is associated with lower levels of depression and suicidality (Diamond et al., 2012; Diamond et al., 2016; Diamond & Shpigel, 2014).

The cross-sectional nature of the findings means that they align with the reverse argument as well, that internalizing symptoms may lead to fewer experiences of subjective support. Internalizing symptoms are associated with social dysfunction (Steare et al., 2025), social isolation (Wolters et al., 2023), and social exclusion (Reinhard, Dewald-Kaufmann, Wüstenberg, et al., 2020) – all of which would lead to fewer experiences of social support. Furthermore, even in cases of similar socializing behaviors, individuals higher in internalizing symptoms are less sensitive to social support (Katz et al., 2024) and more sensitive to social rejection (Gardner, Zimmer-Gembeck, & Modecki, 2020). Taken together, the current meta-analysis supports work that emphasizes the association between social affiliation in well-being. Future longitudinal work is required to further parse out the relationship between them.

Finally, it is notable that the share of bisexual participants moderated effect sizes so that a greater share of bisexuals predicted smaller associations between social support and psychopathology. This is consistent with findings that country-level structural stigma related to homophobia is impactful for bisexual individuals than for gay or lesbian individuals (Katz, in press; Lattanner et al., 2021). This may be owing to the fact that bisexual individuals face a wider range of discrimination experiences among both their heterosexual and gay and lesbian peers, in the form of both homophobia and biphobia (Doan Van et al., 2019; Mereish et al., 2017). These additional concerns are associated with lower levels of identity disclosure, lower levels of connection to SGM communities, and lower relationship satisfaction, and in turn, with greater internalizing psychopathology (Chan, Operario, & Mak, 2020; Dorrell, Benjamin, Dyar, Davila, & Feinstein, 2024; Dyar et al., 2021; Feinstein, Hurtado, Dyar, & Davila, 2023). Because bisexual individuals face a wider range of potential rejection experiences, it is possible that they require a wider range of support to lower their levels of internalizing psychopathology as well.

The current meta-analysis indicates that SGM individuals with multiple-minoritized identities (e.g., racial/ethnic minorities) do not show unique associations between social support and internalizing psychopathology. However, just as SGM individuals in general are impacted by social experiences that are unique from cisgender-heterosexual peers, multiple-minoritized SGM individuals are impacted by social experiences that are unique from many of their other SGM peers in ways that are relevant to their well-being. Many racial and ethnic SGM individuals report experiences of heterosexist bigotry and racism within queer spaces (Jackson et al., 2020; Soler, Caldwell, Córdova, Harper, & Bauermeister, 2018). They may also experience barriers to care related to cultural factors (e.g., Latino *machismo*) or language difficulties (Mayo et al., 2025). Similarly, gender minorities report their own unique difficulties in accessing affirmative, knowledgeable therapies (Mizock & Lundquist, 2016). Interventions focused on supporting SGM individuals with additional minoritized statuses should expect that the association between internalizing psychopathology and social support would be the same, and consider that the causal direction of this association remains to be studied. However, such interventions may still find it important to identify unique social stressors that arise from clients’ intersectional identities and work with them accordingly.

## 7. Limitations

Certain design characteristics should be considered when evaluating the current findings’ generalizability and when considering future directions for research. First, all effects were derived from between-subject, cross-sectional associations, while theories of social support and well-being generally focus on within-subject, longitudinal trajectories (Fisher, Medaglia, & Jeronimus, 2018; Katz-Wise et al., 2017; Toomey, 2021). The cross-sectional design may underestimate the impact of continual social support. The current study’s preregistration used more conservative cutoffs of effect sizes, with correlations between ∣0.10∣ and ∣0.30∣, such as the current study’s findings, being defined as “small.” Such effects fall well within the minority stress literature, that often includes similarly sized effects, such as the associations between SGM psychopathology with identity disclosure (i.e., “coming out of the closet”; Pachankis et al., 2020), religious observance (Lefevor, Davis, Paiz, & Smack, 2021), and internalized homonegativity (Badenes-Ribera et al., 2018). Indeed, it is similar in size to the association between personality and internalizing psychopathology within the general population as well (Kotov et al., 2010). However, this magnitude only accounts for the effects at a single evaluation. Social support, on the other hand, occurs regularly over time, across multiple contexts, impacting internalizing symptoms both cross-sectionally and prospectively (Dyar et al., 2020). Iterative experiences such as social support may show compounding effects, impacting mental health to a greater degree when consistently applied across larger timeframes (Funder & Ozer, 2019; Götz et al., 2022; Paulus & Thompson, 2019). On the other hand, effects may be over-estimated as well. Current levels of perceived social support and psychopathology may not necessarily remain stable over time. A brief period of social support, for example, may not have as large of an impact over time. Future research using longitudinal designs may more directly test minority stress theories in a developmental context (Dyar et al., 2020; McConnell et al., 2016).

Additionally, support and rejection were operationalized as emotionally salient interpersonal interactions. Doing so isolated the emotional aspect of social relationships but leaves open questions related to material and physical considerations that may arise within social relationships such as employment security at work (Bauermeister et al., 2014), and exposure to violence from intimate partners (Williams & Gutierrez, 2022) and strangers (Bränström, Fellman, & Pachankis, 2023). This is particularly true for gender minority individuals (Keuroghlian et al., 2014; Lefevor et al., 2019; Martin-Storey et al., 2018). Furthermore, connectedness to general SGM or religious communities may be additional sources of resilience, above and beyond interpersonal interactions (Kubicek et al., 2009; Meyer, 2015; Zimmerman et al., 2015). Meta-analyses of these literatures (e.g., Lefevor et al., 2021) will be of great use to identify the multifaceted ways in which SGM individuals experience their social environments, and how that may relate to their mental health.

The correlational studies included in the meta-analysis assumed linear associations and moderator analysis assumed consistent moderation across all levels of social support. However, certain time periods and levels of support (e.g., advocacy soon after disclosure; Camacho, Reinka, & Quinn, 2020; Feinstein et al., 2023) may be times in which SGM individuals’ internalizing symptoms are especially sensitive to their social environments. This is all the more relevant as recent cohorts of SGM individuals are becoming self-aware and disclosing their identity at younger ages than previous ones did (Bishop, Fish, Hammack, & Russell, 2020), during times when they are more embedded within their social structures. Research that focuses on SGM individuals at critical developmental periods or during transition periods where SGM individuals are unsure of their social standing will be particularly useful for answering such questions (Pachankis & Jackson, 2023).

Finally, studies could only be included insofar as their effects were accessible to project staff. Unsearchable theses and dissertations and manuscripts not published in English were not able to be included in the meta-analysis. Similarly, most authors contacted for further information regarding their studies’ effects were not responsive to overtures. Ideally, the current meta-analysis would include the effects derived from these studies as well.

## 8. Conclusion

Taken together, the current findings reveal a small negative association between social support and internalizing psychopathology for SGM individuals. While some significant moderators were observed – such as greater effect sizes for depression and smaller effect sizes for bisexuality and SGM measures – the upshot of this meta-analysis is the consistency of effects. Social support was negatively associated with internalizing psychopathology across all SGM identities, all sources of support, and all structural contexts. SGM individuals participate in a wide variety of interpersonal experiences, both generally and reflecting of their SGM identity. Interventions that highlight both the importance and variety of these experiences, such as in the SGM individual’s family or the school, may find support for their approaches here as well.

## Supplementary Material

1

## Figures and Tables

**Table 1:** Demographic Statistics of Samples.

Gender	Share of sample	Race/Ethnicity	Share of sample
Male	0.38	White	0.44
Female	0.32	Black/African American	0.11
Non-binary	0.06	Latinx	0.08
Transgender	0.18	Asian/Pacific Islander	0.14
Male	0.07	Middle Eastern/North African (MENA)	0.01
Female	0.08	Indigenous	0.01
Unspecified	0.03	Mixed	0.04
Other	0.05	Other	0.03

*Note*. Most articles did not specify the shares of participants coded as “male” and “female” who were cisgender or transgender. When participants were identified as transgender, they were coded separately for analysis, as represented in this table.

**Table 2:** Preregistered Meta-Analyses of Social Support and Psychopathology Among SGM Samples.

Category	*#* studies	*k* effects	*N*	*r*	95 % CI
Hypothesis 1: Main Effect					
Main Effect	253	689	111,188	−0.26	[−0.27; −0.24]
Hypothesis 2: Mental Health					
**Depression**	**204**	**369**	**76,569**	**−0.29**	**[−0.34; −0.24]**
Anxiety	88	167	29,734	−0.22	[−0.25; −0.20]
Mixed	21	36	6219	−0.21	[−0.30; −0.13]
Depression/Anxiety					
**Suicidality**	**42**	**99**	**22,077**	**−0.17**	**[−0.24; −0.11]**
NSSI	16	16	3929	−0.18	[−0.29; −0.08]
Hypothesis 3: Source of Social Support					
Family	104	183	39,122	−0.26	[−0.28; −0.23]
Peers	73	142	26,501	−0.22	[−0.28; −0.17]
Partner	35	63	12,747	−0.21	[−0.29; −0.14]
School/Work	22	47	11,307	−0.21	[−0.28; −0.14]

*Note*. *k* = number of effects; *N* = sample size; *r* = correlation between social support and psychopathology. Bolded effects indicate significant difference from other subgroups.

**Table 3:** Exploratory dimensional moderators.

Moderator	*#* studies	*k* effects	*N*	*B*	*p*
**Age**	**219**	**581**	**96,478**	**0.00**	**0.023**
White	239	651	105,076	0.02	0.312
**Bisexual**	**253**	**689**	**111,188**	**0.07**	**0.003**
Male	253	689	111,188	0.02	0.482
Female	253	689	111,188	−0.02	0.286
Non-Binary	253	689	111,188	0.06	0.325
Gender Minority	253	689	111,188	0.03	0.182
**Publication Year**	**253**	**689**	**111,188**	**0.00**	**0.037**
Structural Stigma					
**ESS**	**10**	**39**	**7093**	**−0.12**	**0.051**
GSS	74	183	30,016	−0.06	0.332

## Data Availability

The included studies are reported in S2. The data and analysis syntax are linked in the study’s OSF page.

## Appendix A. Supplementary data

Supplementary data to this article can be found online at https://doi.org/10.1016/j.cpr.2025.102686.
