# Supplementary material for: Social support and internalizing psychopathology among sexual and gender minority individuals: A meta-analysis
Source: Clin Psychol Rev. Author manuscript; Available in PMC 2026 Jun 28. (PMC13310347; doi:10.1016/j.cpr.2025.102686)
Supplement: 1 [file NIHMS2187466-supplement-1.pdf]

**Supplemental Material 1. Documentation Related to Search Process**

**S1a. Preregistered Search Protocol**

| | **Concept: Sexual/Gender Minorities** | **Concept: Social Support/Rejection** | **Concept: Internalizing Disorders/Well-being** |
|---|---|---|---|
| MeSH Terms (included in free search as well) | Sexual and Gender Minorities, Transgender Persons, Homosexuality, Bisexuality, "Homosexuality, Female", "Homosexuality, Male" | "Social Support", "Psychosocial Support Systems", Homophobia, "Family Relations", "Adult Children", Friends, "Family Conflict", "Intergenerational Relations", "Interprofessional Relations", "Social Integration", "Social Cohesion", "Social Determinants of Health", Bullying | Depression, "Depressive Disorder", Anxiety, "Anxiety Disorders", Suicide, "Suicidal Ideation", "Self-Injurious Behavior" |
| Title/Abstract search<br><br>PubMed: [Title]<br><br>PsycInfo and Web of Science: | gay, lesbian, homosexual*, bisexual*, pansexual*, sexuality, "sexual minority", "sexual minorities", "sexual orientation", queer*, "non-heterosexual", MSM , WSW, MSMW, WSWM, "men who have sex with men", "women who have sex with women", "men-loving men", "men loving men", "women-loving women", "women loving women", "same-gender loving", bicurious, lesbigay, transgender, "gender minority", "gender minorities", "gender diverse", | family, families, mother, father, maternal, paternal, sibling, brother, sister, son, daughter, child*, partner*, spouse*, boyfriend*, girlfriend*, husband*, wife, wives, peer, peers, friend*, companion*, colleagues, school, work*, bully*, homophob*, transphobe*, "anti-homosexual", "anti-gay", "anti-transgender", neglect, abuse, rejection, "psychosocial support", "support system", "social care" , | depress*, dysthym*, melancholia, anxi*, "internalizing disorder", "internalizing disorders", "mental disorder", "mental disorders", "mental health", "chronic mental illness", "affective disorders", melancholia*, "self-injury", "self injury", "self-injurious", "self injurious" "self-harm", "self harm", NSSI, suicid* |

| | **Concept: Sexual/Gender Minorities** | **Concept: Social Support/Rejection** | **Concept: Internalizing Disorders/Well-being** |
|---|---|---|---|
| Title and abstract (TI/AB) | “gender identity”, “gender identities”, “two spirit”, “two-spirit” , “same sex couple”, “same-sex couple”, “same sex relationship”, “same-sex relationship”, “same sex relations”, “same-sex relations”, LGB, LGBQ, LGBT*, GLB, GLBT*, asexual*, demisexual* | “social determinant”, “social determinants” | |
| Psychological Index Terms (DE; only used in PsycInfo) | LGBTQ, Bisexuality, Homosexuality, Transgender | Social Support, Social Acceptance, Belonging, Social Connectedness, Social Approval, Social Isolation, Social Interaction, Encouragement, Interpersonal Interaction, Interpersonal Relationships, Teasing, Victimization, Social Discrimination, Social Integration, Social Exclusion, Social Stress, Social Inclusion, Homosexuality (Attitudes Toward), Transgender (Attitudes Toward), Family Conflict, Intergenerational Relations, Parent Child Relations, Sibling Relations, Social Support, Threat, Relational Aggression, Harassment | Major Depression, Dysthymic Disorder, Endogenous Depression, Late Life Depression, Reactive Depression, Recurrent Depression, Treatment Resistant Depression, Depression (Emotion), Anxiety, Anxiety Sensitivity, Social Anxiety, Speech Anxiety, Anxiety Disorders, Generalized Anxiety Disorder, Obsessive Compulsive Disorder, Panic Attack, Panic Disorder, Phobias, Social Phobia, Suicidal Behavior, Attempted Suicide, Suicide, Suicidal Ideation, Suicidality, Suicide, Self-Injurious Behavior, Self-Inflicted Wounds, Self-Mutilation |

**Filters:**

- English

**Database: PubMed**

("Sexual and Gender Minorities" [MeSH Terms] OR "Transgender Persons" [MeSH Terms] OR Homosexuality [MeSH Terms] OR Bisexuality [MeSH Terms] OR "Homosexuality, Female" [MeSH Terms] OR "Homosexuality, Male" [MeSH Terms] OR gay [Title/Abstract] OR lesbian [Title/Abstract] OR homosexual* [Title/Abstract] OR bisexual* [Title/Abstract] OR pansexual* [Title/Abstract] OR sexuality [Title/Abstract] OR "sexual minority" [Title/Abstract] OR "sexual minorities" [Title/Abstract] OR "sexual orientation" [Title/Abstract] OR queer* [Title/Abstract] OR "non-heterosexual" [Title/Abstract] OR MSM [Title/Abstract] OR WSW [Title/Abstract] OR MSMW [Title/Abstract] OR WSWM [Title/Abstract] OR "men who have sex with men" [Title/Abstract] OR "women who have sex with women" [Title/Abstract] OR "men-loving men" [Title/Abstract] OR "women-loving women" [Title/Abstract] OR "same-gender loving" [Title/Abstract] OR bicurious [Title/Abstract] OR lesbigay [Title/Abstract] OR transgender [Title/Abstract] OR "gender minority" [Title/Abstract] OR "gender minorities" [Title/Abstract] OR "gender diverse" [Title/Abstract] OR "gender identity" [Title/Abstract] OR "gender identities" [Title/Abstract] OR "two spirit" [Title/Abstract] OR "two-spirit" [Title/Abstract] OR "same sex couple" [Title/Abstract] OR "same-sex couple" [Title/Abstract] OR "same sex relationship" [Title/Abstract] OR "same-sex relationship" [Title/Abstract] OR "same sex relations" [Title/Abstract] OR "same-sex relations" [Title/Abstract] OR LGB [Title/Abstract] OR LGBQ [Title/Abstract] OR LGBT* [Title/Abstract] OR GLB [Title/Abstract] OR GLBT* [Title/Abstract] OR asexual* [Title/Abstract] OR demisexual* [Title/Abstract])

AND ("Social Support" [MeSH Terms] OR "Psychosocial Support Systems" [MeSH Terms] OR Homophobia [MeSH Terms] OR "Family Relations" [MeSH Terms] OR "Adult Children" [MeSH Terms] OR Friends [MeSH Terms] OR "Family Conflict" [MeSH Terms] OR "Intergenerational Relations" [MeSH Terms] OR "Interprofessional Relations" [MeSH Terms] OR "Social Integration" [MeSH Terms] OR "Social Cohesion" [MeSH Terms] OR "Social Determinants of Health" [MeSH Terms] OR Bullying [MeSH Terms] OR family [Title/Abstract] OR mother [Title/Abstract] OR father [Title/Abstract] OR maternal [Title/Abstract] OR paternal [Title/Abstract] OR sibling [Title/Abstract] OR brother [Title/Abstract] OR sister [Title/Abstract] OR son [Title/Abstract] OR daughter [Title/Abstract] OR child* [Title/Abstract] OR partner [Title/Abstract] OR spouse [Title/Abstract] OR boyfriend [Title/Abstract] OR girlfriend

[Title/Abstract] OR husband [Title/Abstract] OR wife [Title/Abstract] OR peers [Title/Abstract] OR friend* [Title/Abstract] OR companion* [Title/Abstract] OR colleagues [Title/Abstract] OR school [Title/Abstract] OR work* [Title/Abstract] OR bully* [Title/Abstract] OR homophob* [Title/Abstract] OR transphobe* [Title/Abstract] OR "anti-homosexual" [Title/Abstract] OR "anti-gay" [Title/Abstract] OR "anti-transgender" [Title/Abstract] OR neglect [Title/Abstract] OR abuse [Title/Abstract] OR rejection [Title/Abstract] OR "psychosocial support" [Title/Abstract] OR "support system" [Title/Abstract] OR "social care" OR "Social Support" [Title/Abstract] OR "Intergenerational Relations" [Title/Abstract] OR "Interprofessional Relations" [Title/Abstract] OR "Social Integration" [Title/Abstract] OR "Social Cohesion" [Title/Abstract] OR “social determinant” [Title/Abstract] OR “social determinants” [Title/Abstract])

AND (Depression [MeSH Terms] OR “Depressive Disorder” [MeSH Terms] OR Anxiety [MeSH Terms] OR “Anxiety Disorders” [MeSH Terms] OR Suicide [MeSH Terms] OR “Suicidal Ideation” [MeSH Terms] OR “Self-Injurious Behavior” [MeSH Terms] OR depress* [Title/Abstract] OR dysthym* [Title/Abstract] OR melancholia [Title/Abstract] OR anxi* [Title/Abstract] OR "internalizing disorder" [Title/Abstract] OR "internalizing disorders” [Title/Abstract] OR "mental disorder" [Title/Abstract] OR "mental disorders" [Title/Abstract] OR "mental health" [Title/Abstract] OR "chronic mental illness" [Title/Abstract] OR "affective disorders" [Title/Abstract] OR melancholia* [Title/Abstract] OR "self-injury" [Title/Abstract] OR “self injury” [Title/Abstract] OR "self-injurious" [Title/Abstract] OR “self injurious” [Title/Abstract] OR “self-harm” [Title/Abstract] OR “self harm” [Title/Abstract] OR NSSI [Title/Abstract] OR suicid* [Title/Abstract])

AND (english[Filter])

**Results:** 6,908

**Database: PsycINFO**

**<u>Final Search:</u>**

(MA("Sexual and Gender Minorities"  OR "Transgender Persons"  OR Homosexuality  OR Bisexuality  OR "Homosexuality, Female"  OR "Homosexuality, Male")
OR AB(gay  OR lesbian  OR homosexual*  OR bisexual*  OR pansexual*  OR sexuality  OR "sexual minority"  OR "sexual minorities"  OR "sexual orientation"  OR queer*  OR "non-heterosexual"  OR MSM  OR WSW  OR MSMW  OR WSWM  OR "men who have sex with men"  OR "women who have sex with women"  OR "men-loving men"  OR "women-loving women"  OR "same-gender loving"  OR bicurious  OR lesbigay  OR transgender  OR "gender minority"  OR "gender minorities"  OR "gender diverse"  OR "gender identity"  OR "gender identities"  OR "two spirit"  OR "two-spirit"  OR "same sex couple"  OR "same-sex couple"  OR "same sex relationship"  OR "same-sex relationship"  OR "same sex relations"  OR "same-sex relations"  OR LGB  OR LGBQ  OR LGBT*  OR GLB  OR GLBT*  OR asexual*  OR demisexual* )

OR TI(gay  OR lesbian  OR homosexual*  OR bisexual*  OR pansexual*  OR sexuality  OR "sexual minority"  OR "sexual minorities"  OR "sexual orientation"  OR queer*  OR "non-heterosexual"  OR MSM  OR WSW  OR MSMW  OR WSWM  OR "men who have sex with men"  OR "women who have sex with women"  OR "men-loving men"  OR "women-loving women"  OR "same-gender loving"  OR bicurious  OR lesbigay  OR transgender  OR "gender minority"  OR "gender minorities"  OR "gender diverse"  OR "gender identity"  OR "gender identities"  OR "two spirit"  OR "two-spirit"  OR "same sex couple"  OR "same-sex couple"  OR "same sex relationship"  OR "same-sex relationship"  OR "same sex relations"  OR "same-sex relations"  OR LGB  OR LGBQ  OR LGBT*  OR GLB  OR GLBT*  OR asexual*  OR demisexual*)

OR DE(LGBTQ OR Bisexuality OR Homosexuality OR Transgender) )

AND (MA("Social Support" OR "Psychosocial Support Systems" OR Homophobia OR "Family Relations" OR "Adult Children" OR Friends OR "Family Conflict" OR "Intergenerational Relations" OR "Interprofessional Relations" OR "Social Integration" OR "Social Cohesion" OR Bullying)

OR AB(family OR mother OR father OR maternal OR paternal OR sibling OR brother OR sister OR son OR daughter OR child* OR partner OR spouse OR boyfriend OR girlfriend OR husband OR wife OR peers OR friend* OR companion* OR colleagues OR school OR work* OR bully* OR homophob* OR transphobe* OR "anti-homosexual" OR "anti-gay" OR

"anti-transgender" OR neglect OR abuse OR rejection OR "psychosocial support" OR "support system" OR "social care" OR "Social Support" OR "Intergenerational Relations" OR "Interprofessional Relations" OR "Social Integration" OR "Social Cohesion" OR “social determinant” OR “social determinants”)

OR TI(family OR mother OR father OR maternal OR paternal OR sibling OR brother OR sister OR son OR daughter OR child* OR partner OR spouse OR boyfriend OR girlfriend OR husband OR wife OR peers OR friend* OR companion* OR colleagues OR school OR work* OR bully* OR homophob* OR transphobe* OR "anti-homosexual" OR "anti-gay" OR "anti-transgender" OR neglect OR abuse OR rejection OR "psychosocial support" OR "support system" OR "social care" OR "Social Support" OR "Intergenerational Relations" OR "Interprofessional Relations" OR "Social Integration" OR "Social Cohesion" OR “social determinant” OR “social determinants”)

OR DE (“Social Support” OR “Social Acceptance” OR Belonging OR “Social Connectedness” OR “Social Approval” OR “Social Isolation” OR “Social Interaction” OR Encouragement OR “Interpersonal Interaction” OR “Interpersonal Relationships” OR Teasing OR Victimization OR “Social Discrimination” OR “Social Integration” OR “Social Exclusion” OR “Social Stress” OR “Social Inclusion” OR “Homosexuality (Attitudes Toward)” OR “Transgender (Attitudes Toward)” OR “Family Conflict” OR “Intergenerational Relations” OR “Parent Child Relations” OR “Sibling Relations” OR “Social Support” OR Threat OR “Relational Aggression” OR Harassment))

AND (MA(Depression OR "Depressive Disorder" OR Anxiety OR "Anxiety Disorders" OR Suicide OR "Suicidal Ideation" OR "Self-Injurious Behavior")

OR AB(depress* OR dysthym* OR melancholia OR anxi* OR "internalizing disorder" OR "internalizing disorders" OR "mental disorder" OR "mental disorders" OR "mental health" OR "chronic mental illness" OR "affective disorders" OR melancholia* OR "self-injury" OR "self injury" OR "self-injurious" OR "self injurious" OR "self-harm" OR "self harm" OR NSSI OR suicid*)

OR TI(depress* OR dysthym* OR melancholia OR anxi* OR "internalizing disorder" OR "internalizing disorders" OR "mental disorder" OR "mental disorders" OR "mental health" OR "chronic mental illness" OR "affective disorders" OR melancholia* OR "self-injury" OR "self injury" OR "self-injurious" OR "self injurious" OR "self-harm" OR "self harm" OR NSSI OR suicid*)

OR DE(“Major Depression” OR “Dysthymic Disorder” OR “Endogenous Depression” OR “Late Life Depression” OR “Reactive Depression” OR “Recurrent Depression” OR “Treatment Resistant Depression” OR “Depression (Emotion)” OR Anxiety OR “Anxiety Sensitivity” OR “Social Anxiety” OR “Speech Anxiety” OR “Anxiety Disorders” OR “Generalized Anxiety Disorder” OR “Obsessive Compulsive Disorder” OR “Panic Attack” OR “Panic Disorder” OR Phobias OR “Social Phobia” OR “Suicidal Behavior” OR “Attempted Suicide” OR Suicide OR “Suicidal Ideation” OR Suicidality OR Suicide OR “Self-Injurious Behavior” OR “Self-Inflicted Wounds” OR “Self-Mutilation”)

)

**Results:** 8,062

**Database: Web of Science**

(AB = (gay OR lesbian OR homosexual* OR bisexual* OR pansexual* OR sexuality OR "sexual minority" OR "sexual minorities" OR "sexual orientation" OR queer* OR "non-heterosexual" OR MSM OR WSW OR MSMW OR WSWM OR "men who have sex with men" OR "women who have sex with women" OR "men-loving men" OR "women-loving women" OR "same-gender loving" OR bicurious OR lesbigay OR transgender OR "gender minority" OR "gender minorities" OR "gender diverse" OR "gender identity" OR "gender identities" OR "two spirit" OR "two-spirit" OR "same sex couple" OR "same-sex couple" OR "same sex relationship" OR "same-sex relationship" OR "same sex relations" OR "same-sex relations" OR LGB OR LGBQ OR LGBT* OR GLB OR GLBT* OR asexual* OR demisexual* )

OR TI = (gay OR lesbian OR homosexual* OR bisexual* OR pansexual* OR sexuality OR "sexual minority" OR "sexual minorities" OR "sexual orientation" OR queer* OR "non-heterosexual" OR MSM OR WSW OR MSMW OR WSWM OR "men who have sex with men" OR "women who have sex with women" OR "men-loving men" OR "women-loving women" OR "same-gender loving" OR bicurious OR lesbigay OR transgender OR "gender minority" OR "gender minorities" OR "gender diverse" OR "gender identity" OR "gender identities" OR "two spirit" OR "two-spirit" OR "same sex couple" OR "same-sex couple" OR "same sex relationship" OR "same-sex relationship" OR "same sex relations" OR "same-sex relations" OR LGB OR LGBQ OR LGBT* OR GLB OR GLBT* OR asexual* OR demisexual*)

)

AND (AB = (family OR mother OR father OR maternal OR paternal OR sibling OR brother OR sister OR son OR daughter OR child* OR partner OR spouse OR boyfriend OR girlfriend OR husband OR wife OR peers OR friend* OR companion* OR colleagues OR school OR work* OR bully* OR homophob* OR transphobe* OR "anti-homosexual" OR "anti-gay" OR "anti-transgender" OR neglect OR abuse OR rejection OR "psychosocial support" OR "support system" OR "social care" OR "Social Support" OR "Intergenerational Relations" OR "Interprofessional Relations" OR "Social Integration" OR "Social Cohesion" OR “social determinant” OR “social determinants”)

OR TI = (family OR mother OR father OR maternal OR paternal OR sibling OR brother OR sister OR son OR daughter OR child* OR partner OR spouse OR boyfriend OR girlfriend OR husband OR wife OR peers OR friend* OR companion* OR colleagues OR school OR work* OR bully* OR homophob* OR transphobe* OR "anti-homosexual" OR "anti-gay" OR "anti-transgender" OR neglect OR abuse OR rejection OR "psychosocial support" OR "support system" OR "social care"

OR "Social Support" OR "Intergenerational Relations" OR "Interprofessional Relations" OR "Social Integration" OR "Social Cohesion" OR “social determinant” OR “social determinants”)

)

AND (AB = (depress* OR dysthym* OR melancholia OR anxi* OR "internalizing disorder" OR "internalizing disorders" OR "mental disorder" OR "mental disorders" OR "mental health" OR "chronic mental illness" OR "affective disorders" OR melancholia* OR "self-injury" OR "self injury" OR "self-injurious" OR "self injurious" OR "self-harm" OR "self harm" OR NSSI OR suicid*)

OR TI = (depress* OR dysthym* OR melancholia OR anxi* OR "internalizing disorder" OR "internalizing disorders" OR "mental disorder" OR "mental disorders" OR "mental health" OR "chronic mental illness" OR "affective disorders" OR melancholia* OR "self-injury" OR "self injury" OR "self-injurious" OR "self injurious" OR "self-harm" OR "self harm" OR NSSI OR suicid* )

)

**Results:** 7,731

**S1b. Journals reviewed:**

- Archives of Sexual Behavior
- Journal of Adolescent Health
- Journal of Affective Disorders
- Journal of the American Academy of Child and Adolescent Psychiatry
- Journal of Bisexuality
- Journal of Consulting and Clinical Psychology
- Journal of LGBT Issues in Counseling
- Journal of LGBT Youth
- Journal of Gay and Lesbian Mental Health
- Journal of Gay & Lesbian Social Services: Issues in Practice, Policy & Research
- Journal of GLBT Family Studies
- Journal of Homosexuality
- Journal of Lesbian Studies
- Journal of Psychopathology and Clinical Science (Journal of Abnormal Psychology)
- Journal of Sexual Medicine
- Journal of Sex Research
- LGBT Health
- International Journal of Transgender Health
- International Journal of Transgenderism (from 2005 on)
- Psychology & Sexuality
- Psychology of Sexual Orientation and Gender Diversity
- Psychology of Women Quarterly
- Sexual and Relationship Therapy
- Transgender Health

**S1c. Articles reviewed for citations:**


- Russell, S. T., & Fish, J. N. (2016). Mental health in lesbian, gay, bisexual, and transgender (LGBT) youth. Annual review of clinical psychology, 12, 465-487.
- Richardson, V. E., & King, S. D. (2017). Mental health for older LGBT adults. Annual Review of Gerontology and Geriatrics, 37(1), 59-75.
- Meyer, I. H. (2003). Prejudice, social stress, and mental health in lesbian, gay, and bisexual populations: conceptual issues and research evidence. Psychological bulletin, 129(5), 674.
- Schwartz, S., & Meyer, I. H. (2010). Mental health disparities research: The impact of within and between group analyses on tests of social stress hypotheses. Social science & medicine, 70(8), 1111-1118.
- Hatzenbuehler, M. L. (2009). How does sexual minority stigma “get under the skin”? A psychological mediation framework. Psychological bulletin, 135(5), 707.
- Pachankis, J. E. (2007). The psychological implications of concealing a stigma: a cognitive-affective-behavioral model. Psychological bulletin, 133(2), 328.
- Peplau, L. A., & Fingerhut, A. W. (2007). The close relationships of lesbians and gay men. Annu. Rev. Psychol., 58, 405-424.
- Herek, G. M., & Garnets, L. D. (2007). Sexual orientation and mental health. Annu. Rev. Clin. Psychol., 3, 353-375.
- Lefevor, G. T., Davis, E. B., Paiz, J. Y., & Smack, A. C. (2021). The relationship between religiousness and health among sexual minorities: A meta-analysis. Psychological bulletin, 147(7), 647.
- Scheim, A. I., Baker, K. E., Restar, A. J., & Sell, R. L. (2022). Health and Health Care Among Transgender Adults in the United States. Annual Review of Public Health, 43, 503-523.
- Moore, M. R., & Stambolis-Ruhstorfer, M. (2013). LGBT sexuality and families at the start of the twenty-first century. Annual Review of Sociology, 39, 491-507.
- Orel, N. A. (2017). Families and support systems of LGBT elders. Annual Review of Gerontology and Geriatrics, 37(1), 89-109.
- Coker, T. R., Austin, S. B., & Schuster, M. A. (2010). The health and health care of lesbian, gay, and bisexual adolescents. Annual review of public health, 31, 457-477.
- Ryan, D., & Martin, A. (2000). Lesbian, gay, bisexual, and transgender parents in the school systems. School psychology review, 29(2), 207-216.
- McCabe, P. C., & Rubinson, F. (2008). Committing to social justice: The behavioral intention of school psychology and education trainees to advocate for lesbian, gay, bisexual, and transgender youth. School Psychology Review, 37(4), 469-486.

- Newcomb, M. E., & Mustanski, B. (2010). Internalized homophobia and internalizing mental health problems: A meta-analytic review. Clinical psychology review, 30(8), 1019-1029.
- Henning-Stout, M., James, S., & Macintosh, S. (2000). Reducing harassment of lesbian, gay, bisexual, transgender, and questioning youth in schools. School Psychology Review, 29(2), 180-191.

## Supplemental Material 2. Studies included in the meta-analysis

### S2a. Descriptive information of included studies

| Study | N | Source | Structural Stigma | Age (Mean) | % Gender Minority | % Bisexual | Social Support | SGM-Specific | Psychopathology | Effect Size (*r*) |
|---|---|---|---|---|---|---|---|---|---|---|
| Abreu (2023) | 1292 | Journal Article | - | 15.65 | .15 | .56 | Family (Parents) | Y | Depression | -.21 |
| Al-Khouja (2021) | 882 | Dissertation | ESS | 38 | .00 | .46 | Partner | N | Depression/ Anxiety | -.31 |
| | | | | | | | Family | N | Depression/ Anxiety | -.17 |
| | | | | | | | Peers | N | Depression/ Anxiety | -.15 |
| Algarin (2024) | 152 | Journal Article | - | NA | 1.00 | .00 | General | Y | Depression | -.23 |
| Allen (2014) | 117 | Journal Article | - | 45.77 | .00 | .00 | General | N | Depression | -.37 |
| Alpert (2024) | 78 | Dissertation | - | 31.89 | .13 | .53 | General | Y | Depression | -.53 |

| Study | N | Source | Structural Stigma | Age (Mean) | % Gender Minority | % Bisexual | Social Support | SGM-Specific | Psychopathology | Effect Size (*r*) |
|---|---|---|---|---|---|---|---|---|---|---|
| Aristegui (2021) | 61 | Journal Article | - | 29.87 | 1.00 | .00 | General | N | Depression | -.33 |
| | | | | | | | Peers | N | Depression | .28 |
| | | | | | | | Family | N | Depression | -.35 |
| | | | | | | | General | N | Anxiety | -.26 |
| | | | | | | | Peers | N | Anxiety | .08 |
| | | | | | | | Family | N | Anxiety | -.20 |
| Arnett (2019) | 387 | Journal Article | GSS | 29.48 | .06 | 1.00 | General | Y | Depression | -.32 |
| | | | | | | | General | Y | Anxiety | -.38 |
| | | | | | | | Peers (SGM) | Y | Depression | -.25 |
| | | | | | | | Peers (SGM) | Y | Anxiety | -.31 |

| Study | N | Source | Structural Stigma | Age (Mean) | % Gender Minority | % Bisexual | Social Support | SGM-Specific | Psychopathology | Effect Size ($r$) |
|---|---|---|---|---|---|---|---|---|---|---|
| Ayala (2000) | 117 | Journal Article | - | NA | .00 | .00 | Family | N | Depression | -.36 |
| | | | | | | | Peers | N | Depression | -.47 |
| | | | | | | | General | Y | Depression/ Anxiety | -.38 |
| Bartoshuk (2008) | 180 | Dissertation | GSS | 35.3 | .00 | .15 | Family | N | Depression | -.22 |
| | | | | | | | Peers | N | Depression | -.34 |
| | | | | | | | Partner | N | Depression | -.49 |
| | | | | | | | General | N | Depression | -.42 |
| Bazargan (2012) | 220 | Journal Article | GSS | 36 | 1.00 | .00 | General | Y | Depression | -.39 |
| Beggiato (2025) | 152 | Dissertation | - | 28.63 | .73 | .29 | General | N | Depression | -.28 |
| | | | | | | | General | N | Anxiety | -.16 |
| | | | | | | | General | N | Suicidality | .19 |

| Study | N | Source | Structural Stigma | Age (Mean) | % Gender Minority | % Bisexual | Social Support | SGM-Specific | Psychopathology | Effect Size (*r*) |
|---|---|---|---|---|---|---|---|---|---|---|
| Benibgui (2011) | 63 | Dissertation | - | 21.3 | .00 | .13 | General | Y | Suicidality | .00 |
| | | | | | | | General | Y | Depression | -.31 |
| Berg (2017) | 129 | Contact | GSS | 45 | .00 | .10 | General | N | Depression | -.41 |
| Bergfeld (2017) | 175 | Journal Article | - | 22.84 | .00 | .03 | General | Y | Depression | -.30 |
| Berlin (2008) | 75 | Dissertation | GSS | 56.39 | .00 | .09 | General | N | Depression | -.28 |
| Bhoopsingh (2017) | 410 | Dissertation | GSS | 21.29 | .00 | .23 | Family | N | Depression | -.38 |
| | | | | | | | Peers | N | Depression | -.46 |
| | | | | | | | School | Y | Depression | -.16 |
| Bitran (2024) | 60 | Journal Article | - | 16.88 | .17 | .67 | Family | N | Depression | -.25 |
| Bittner (2016) | 169 | Dissertation | GSS | 32 | .00 | .60 | General | N | Depression | -.42 |
| | | | | | | | General | N | Anxiety | -.21 |
| | | | | | | | General | N | Suicidality | -.36 |

| Study | N | Source | Structural Stigma | Age (Mean) | % Gender Minority | % Bisexual | Social Support | SGM-Specific | Psychopathology | Effect Size (*r*) |
|---|---|---|---|---|---|---|---|---|---|---|
| | 134 | Dissertation | GSS | 32 | 1.00 | .52 | General | N | Depression | -.37 |
| | | | | | | | General | N | Anxiety | -.22 |
| | | | | | | | General | N | Suicidality | -.18 |
| Bolwell (2022) | 396 | Dissertation | GSS | NA | .07 | .60 | General | Y | Depression | -.48 |
| Boyd (2021) | 448 | Journal Article | GSS | 22.3 | .00 | .00 | General | N | Depression | -.25 |
| | | | | | | | Family | Y | Depression | -.19 |
| Boyd, Jones (2024) | 400 | Journal Article | - | 23.46 | .00 | .20 | Family | N | Depression | .00 |
| | | | | | | | Family | N | Suicidality | -.03 |
| Boyd, Quinn (2024) | 225 | Journal Article | - | 29 | .00 | .00 | General | N | Depression | -.95 |
| Brady (2024) | 55 | Dissertation | - | 25.4 | .00 | .27 | General | N | Depression/ Anxiety | -.29 |

| Study | N | Source | Structural Stigma | Age (Mean) | % Gender Minority | % Bisexual | Social Support | SGM-Specific | Psychopathology | Effect Size (*r*) |
|---|---|---|---|---|---|---|---|---|---|---|
| Budge (2013) | 64 | Journal Article | - | 40.28 | 1.00 | .42 | General | N | Anxiety | -.28 |
| | | | | | | | General | N | Depression | -.41 |
| Budge (2014) | 64 | Journal Article | GSS | 30.3 | 1.00 | .96 | General | N | Depression | -.34 |
| | | | | | | | General | N | Anxiety | -.30 |
| Burish (2023) | 139 | Journal Article | - | 33.78 | .00 | .71 | Peers | N | Suicidality | -.22 |
| | | | | | | | Family | N | Suicidality | -.19 |
| Cain (2017) | 1071 | Dissertation | GSS | 40.24 | .00 | .05 | General | N | Depression | -.37 |
| Carter (2014) | 165 | Journal Article | GSS | 37.89 | .00 | .12 | Work | Y | Depression/ Anxiety | -.28 |
| Cassidy (2021) | 236 | Dissertation | GSS | NA | 1.21 | .82 | Family | Y | Depression/ Anxiety | -.12 |

| Study | N | Source | Structural Stigma | Age (Mean) | % Gender Minority | % Bisexual | Social Support | SGM-Specific | Psychopathology | Effect Size (*r*) |
|---|---|---|---|---|---|---|---|---|---|---|
| | | | | | | | Family (Parents) | Y | Depression/ Anxiety | -.15 |
| Cerezo (2024) | 465 | Journal Article | - | 29.46 | .00 | .00 | General | Y | Depression | -.15 |
| | | | | | | | General | Y | Anxiety | -.13 |
| Chakrapani (2017) | 300 | Journal Article | - | 29.6 | 1.00 | .00 | General | N | Depression | -.38 |
| Chang (2021) | 256 | Journal Article | GSS | 27.89 | .11 | .61 | General | N | Depression | -.41 |
| Chang (2023) | 161 | Journal Article | GSS | NA | .00 | .40 | General | N | Depression | -.35 |
| | | | | | | | General | N | Anxiety | -.13 |
| Chelliah (2024) | 139 | Contact | - | 15.3 | .90 | .41 | Family (Parents) | Y | Depression | -.34 |

| Study | N | Source | Structural Stigma | Age (Mean) | % Gender Minority | % Bisexual | Social Support | SGM-Specific | Psychopathology | Effect Size ($r$) |
|---|---|---|---|---|---|---|---|---|---|---|
| | | | | | | | Family (Parents) | Y | Anxiety | -.26 |
| | | | | | | | Peers | N | Depression | -.16 |
| | | | | | | | Peers | N | Anxiety | -.08 |
| Chen (2020) | 153 | Journal Article | - | 20.97 | .00 | .39 | General | N | Depression | -.31 |
| Cheung (2025a) | 263 | Contact | - | 53.4 | .00 | - | General | N | Depression | -.13 |
| | | | | | | | General | N | Anxiety | -.00 |
| Cheung (2025b) | 214 | Contact | - | NA | .00 | .05 | General | N | Depression | -.07 |
| | | | | | | | General | N | Suicidality | .07 |
| Chinazzo (2023) | 213 | Contact | - | 18.53 | 1.00 | .00 | General | N | Depression | -.31 |
| Christie (2020) | 33 | Journal Article | - | 19.7 | .00 | .00 | General | N | Anxiety | -.13 |

| Study | N | Source | Structural Stigma | Age (Mean) | % Gender Minority | % Bisexual | Social Support | SGM-Specific | Psychopathology | Effect Size ($r$) |
|---|---|---|---|---|---|---|---|---|---|---|
| Clark (2014) | 96 | Contact | - | NA | 1.00 | .00 | Family (Parents) | N | Depression | -.23 |
| Craney (2018) | 352 | Journal Article | GSS | 27.14 | .09 | 1.00 | General | Y | Depression/ Anxiety | -.29 |
| Dai (2023) | 1394 | Journal Article | - | NA | .00 | .00 | General | N | Depression | -.23 |
| Das (2023) | 106 | Journal Article | - | 33.35 | .59 | .72 | Family | N | Depression | -.52 |
| | | | | | | | Peers | N | Depression | .06 |
| | | | | | | | Partner | N | Depression | -.42 |
| | | | | | | | Family | N | Anxiety | -.25 |
| | | | | | | | Peers | N | Anxiety | .09 |
| | | | | | | | Partner | N | Anxiety | -.21 |
| Del Pino (2023) | 434 | Contact | - | 22.1 | 0 | 0 | Family | N | Depression | -.16 |

| Study | N | Source | Structural Stigma | Age (Mean) | % Gender Minority | % Bisexual | Social Support | SGM-Specific | Psychopathology | Effect Size (*r*) |
|---|---|---|---|---|---|---|---|---|---|---|
| DeLaney (2020) | 234 | Journal Article | - | 18.46 | .00 | .76 | General | N | Depression | -.31 |
| DeLaney (2023) | 179 | Journal Article | GSS | 19.48 | .22 | .80 | General | N | Depression | -.28 |
| | | | | | | | General | N | Anxiety | -.18 |
| | | | | | | | General | N | Anxiety | -.19 |
| Dejanipont (2023) | 100 | Journal Article | - | 59 | .00 | .00 | General | N | Depression | -.34 |
| Dellucci (2025) | 50 | Journal Article | - | 18.06 | .00 | .42 | Partner | N | Depression | .25 |
| | | | | | | | Partner | N | Anxiety | .06 |
| | | | | | | | Family | Y | Depression | -.49 |
| | | | | | | | Family | Y | Anxiety | -.33 |

| Study | N | Source | Structural Stigma | Age (Mean) | % Gender Minority | % Bisexual | Social Support | SGM-Specific | Psychopathology | Effect Size (*r*) |
|---|---|---|---|---|---|---|---|---|---|---|
| Detwiler (2016) | 189 | Dissertation | GSS | 60.41 | .00 | .41 | General | Y | Depression/ Anxiety | -.36 |
| | 189 | Dissertation | GSS | 60.41 | .00 | .41 | General | N | Depression/ Anxiety | -.34 |
| Dickenson (2016) | 519 | Journal Article | GSS | 17.42 | .00 | .36 | Family | N | Depression | -.36 |
| Ding (2020) | 715 | Journal Article | - | 27.09 | .00 | .27 | General | N | Depression | -.34 |
| Do (2022) | 206 | Journal Article | - | 33.7 | .00 | .19 | General | Y | Depression | -.26 |
| | | | | | | | General | N | Anxiety | -.23 |
| Dolezal (2023) | 234 | Journal Article | GSS | 20.17 | .72 | .93 | General | Y | Depression | -.23 |
| | | | | | | | General | Y | Anxiety | -.37 |

| Study | N | Source | Structural Stigma | Age (Mean) | % Gender Minority | % Bisexual | Social Support | SGM-Specific | Psychopathology | Effect Size (*r*) |
|---|---|---|---|---|---|---|---|---|---|---|
| Donnelly (2019) | 246 | Contact | GSS | 49.83 | .00 | .00 | Family | N | Depression | -.19 |
| | | | | | | | Partner | N | Depression | -.30 |
| | 342 | Contact | GSS | 49.41 | .00 | .00 | Family | N | Depression | -.20 |
| | | | | | | | Partner | N | Depression | -.25 |
| Dowshen (2009) | 42 | Journal Article | GSS | NA | .14 | .00 | General | N | Depression/ Anxiety | -.04 |
| Drydakis (2021) | 866 | Journal Article | ESS | 32.63 | .00 | .00 | Peers | Y | Depression | -.44 |
| | | | | | | | Family | Y | Depression | -.21 |
| Du (2018) | 321 | Journal Article | - | 30.64 | .00 | .03 | General | N | Depression | -.36 |
| Dyar (2014) | 106 | Journal Article | - | 25.1 | .00 | 1.00 | Peers (Non-SGM) | Y | Depression | -.13 |
| | | | | | | | Peers (SGM) | Y | Depression | -.19 |

| Study | N | Source | Structural Stigma | Age (Mean) | % Gender Minority | % Bisexual | Social Support | SGM-Specific | Psychopathology | Effect Size (*r*) |
|---|---|---|---|---|---|---|---|---|---|---|
| Dyar (2020) | 488 | Journal Article | - | 20.06 | .26 | .76 | General | Y | Depression | -.34 |
| | | | | | | | General | Y | Anxiety | -.30 |
| Dyar (2021) | 414 | Journal Article | - | 20.21 | .29 | 1.00 | General | Y | Depression | -.27 |
| | | | | | | | General | Y | Anxiety | -.30 |
| Eadeh (2023) | 155 | Journal Article | GSS | 27.84 | .69 | .52 | General | Y | Depression | -.50 |
| | | | | | | | General | Y | Anxiety | -.34 |
| Earle (2000) | 82 | Dissertation | - | 28.82 | .00 | .24 | General | N | Depression | -.50 |
| Edwards (2020) | 106 | Journal Article | GSS | 29.17 | .06 | .00 | General | N | Suicidality | -.16 |
| EngHui (2025) | 317 | Journal Article | - | 23.62 | .27 | .49 | Family | N | Suicidality | -.33 |

| Study | N | Source | Structural Stigma | Age (Mean) | % Gender Minority | % Bisexual | Social Support | SGM-Specific | Psychopathology | Effect Size (*r*) |
|---|---|---|---|---|---|---|---|---|---|---|
| | | | | | | | Peers | N | Suicidality | -.16 |
| | | | | | | | Partner | N | Suicidality | -.11 |
| Feinstein (2014) | 414 | Journal Article | GSS | 31.3 | .00 | .00 | Family | N | Depression | -.33 |
| Feinstein (2016) | 335 | Journal Article | GSS | 43.46 | .00 | .00 | General | Y | Depression | -.01 |
| | | | | | | | General | Y | Anxiety | -.05 |
| | 242 | Journal Article | GSS | 43.46 | .00 | 1.00 | General | Y | Depression | .02 |
| | | | | | | | General | Y | Anxiety | -.04 |
| Fiani (2019) | 342 | Dissertation | - | 30.02 | .63 | .71 | General | N | Depression | -.27 |
| | | | | | | | General | N | Anxiety | -.21 |
| Fingerhut (2018) | 89 | Journal Article | GSS | 36.78 | .00 | .00 | Peers | N | Depression | -.54 |

| Study | N | Source | Structural Stigma | Age (Mean) | % Gender Minority | % Bisexual | Social Support | SGM-Specific | Psychopathology | Effect Size (*r*) |
|---|---|---|---|---|---|---|---|---|---|---|
| | | | | | | | Family | N | Depression | -.35 |
| Fischer (2011) | 1504 | Dissertation | GSS | 16.3 | .10 | .35 | School | Y | Anxiety | -.22 |
| | | | | | | | School | N | Depression | -.26 |
| Frank (2017) | 127 | Dissertation | GSS | 18.69 | 1.01 | .39 | Peers | N | Suicidality | -.29 |
| | | | | | | | Family (Parents) | N | Suicidality | -.20 |
| | | | | | | | School | N | Suicidality | -.19 |
| | | | | | | | Peers | N | Depression | -.35 |
| | | | | | | | Family (Parents) | N | Depression | -.29 |
| | | | | | | | School | N | Depression | -.27 |
| Galarneau (2025) | 355 | Journal Article | - | 21.07 | .24 | .80 | General | N | Depression | -.35 |
| | | | | | | | General | N | Anxiety | -.27 |

| Study | N | Source | Structural Stigma | Age (Mean) | % Gender Minority | % Bisexual | Social Support | SGM-Specific | Psychopathology | Effect Size (*r*) |
|---|---|---|---|---|---|---|---|---|---|---|
| Gato (2020) | 403 | Journal Article | - | 22.13 | .13 | .42 | Family | Y | Depression | -.25 |
| | | | | | | | | Y | Anxiety | -.19 |
| Gillig (2022) | 41 | Contact | GSS | 15.5 | .67 | .72 | General | N | Depression | .02 |
| | | | | | | | | N | Anxiety | .21 |
| Gilmore (1996) | 105 | Dissertation | - | NA | .00 | .29 | General | NA | Depression | -.34 |
| | | | | | | | General | NA | Anxiety | -.20 |
| Glenwright (2023) | 775 | Dissertation | - | 22.06 | .55 | .84 | General | N | Depression | -.37 |
| | | | | | | | General | N | Anxiety | -.37 |
| Glynn (2016) | 573 | Journal Article | GSS | 35.1 | 1.00 | .00 | Family | N | Depression | -.21 |
| Golembiewski (2023) | 268 | Dissertation | GSS | 19.8 | .24 | .52 | General | N | Depression/ Anxiety | -.12 |

| Study | N | Source | Structural Stigma | Age (Mean) | % Gender Minority | % Bisexual | Social Support | SGM-Specific | Psychopathology | Effect Size (*r*) |
|---|---|---|---|---|---|---|---|---|---|---|
| Gray (1999) | 35 | Journal Article | GSS | 37 | .00 | .00 | General | N | Depression | -.36 |
| Greenblatt (2019) | 309 | Dissertation | - | 24.32 | 1.00 | .00 | Peers | N | Depression/ Anxiety | -.31 |
| | | | | | | | Family | N | Depression/ Anxiety | -.30 |
| | | | | | | | Partner | N | Depression/ Anxiety | -.32 |
| | | | | | | | Peers | N | Depression | -.46 |
| | | | | | | | Family | N | Depression | -.37 |
| | | | | | | | Partner | N | Depression | -.39 |
| | | | | | | | Peers | N | Anxiety | -.19 |
| | | | | | | | Family | N | Anxiety | -.22 |
| | | | | | | | Partner | N | Anxiety | -.21 |

| Study | N | Source | Structural Stigma | Age (Mean) | % Gender Minority | % Bisexual | Social Support | SGM-Specific | Psychopathology | Effect Size (*r*) |
|---|---|---|---|---|---|---|---|---|---|---|
| | | | | | | | Peers | N | Suicidality | -.23 |
| | | | | | | | Family | N | Suicidality | -.24 |
| | | | | | | | Partner | N | Suicidality | -.14 |
| Grigoriou (2014) | 142 | Journal Article | - | 40.37 | .00 | .00 | General | Y | Depression | -.35 |
| | | | | | | | General | Y | Anxiety | -.37 |
| Grocott (2023) | 191 | Journal Article | - | 30.28 | .80 | .45 | Partner | N | Depression | -.15 |
| | | | | | | | Family | N | Depression | -.07 |
| | | | | | | | Peers | N | Depression | -.13 |
| Grossman (2021) | 58 | Journal Article | - | 18 | 1.00 | .00 | Family (Parents) | Y | Depression | -.33 |
| | 71 | Journal Article | - | 18 | 1.00 | .00 | Family (Parents) | N | Depression | -.27 |

| Study | N | Source | Structural Stigma | Age (Mean) | % Gender Minority | % Bisexual | Social Support | SGM-Specific | Psychopathology | Effect Size (*r*) |
|---|---|---|---|---|---|---|---|---|---|---|
| Guo (2025) | 419 | Journal Article | - | 22.63 | 1.00 | .00 | Family | N | Suicidality | -.39 |
| | | | | | | | Peers | N | Suicidality | -.28 |
| | | | | | | | General | N | Suicidality | -.32 |
| Gutiérrez (2022) | 235 | Dissertation | - | 21.31 | .06 | .62 | Family (Parents) | Y | Depression | -.16 |
| Guzman Parra (2023) | 204 | Journal Article | ESS | 27.95 | 1.00 | .04 | General | N | Suicidality | -.01 |
| Haas (2019) | 1303 | Journal Article | - | 39.06 | .05 | .13 | Partner | N | Depression | -.22 |
| | | | | | | | Peers | N | Depression | -.24 |
| | | | | | | | Family | N | Depression | -.26 |
| Hammack (2024) | 314 | Contact | - | 16.5 | .32 | .61 | General | N | Depression | -.30 |
| Handelman (2017) | 220 | Dissertation | - | 21 | .00 | .00 | General | Y | Depression | -.33 |

| Study | N | Source | Structural Stigma | Age (Mean) | % Gender Minority | % Bisexual | Social Support | SGM-Specific | Psychopathology | Effect Size (*r*) |
|---|---|---|---|---|---|---|---|---|---|---|
| | | | | | | | General | Y | Anxiety | -.11 |
| Hatchel (2019) | 934 | Journal Article | - | 15.91 | .00 | .44 | School | N | Suicidality | -.14 |
| Heiden-Rootes (2021) | 153 | Journal Article | - | 29.67 | .20 | 1.00 | Family (Parents) | Y | Depression | -.15 |
| | | | | | | | Family (Siblings) | Y | Depression | -.15 |
| | | | | | | | Peers | Y | Depression | -.14 |
| | 231 | Journal Article | - | 35.58 | .04 | .00 | Family (Parents) | Y | Depression | -.28 |
| | | | | | | | Family (Siblings) | Y | Depression | -.28 |
| | | | | | | | Peers | Y | Depression | -.22 |

| Study | N | Source | Structural Stigma | Age (Mean) | % Gender Minority | % Bisexual | Social Support | SGM-Specific | Psychopathology | Effect Size (*r*) |
|---|---|---|---|---|---|---|---|---|---|---|
| Hershberger (1995) | 165 | Journal Article | - | 19.08 | .00 | .00 | Family | Y | Suicidality | .17 |
| | | | | | | | Family | Y | Depression | -.03 |
| Hill (2017) | 50 | Journal Article | - | 20.84 | .00 | .00 | Family | N | Suicidality | -.32 |
| | | | | | | | Peers (Non-SGM) | N | Suicidality | -.27 |
| | | | | | | | Peers (SGM) | Y | Suicidality | -.21 |
| | | | | | | | Family | N | Depression | -.26 |
| | | | | | | | Peers (Non-SGM) | N | Depression | -.45 |
| | | | | | | | Peers (SGM) | Y | Depression | -.42 |

| Study | N | Source | Structural Stigma | Age (Mean) | % Gender Minority | % Bisexual | Social Support | SGM-Specific | Psychopathology | Effect Size ($r$) |
|---|---|---|---|---|---|---|---|---|---|---|
| Horne (2022) | 523 | Journal Article | - | 27.75 | .72 | .68 | General | Y | Depression | -.39 |
| | | | | | | | General | Y | Anxiety | -.27 |
| Houston (2007) | 817 | Contact | - | 33 | .00 | .13 | General | N | Depression | -.07 |
| Huebner (2014) | 1289 | Journal Article | - | NA | .00 | .00 | Peers (SGM) | Y | Depression | -.01 |
| Jackson (1999) | 134 | Dissertation | - | NA | .01 | .24 | Family (Parents) | Y | Depression | -.34 |
| | | | | | | | Peers (SGM) | Y | Depression | -.32 |
| | | | | | | | Peers (Non-SGM) | Y | Depression | -.35 |
| | | | | | | | Family (Parents) | Y | Suicidality | -.22 |

| Study | N | Source | Structural Stigma | Age (Mean) | % Gender Minority | % Bisexual | Social Support | SGM-Specific | Psychopathology | Effect Size ($r$) |
|---|---|---|---|---|---|---|---|---|---|---|
| | | | | | | | Peers (SGM) | Y | Suicidality | -.34 |
| | | | | | | | Peers (SGM) | Y | Suicidality | -.23 |
| Jeon (2024) | 224 | Data | - | 19.85 | .27 | .69 | Work | Y | Depression | -.09 |
| | | | | | | | Work | Y | Suicidality | -.10 |
| | | | | | | | Work | Y | Anxiety | -.09 |
| | | | | | | | Peers | Y | Depression | -.25 |
| | | | | | | | Peers | Y | Suicidality | -.06 |
| | | | | | | | Peers | Y | Anxiety | -.23 |
| | 46 | Data | - | 19.85 | .87 | .67 | Work | Y | Depression | -.08 |
| | | | | | | | Work | Y | Suicidality | -.20 |
| | | | | | | | Work | Y | Anxiety | .06 |
| | | | | | | | Peers | Y | Depression | -.35 |

| Study | N | Source | Structural Stigma | Age (Mean) | % Gender Minority | % Bisexual | Social Support | SGM-Specific | Psychopathology | Effect Size (*r*) |
|---|---|---|---|---|---|---|---|---|---|---|
| | | | | | | | Peers | Y | Suicidality | -.18 |
| | | | | | | | Peers | Y | Anxiety | -.30 |
| Johnson (2001) | 103 | Journal Article | - | 38 | .00 | .00 | General | N | Depression | -.30 |
| Jones (2011) | 1823 | Dissertation | - | 41 | .00 | .00 | General | N | Depression | -.27 |
| | | | | | | | General | Y | Depression | -.16 |
| | | | | | | | Family | N | Depression | -.21 |
| Jones (2012) | 279 | Dissertation | - | 22.5 | .00 | .25 | General | N | Suicidality | -.16 |
| | | | | | | | General | N | Depression | -.31 |
| Jorgensen (2012) | 207 | Dissertation | - | 30.92 | .28 | 1.00 | Peers (SGM) | Y | Depression/ Anxiety | -.12 |
| | | | | | | | General | N | Depression/ Anxiety | -.06 |

| Study | N | Source | Structural Stigma | Age (Mean) | % Gender Minority | % Bisexual | Social Support | SGM-Specific | Psychopathology | Effect Size (*r*) |
|---|---|---|---|---|---|---|---|---|---|---|
| Kamen (2017) | 201 | Journal Article | - | 53 | .00 | .14 | General | N | Anxiety | -.20 |
| | | | | | | | General | N | Depression | -.27 |
| Kaplan (2019) | 902 | Journal Article | - | 32.47 | .70 | .00 | Peers (SGM) | Y | Anxiety | -.18 |
| Kaplan (2020) | 136 | Dissertation | - | 36.88 | .85 | .54 | Peers (SGM) | Y | Depression | .05 |
| | | | | | | | Peers (Non-SGM) | N | Depression | -.13 |
| | | | | | | | Peers (SGM) | Y | Anxiety | .09 |
| | | | | | | | Peers (Non-SGM) | N | Anxiety | .13 |

| Study | N | Source | Structural Stigma | Age (Mean) | % Gender Minority | % Bisexual | Social Support | SGM-Specific | Psychopathology | Effect Size (*r*) |
|---|---|---|---|---|---|---|---|---|---|---|
| Katz-Wise (2021) | 30 | Journal Article | - | 15.1 | .99 | .83 | Family | N | Depression | -.23 |
| | | | | | | | General | N | Depression | -.33 |
| | | | | | | | Peers (SGM) | Y | Depression | -.10 |
| | | | | | | | Family | N | Anxiety | -.09 |
| | | | | | | | General | N | Anxiety | -.19 |
| | | | | | | | Peers (SGM) | Y | Anxiety | .08 |
| Kavanaugh (2019) | 941 | Journal Article | - | 21.4 | .04 | .40 | Family | Y | Depression | -.22 |
| Kecojevic (2019) | 191 | Journal Article | - | NA | .00 | .00 | Family | N | Depression | -.13 |
| | | | | | | | Peers | N | Depression | -.14 |

| Study | N | Source | Structural Stigma | Age (Mean) | % Gender Minority | % Bisexual | Social Support | SGM-Specific | Psychopathology | Effect Size (*r*) |
|---|---|---|---|---|---|---|---|---|---|---|
| | | | | | | | Partner | N | Depression | -.13 |
| Keleher (2010) | 163 | Journal Article | - | 30 | .00 | .00 | General | N | Depression | -.53 |
| Kephart (2006) | 52 | Dissertation | - | 21.37 | .00 | .73 | General | N | Suicidality | -.28 |
| | | | | | | | General | N | Depression | -.48 |
| Kim (2019) | 295 | Journal Article | - | 32.77 | .08 | .54 | Partner | N | Anxiety | -.13 |
| | | | | | | | Family | N | Anxiety | -.23 |
| | | | | | | | Work | Y | Anxiety | -.33 |
| | | | | | | | Partner | N | Depression | -.24 |
| | | | | | | | Family | N | Depression | -.28 |
| | | | | | | | Work | Y | Depression | -.37 |

| Study | N | Source | Structural Stigma | Age (Mean) | % Gender Minority | % Bisexual | Social Support | SGM-Specific | Psychopathology | Effect Size ($r$) |
|---|---|---|---|---|---|---|---|---|---|---|
| Kishore (2016) | 125 | Dissertation | - | NA | .16 | .55 | Family (Parents) | Y | Depression | -.23 |
| | | | | | | | Family (Parents) | Y | Anxiety | -.14 |
| Knutson (2021) | 93 | Contact | GSS | NA | .00 | .00 | General | N | Depression | -.40 |
| Knutson (2021) | 27 | Contact | GSS | NA | .00 | 1.00 | General | N | Depression | -.43 |
| Kolp (2020) | 191 | Journal Article | GSS | 30.28 | .97 | .76 | Peers | Y | Depression | -.32 |
| | | | | | | | Peers | Y | Anxiety | -.29 |
| Kulick (2017) | 111 | Journal Article | GSS | 22.05 | .04 | .00 | General | Y | Depression | -.29 |
| | 349 | Journal Article | GSS | 23.09 | .02 | .00 | General | Y | Depression | -.28 |

| Study | N | Source | Structural Stigma | Age (Mean) | % Gender Minority | % Bisexual | Social Support | SGM-Specific | Psychopathology | Effect Size (*r*) |
|---|---|---|---|---|---|---|---|---|---|---|
| Lampis (2020) | 235 | Journal Article | - | NA | .00 | .00 | General | Y | Depression/ Anxiety | -.40 |
| Lee (2023) | 331 | Journal Article | - | 29 | .00 | .00 | Family | N | Depression | -.26 |
| | | | | | | | Peers | N | Depression | -.26 |
| | | | | | | | Partner | N | Depression | -.30 |
| | | | | | | | Family | N | Anxiety | -.18 |
| | | | | | | | Peers | N | Anxiety | -.14 |
| | | | | | | | Partner | N | Anxiety | -.14 |
| Lefevor (2025) | 359 | Journal Article | - | 36.16 | .18 | .30 | General | N | Depression | -.24 |
| Lehavot (2011) | 1381 | Journal Article | GSS | 33.54 | .00 | .45 | General | Y | Depression | -.30 |
| | | | | | | | General | Y | Anxiety | -.26 |

| Study | N | Source | Structural Stigma | Age (Mean) | % Gender Minority | % Bisexual | Social Support | SGM-Specific | Psychopathology | Effect Size ($r$) |
|---|---|---|---|---|---|---|---|---|---|---|
| | | | | | | | Work | Y | Depression | -.12 |
| | | | | | | | Work | Y | Anxiety | -.13 |
| Li (2017) | 321 | Journal Article | - | NA | .00 | .12 | General | N | Depression | -.36 |
| Li (2018) | 292 | Dissertation | - | 32 | .00 | .35 | General | Y | Depression | -.23 |
| | | | | | | | General | Y | Anxiety | -.37 |
| | | | | | | | Peers | N | Depression | -.28 |
| | | | | | | | Peers | N | Anxiety | -.17 |
| | | | | | | | Family | N | Depression | -.30 |
| | | | | | | | Family | N | Anxiety | -.22 |
| Li (2023) | 1393 | Journal Article | - | 20 | .00 | .31 | Family | N | Depression | -.42 |
| | | | | | | | Peers | N | Depression | -.37 |
| | | | | | | | Partner | N | Depression | -.37 |

| Study | N | Source | Structural Stigma | Age (Mean) | % Gender Minority | % Bisexual | Social Support | SGM-Specific | Psychopathology | Effect Size (*r*) |
|---|---|---|---|---|---|---|---|---|---|---|
| | | | | | | | Family | N | Anxiety | -.31 |
| | | | | | | | Peers | N | Anxiety | -.31 |
| | | | | | | | Partner | N | Anxiety | -.27 |
| Liu (2023) | 655 | Journal Article | - | NA | .00 | .00 | General | N | Depression | .12 |
| Liu (2024) | 1109 | Journal Article | - | 19 | .00 | .00 | General | N | Depression | -.42 |
| Logie (2017) | 391 | Journal Article | - | 30.9 | .12 | .63 | General | N | Depression | -.34 |
| Macbeth (2022) | 427 | Journal Article | - | - | .08 | .58 | Family | Y | Depression | -.56 |
| Mann (2024) | 163 | Journal Article | GSS | 26.43 | .88 | .00 | General | N | Depression/ Anxiety | -.17 |

| Study | N | Source | Structural Stigma | Age (Mean) | % Gender Minority | % Bisexual | Social Support | SGM-Specific | Psychopathology | Effect Size (*r*) |
|---|---|---|---|---|---|---|---|---|---|---|
| Marsland (2021) | 194 | Journal Article | - | 32.69 | .01 | .35 | Peers | N | Depression | -.27 |
| | | | | | | | Family | N | Depression | -.40 |
| | | | | | | | Partner | N | Depression | -.32 |
| Mason (2017) | 436 | Journal Article | GSS | - | .00 | .00 | Family | N | Anxiety | -.30 |
| | | | | | | | Peers | N | Anxiety | -.30 |
| Matijczak (2020) | 134 | Journal Article | - | 19.31 | .30 | .79 | School | Y | Anxiety | -.32 |
| | | | | | | | School | Y | Depression | -.34 |
| | | | | | | | General | N | Anxiety | .17 |
| | | | | | | | General | N | Depression | -.39 |
| McCurdy (2023) | 536 | Journal Article | - | 18.98 | .05 | .42 | Family (Parents) | N | Depression | -.22 |

| Study | N | Source | Structural Stigma | Age (Mean) | % Gender Minority | % Bisexual | Social Support | SGM-Specific | Psychopathology | Effect Size (*r*) |
|---|---|---|---|---|---|---|---|---|---|---|
| McLaren (2015) | 82 | Journal Article | - | 16.54 | .00 | .23 | School | N | Depression | -.23 |
| | | | | | | | Peers | N | Depression | -.43 |
| McLemore (2018) | 410 | Journal Article | - | 30.27 | .70 | .46 | General | N | Depression | -.45 |
| | | | | | | | General | N | Anxiety | -.15 |
| | | | | | | | General | Y | Anxiety | -.05 |
| Mehrtens (2020) | 158 | Dissertation | - | 15.33 | .16 | .57 | General | N | Depression | -.35 |
| | | | | | | | General | N | Anxiety | -.26 |
| Mereish (2020) | 94 | Journal Article | - | 16.1 | .23 | .67 | Family | Y | Depression | -.29 |
| Meza (2021) | 424 | Journal Article | - | NA | 1.00 | .00 | Family | Y | Depression/ Anxiety | -.21 |
| | | | | | | | Family | Y | Suicidality | -.19 |

| Study | N | Source | Structural Stigma | Age (Mean) | % Gender Minority | % Bisexual | Social Support | SGM-Specific | Psychopathology | Effect Size ($r$) |
|---|---|---|---|---|---|---|---|---|---|---|
| Milton (2023) | 175 | Journal Article | - | 30.5 | .31 | .73 | Family | N | Depression | -.33 |
| | | | | | | | General | N | Depression | -.23 |
| Mo (2020) | 415 | Journal Article | - | 30.2 | .00 | .00 | General | N | Anxiety | -.22 |
| Moe (2023) | 41 | Contact | - | NA | .00 | .00 | Family | N | Depression | -.40 |
| | | | | | | | Peers | N | Depression | -.29 |
| | | | | | | | Partner | N | Depression | -.51 |
| | | | | | | | Family | N | Suicidality | -.37 |
| | | | | | | | Peers | N | Suicidality | -.12 |
| | | | | | | | Partner | N | Suicidality | -.23 |
| | 37 | Contact | - | NA | .00 | 1.00 | Family | N | Depression | -.03 |
| | | | | | | | Peers | N | Depression | -.14 |
| | | | | | | | Partner | N | Depression | -.01 |

| Study | N | Source | Structural Stigma | Age (Mean) | % Gender Minority | % Bisexual | Social Support | SGM-Specific | Psychopathology | Effect Size ($r$) |
|---|---|---|---|---|---|---|---|---|---|---|
| | | | | | | | Family | N | Suicidality | -.05 |
| | | | | | | | Peers | N | Suicidality | -.28 |
| | | | | | | | Partner | N | Suicidality | -.13 |
| | 28 | Contact | - | NA | .00 | 1.00 | Family | N | Depression | .02 |
| | 28 | Contact | - | NA | .00 | 1.00 | Peers | N | Depression | -.23 |
| | | | | | | | Partner | N | Depression | -.08 |
| | | | | | | | Family | N | Suicidality | .01 |
| | | | | | | | Peers | N | Suicidality | -.39 |
| | | | | | | | Partner | N | Suicidality | -.17 |
| Moody (2013) | 133 | Journal Article | - | 36.75 | 1.00 | .00 | Peers | N | Suicidality | -.17 |
| | | | | | | | Family | N | Suicidality | -.42 |
| Moran (2018) | 347 | Journal Article | - | 21.3 | .17 | .28 | Peers | N | Depression | -.38 |

| Study | N | Source | Structural Stigma | Age (Mean) | % Gender Minority | % Bisexual | Social Support | SGM-Specific | Psychopathology | Effect Size (*r*) |
|---|---|---|---|---|---|---|---|---|---|---|
| | | | | | | | Family | N | Depression | -.45 |
| | | | | | | | General | N | Depression | -.40 |
| Mustanski (2011) | 425 | Journal Article | - | 20.2 | .00 | .25 | Family | N | Depression/ Anxiety | -.33 |
| | | | | | | | Peers | N | Depression/ Anxiety | -.37 |
| Mustanski (2013) | 237 | Journal Article | - | 18.76 | .09 | .29 | Peers | N | Depression | -.29 |
| | | | | | | | Family | N | Depression | -.26 |
| | | | | | | | Family | N | Suicidality | -.02 |
| | | | | | | | Peers | N | Suicidality | .03 |
| Nelson (2024) | 56 | Contact | - | NA | .00 | .00 | Family | N | Depression | -.14 |
| | | | | | | | Peers | N | Depression | -.15 |
| | 32 | Contact | - | NA | .00 | 1.00 | Family | N | Depression | .01 |

| Study | N | Source | Structural Stigma | Age (Mean) | % Gender Minority | % Bisexual | Social Support | SGM-Specific | Psychopathology | Effect Size ($r$) |
|---|---|---|---|---|---|---|---|---|---|---|
| | | | | | | | Peers | N | Depression | .14 |
| | 40 | Contact | - | NA | .00 | .00 | Family | N | Depression | -.17 |
| | | | | | | | Peers | N | Depression | -.40 |
| | 36 | Contact | - | NA | .00 | 1.00 | Family | N | Depression | -.02 |
| | | | | | | | Peers | N | Depression | -.10 |
| Normansell-Mossa (2023) | 54 | Contact | - | 26.97 | .37 | .70 | General | Y | Depression | -.13 |
| | | | | | | | General | Y | Anxiety | -.10 |
| O'Neill (1998) | 97 | Dissertation | GSS | 20.62 | .00 | .36 | General | N | Depression | -.42 |
| Oetjen (2000) | 167 | Journal Article | - | 33 | .00 | .00 | Family | N | Depression | -.49 |
| | | | | | | | Peers | N | Depression | -.55 |
| Ogunbajo (2020) | 30 | Journal Article | - | 29.1 | .00 | .50 | Family | N | Depression | -.26 |

| Study | N | Source | Structural Stigma | Age (Mean) | % Gender Minority | % Bisexual | Social Support | SGM-Specific | Psychopathology | Effect Size (*r*) |
|---|---|---|---|---|---|---|---|---|---|---|
| | | | | | | | Peers | N | Depression | -.04 |
| | | | | | | | Partner | N | Depression | -.15 |
| Olsavsky (2023) | 75 | Journal Article | - | 16.39 | .92 | .00 | Family | N | Depression | -.30 |
| | | | | | | | | N | Anxiety | -.13 |
| | | | | | | | | N | NSSI/Self-Harm | -.06 |
| Onar (2024) | 67 | Journal Article | - | 25.3 | 1.00 | .27 | General | N | Depression | -.43 |
| Otis (1996) | 473 | Journal Article | - | 34.4 | .00 | .00 | Partner | N | Depression | -.25 |
| | | | | | | | Peers | N | Depression | -.07 |
| | | | | | | | General | N | Depression | -.22 |
| | 440 | Journal Article | - | 34.4 | .00 | .00 | Partner | N | Depression | -.37 |

| Study | N | Source | Structural Stigma | Age (Mean) | % Gender Minority | % Bisexual | Social Support | SGM-Specific | Psychopathology | Effect Size ($r$) |
|---|---|---|---|---|---|---|---|---|---|---|
| | | | | | | | Peers | N | Depression | -.09 |
| | | | | | | | General | N | Depression | -.37 |
| Pachankis (2008); Pachankis (2010) | 77 | Dissertation | GSS | 20.19 | .00 | .00 | General | N | Depression | -.35 |
| Pachankis (2018) | 113 | Journal Article | GSS | 20.78 | .00 | .19 | Family | Y | Anxiety | -.09 |
| | | | | | | | Family | Y | Depression | -.15 |
| Pachankis (2024) | 465 | Contact | ESS | 25.1 | .00 | .74 | Family | Y | Depression | -.26 |
| | | | | | | | Family | Y | Anxiety | -.23 |
| | | | | | | | Family | Y | Suicidality | -.14 |
| Pakenham (2001) | 114 | Journal Article | - | 36 | .00 | .00 | General | Y | Depression | -.30 |
| Palmer (2023) | 150 | Journal Article | - | 15.77 | .24 | .83 | Family | Y | Depression | -.22 |

| Study | N | Source | Structural Stigma | Age (Mean) | % Gender Minority | % Bisexual | Social Support | SGM-Specific | Psychopathology | Effect Size (*r*) |
|---|---|---|---|---|---|---|---|---|---|---|
| | | | | | | | Family | Y | Anxiety | -.19 |
| Pantalone (2007); Pantalone (2012) | 157 | Dissertation | GSS | 44.4 | .02 | .00 | General | Y | Depression | -.20 |
| | | | | | | | General | Y | Anxiety | -.24 |
| Pate (2023) | 350 | Journal Article | GSS | NA | .42 | .45 | Family | Y | Suicidality | -.11 |
| | | | | | | | Peers | Y | Suicidality | .02 |
| | | | | | | | General | Y | Suicidality | -.08 |
| Patki (2024) | 30 | Journal Article | - | 30.63 | 1.00 | .00 | Family | N | Depression | -.68 |
| | | | | | | | Partner | N | Depression | -.38 |
| Pereira (2022) | 210 | Journal Article | - | 58.85 | .00 | .00 | General | Y | Depression | -.30 |
| | | | | | | | General | Y | Anxiety | -.29 |

| Study | N | Source | Structural Stigma | Age (Mean) | % Gender Minority | % Bisexual | Social Support | SGM-Specific | Psychopathology | Effect Size (*r*) |
|---|---|---|---|---|---|---|---|---|---|---|
| Perry (2023) | 517 | Journal Article | - | 60 | .00 | .05 | Partner | N | Depression | -.31 |
| | | | | | | | Partner | N | Anxiety | -.26 |
| Ploskonka (2016) | 100 | Dissertation | - | 20.6 | .01 | .62 | Family | N | Suicidality | -.39 |
| | | | | | | | Peers | N | Suicidality | -.40 |
| | | | | | | | School | N | Suicidality | -.32 |
| Pitoňák (2024) | 1778 | Contact | - | 24.2 | .092 | .371 | Family | N | Suicidality | -.41 |
| | | | | | | | Peers | N | Suicidality | -.21 |
| | | | | | | | Partner | N | Suicidality | -.27 |
| | | | | | | | General | N | Suicidality | -.34 |
| Pollit (2017) | 383 | Journal Article | GSS | 18.3 | .00 | 1.00 | Family (Parents) | N | Depression | -.43 |
| | | | | | | | Peers | N | Depression | -.24 |

| Study | N | Source | Structural Stigma | Age (Mean) | % Gender Minority | % Bisexual | Social Support | SGM-Specific | Psychopathology | Effect Size (*r*) |
|---|---|---|---|---|---|---|---|---|---|---|
| Poteat (2011) | 606 | Journal Article | GSS | 14.85 | .00 | .00 | Family (Parents) | N | Suicidality | -.23 |
| Puckett (2019) | 695 | Journal Article | GSS | 25.52 | .79 | .59 | Family | N | Depression | -.37 |
| | | | | | | | Peers | N | Depression | -.15 |
| | | | | | | | Family | N | Anxiety | -.31 |
| | | | | | | | Peers | N | Anxiety | -.08 |
| Rabasco (2021) | 180 | Contact | GSS | 26.01 | .83 | .39 | Peers (SGM) | Y | Suicidality | -.08 |
| | | | | | | | Peers (SGM) | Y | Depression | -.67 |
| Ranjit (2021) | 359 | Journal Article | - | 34 | .13 | .17 | General | N | Depression | .05 |

| Study | N | Source | Structural Stigma | Age (Mean) | % Gender Minority | % Bisexual | Social Support | SGM-Specific | Psychopathology | Effect Size ($r$) |
|---|---|---|---|---|---|---|---|---|---|---|
| Reyes (2015) | 340 | Journal Article | - | NA | .00 | .00 | Family (Parents) | N | Suicidality | -.32 |
| Rimmer (2021) | 217 | Journal Article | GSS | NA | 1.00 | .76 | General | Y | Suicidality | -.47 |
| | | | | | | | Family | N | Suicidality | -.37 |
| | | | | | | | Peers (SGM) | Y | Suicidality | .01 |
| | | | | | | | Peers (Non-SGM) | N | Suicidality | -.27 |
| | | | | | | | Partner | N | Suicidality | -.02 |
| | | | | | | | General | N | Suicidality | -.25 |
| Rimmer (2023) | 217 | Journal Article | - | NA | .54 | .76 | Family | Y | Suicidality | -.37 |

| Study | N | Source | Structural Stigma | Age (Mean) | % Gender Minority | % Bisexual | Social Support | SGM-Specific | Psychopathology | Effect Size (*r*) |
|---|---|---|---|---|---|---|---|---|---|---|
| | | | | | | | Peers (SGM) | Y | Suicidality | .01 |
| | | | | | | | Peers (Non-SGM) | Y | Suicidality | -.27 |
| | | | | | | | Partner | Y | Suicidality | -.02 |
| Rivas-Koehl (2022) | 1078 | Journal Article | GSS | 14.97 | .00 | .78 | General | Y | Suicidality | -.16 |
| | | | | | | | General | Y | Depression | -.22 |
| Robles (2024a) | 94 | Journal Article | GSS | 24.27 | .00 | .38 | Peers | N | Depression | -.32 |
| | | | | | | | Peers | N | Anxiety | -.26 |
| Robles (2024b) | 612 | Contact | - | 31.29 | .00 | .12 | Family | N | Depression | -.39 |
| | | | | | | | Peers | N | Depression | -.46 |
| | | | | | | | Partner | N | Depression | -.46 |

| Study | N | Source | Structural Stigma | Age (Mean) | % Gender Minority | % Bisexual | Social Support | SGM-Specific | Psychopathology | Effect Size (*r*) |
|---|---|---|---|---|---|---|---|---|---|---|
| | | | | | | | Family | N | Anxiety | -.31 |
| | | | | | | | Peers | N | Anxiety | -.33 |
| | | | | | | | Partner | N | Anxiety | -.31 |
| Rogowska (2024) | 509 | Journal Article | - | 22.39 | .34 | .71 | Family | N | Depression | -.25 |
| | | | | | | | Peers | N | Depression | -.27 |
| | | | | | | | Partner | N | Depression | -.18 |
| Rosario (2012) | 156 | Journal Article | GSS | 18.3 | .00 | .34 | Family | N | Depression | -.23 |
| | | | | | | | Family | N | Anxiety | -.07 |
| | | | | | | | Peers | N | Depression | -.35 |
| | | | | | | | Peers | N | Anxiety | -.13 |
| Rotondi (2012) | 186 | Journal Article | - | NA | 1.00 | .00 | General | N | Depression | -.18 |

| Study | N | Source | Structural Stigma | Age (Mean) | % Gender Minority | % Bisexual | Social Support | SGM-Specific | Psychopathology | Effect Size (*r*) |
|---|---|---|---|---|---|---|---|---|---|---|
| Salim (2024) | 400 | Journal Article | - | 25.6 | .02 | 1.00 | General | Y | Suicidality | -.13 |
| Samrock (2021) | 148 | Journal Article | GSS | 26.38 | .95 | .00 | Family | N | Depression | -.24 |
| Sarno (2020) | 1130 | Journal Article | GSS | 21.38 | .05 | .26 | General | Y | Depression | -.26 |
| Sarno (2021) | 213 | Journal Article | GSS | 20.63 | .27 | .73 | Partner | N | Anxiety | -.08 |
| | | | | | | | Partner | N | Depression | -.19 |
| Scandurra (2020a); Scandurra (2020b) | 203 | Journal Article | ESS | 30.7 | 1.00 | .00 | General | Y | Depression | -.14 |
| | | | | | | | General | Y | Anxiety | -.18 |
| | | | | | | | Family | N | Depression | -.26 |
| | | | | | | | Peers | N | Depression | -.21 |

| Study | N | Source | Structural Stigma | Age (Mean) | % Gender Minority | % Bisexual | Social Support | SGM-Specific | Psychopathology | Effect Size ($r$) |
|---|---|---|---|---|---|---|---|---|---|---|
| | | | | | | | Partner | N | Depression | -.12 |
| | | | | | | | Family | N | Anxiety | -.34 |
| | | | | | | | Peers | N | Anxiety | -.29 |
| | | | | | | | Partner | N | Anxiety | -.17 |
| Scheer (2021) | 6305 | Journal Article | - | 33.2 | .00 | .18 | General | N | Suicidality | -.17 |
| Seabra (2023) | 264 | Journal Article | ESS | 28.4 | .08 | .35 | Family | N | Depression | -.27 |
| | | | | | | | Peers | N | Depression | -.30 |
| | | | | | | | Family | N | Anxiety | -.32 |
| | | | | | | | Peers | N | Anxiety | -.22 |
| Sheets (2009) | 210 | Journal Article | - | 20.96 | .00 | 1.00 | Peers | N | Depression | -.34 |
| | | | | | | | Family | N | Depression | -.33 |

| Study | N | Source | Structural Stigma | Age (Mean) | % Gender Minority | % Bisexual | Social Support | SGM-Specific | Psychopathology | Effect Size ($r$) |
|---|---|---|---|---|---|---|---|---|---|---|
| | | | | | | | Peers | Y | Depression | -.11 |
| | | | | | | | Family | Y | Depression | -.10 |
| Shenkman (2022) | 36 | Contact | - | NA | .00 | .00 | General | N | Depression | -.39 |
| Shepherd (2024) | 792 | Contact | - | 23.2 | .15 | .50 | General | Y | Depression | -.25 |
| | | | | | | | School | Y | Depression | -.15 |
| | | | | | | | General | Y | NSSI/Self-Harm | -.16 |
| | | | | | | | School | Y | NSSI/Self-Harm | -.10 |
| Shrader (2024) | 482 | Contact | - | 22 | .30 | .67 | Family | Y | Depression/ Anxiety | .26 |
| Sigurvinsdottir (2024) | 1584 | Contact | ESS | 17.8 | .00 | .00 | Family (Parents) | N | Depression | -.27 |
| | | | | | | | Peers | N | Depression | -.11 |
| | | | | | | | Family (Parents) | N | Anxiety | -.12 |

| Study | N | Source | Structural Stigma | Age (Mean) | % Gender Minority | % Bisexual | Social Support | SGM-Specific | Psychopathology | Effect Size ($r$) |
|---|---|---|---|---|---|---|---|---|---|---|
| | | | | | | | Peers | N | Anxiety | .04 |
| Skidmore (2024) | 339 | Journal Article | GSS | 34.41 | .20 | .48 | Family | N | Depression | -.26 |
| Smith (2017) | 239 | Contact | - | 31.47 | .00 | .50 | Work | N | Depression | -.30 |
| | | | | | | | Work | N | Anxiety | -.33 |
| Smith2020a | 252 | Journal Article | - | 14.66 | .53 | .48 | Family | N | Depression | -.48 |
| Smith2020b | 239 | Journal Article | - | 31.47 | .00 | .50 | Work | N | NSSI/Self-Harm | -.27 |
| Soler (2018) | 350 | Journal Article | GSS | 23 | .00 | .11 | General | N | Depression | -.30 |
| | | | | | | | General | N | Anxiety | -.12 |
| Srikummoon (2021) | 205 | Contact | - | NA | 1.00 | .00 | General | N | Depression | -.10 |
| Srivastava (2021) | 1366 | Contact | - | 33.7 | 1.00 | .00 | General | N | Depression | -.45 |

| Study | N | Source | Structural Stigma | Age (Mean) | % Gender Minority | % Bisexual | Social Support | SGM-Specific | Psychopathology | Effect Size (*r*) |
|---|---|---|---|---|---|---|---|---|---|---|
| | 2182 | Contact | - | 32.7 | .00 | .00 | General | N | Depression | -.82 |
| Sullivan (2021) | 115 | Contact | GSS | 30 | .30 | .24 | Family | N | Depression | -.21 |
| Sullivan (2022) | 84 | Data | - | 25.4 | .35 | .60 | Peers | Y | Anxiety | -.22 |
| | | | | | | | Work | Y | Anxiety | -.27 |
| | | | | | | | Partner | Y | Anxiety | -.01 |
| | | | | | | | Peers | Y | Depression | -.22 |
| | | | | | | | Work | Y | Depression | -.27 |
| | | | | | | | Partner | Y | Depression | -.04 |
| Sutter (2016) | 192 | Journal Article | GSS | 29.5 | .15 | .53 | General | Y | Depression | -.40 |
| | | | | | | | General | Y | Anxiety | -.39 |
| | | | | | | | General | Y | Suicidality | -.24 |
| | | | | | | | Work | Y | Depression | -.16 |
| | | | | | | | Work | Y | Anxiety | -.27 |

| Study | N | Source | Structural Stigma | Age (Mean) | % Gender Minority | % Bisexual | Social Support | SGM-Specific | Psychopathology | Effect Size ($r$) |
|---|---|---|---|---|---|---|---|---|---|---|
| | | | | | | | Work | Y | Suicidality | -.13 |
| Szymanski (2001) | 157 | Journal Article | - | 36.06 | .00 | .18 | General | N | Depression | -.19 |
| | | | | | | | General | Y | Depression | -.19 |
| Szymanski (2008) | 304 | Journal Article | - | 39.92 | .00 | .19 | General | N | Depression/ Anxiety | -.37 |
| Tabaac (2016) | 150 | Journal Article | - | 31.9 | .00 | .61 | Family | N | Suicidality | -.30 |
| | | | | | | | Peers | N | Suicidality | -.11 |
| | | | | | | | Partner | N | Suicidality | -.34 |
| Tabaac (2018) | 78 | Journal Article | - | 29.5 | 1.00 | .72 | General | Y | Anxiety | -.32 |
| | | | | | | | General | Y | Depression | -.48 |
| | | | | | | | Work | Y | Anxiety | -.24 |

| Study | N | Source | Structural Stigma | Age (Mean) | % Gender Minority | % Bisexual | Social Support | SGM-Specific | Psychopathology | Effect Size (*r*) |
|---|---|---|---|---|---|---|---|---|---|---|
| | | | | | | | Work | Y | Depression | -.20 |
| Takeda (2021) | 544 | Journal Article | GSS | 27.84 | .04 | .29 | General | Y | Anxiety | -.02 |
| | | | | | | | General | Y | Depression | .01 |
| Taliaferro (2017) | 2223 | Contact | GSS | NA | .00 | 1.00 | Family (Parents) | N | NSSI/Self-Harm | -.02 |
| | | | | | | | Family (Parents) | N | Suicidality | -.01 |
| | | | | | | | School | N | NSSI/Self-Harm | -.20 |
| | | | | | | | School | N | Suicidality | -.23 |
| | | | | | | | Peers (Non-SGM) | Y | NSSI/Self-Harm | -.08 |
| | | | | | | | Peers (Non-SGM) | Y | Suicidality | -.10 |

| Study | N | Source | Structural Stigma | Age (Mean) | % Gender Minority | % Bisexual | Social Support | SGM-Specific | Psychopathology | Effect Size (*r*) |
|---|---|---|---|---|---|---|---|---|---|---|
| | 655 | Contact | GSS | NA | .00 | .00 | Family (Parents) | N | NSSI/Self-Harm | -.20 |
| | | | | | | | Family (Parents) | N | Suicidality | -.03 |
| | | | | | | | School | N | NSSI/Self-Harm | .01 |
| | | | | | | | Peers (Non-SGM) | Y | NSSI/Self-Harm | -.03 |
| | | | | | | | Peers (Non-SGM) | Y | Suicidality | -.07 |
| | 2082 | Contact | GSS | NA | .00 | 1.00 | Family (Parents) | N | NSSI/Self-Harm | -.04 |
| | | | | | | | Family (Parents) | N | Suicidality | -.01 |
| | | | | | | | School | N | NSSI/Self-Harm | -.05 |

| Study | N | Source | Structural Stigma | Age (Mean) | % Gender Minority | % Bisexual | Social Support | SGM-Specific | Psychopathology | Effect Size (*r*) |
|---|---|---|---|---|---|---|---|---|---|---|
| | | | | | | | School | N | Suicidality | -.05 |
| | | | | | | | Peers (Non-SGM) | Y | NSSI/Self-Harm | -.13 |
| | | | | | | | Peers (Non-SGM) | Y | Suicidality | -.14 |
| Tan (2021) | 570 | Contact | GSS | 21.9 | .02 | .28 | General | Y | Depression | -.30 |
| | | | | | | | General | Y | Suicidality | -.15 |
| Tantirattanakulchai (2022) | 280 | Journal Article | - | 29.7 | 1.00 | .00 | General | N | Depression | -.59 |
| Tebbe (2016) | 335 | Journal Article | - | 25.21 | .98 | .84 | Family | N | Depression | -.37 |
| | | | | | | | Peers | N | Depression | -.43 |
| | | | | | | | Partner | N | Depression | -.31 |

| Study | N | Source | Structural Stigma | Age (Mean) | % Gender Minority | % Bisexual | Social Support | SGM-Specific | Psychopathology | Effect Size (*r*) |
|---|---|---|---|---|---|---|---|---|---|---|
| Terry (2020) | 112 | Dissertation | - | 20.9 | .06 | .84 | General | Y | Depression | -.05 |
| | | | | | | | General | Y | Anxiety | -.08 |
| Testa (2015) | 844 | Journal Article | - | 41.27 | .68 | .00 | General | Y | Depression | -.18 |
| | | | | | | | General | N | Depression | -.43 |
| | | | | | | | General | Y | Anxiety | -.08 |
| Testa (2017) | 814 | Journal Article | - | 32.53 | 1.00 | .00 | General | Y | Suicidality | -.18 |
| Thoma (2025) | 455 | Journal Article | | 15.52 | 0 | .42 | Family | Y | Depression | -.33 |
| Toomey (2025) | 319 | Journal Article | | 18.64 | .771 | .734 | Family | Y | Depression | -.16 |
| | | | | | | | Family | Y | Anxiety | -.08 |

| Study | N | Source | Structural Stigma | Age (Mean) | % Gender Minority | % Bisexual | Social Support | SGM-Specific | Psychopathology | Effect Size (*r*) |
|---|---|---|---|---|---|---|---|---|---|---|
| Tornello (2016) | 79 | Journal Article | GSS | 61.05 | .00 | .00 | General | N | Depression/ Anxiety | -.38 |
| Travers (2020) | 123 | Contact | ESS | 22.96 | .00 | .00 | Partner | N | Depression | .00 |
| | | | | | | | Peers | N | Depression | -.22 |
| | | | | | | | Family | N | Depression | -.44 |
| | | | | | | | Partner | N | Anxiety | .01 |
| | | | | | | | Peers | N | Anxiety | -.16 |
| | | | | | | | Family | N | Anxiety | -.44 |
| Trujillo (2017) | 78 | Contact | GSS | 29.6 | .71 | .56 | Family | N | Anxiety | -.13 |
| | | | | | | | Family | N | Depression | -.24 |
| Truong (2010) | 223 | Dissertation | - | 28 | .00 | .07 | General | Y | Depression | -.05 |
| Tsai (2021) | 1000 | Contact | - | 24.6 | .00 | .43 | General | Y | Anxiety | -.23 |
| | | | | | | | General | Y | Depression | -.23 |

| Study | N | Source | Structural Stigma | Age (Mean) | % Gender Minority | % Bisexual | Social Support | SGM-Specific | Psychopathology | Effect Size (*r*) |
|---|---|---|---|---|---|---|---|---|---|---|
| Tse (2017) | 239 | Journal Article | - | 38.9 | .00 | .00 | General | N | Depression | -.49 |
| Tse (2018) | 389 | Journal Article | - | 33.2 | .00 | .51 | General | N | Depression | -.48 |
| Turpin (2020) | 1063 | Journal Article | GSS | 40 | .00 | .00 | General | N | Depression | -.35 |
| Tüzün (2022) | 49 | Journal Article | - | 20.53 | 1.00 | .00 | Family | N | Depression | -.62 |
| | | | | | | | Peers | N | Depression | -.38 |
| | | | | | | | Partner | N | Depression | -.15 |
| | | | | | | | Family | N | Anxiety | -.64 |
| | | | | | | | Peers | N | Anxiety | -.52 |
| | | | | | | | Partner | N | Anxiety | -.30 |

| Study | N | Source | Structural Stigma | Age (Mean) | % Gender Minority | % Bisexual | Social Support | SGM-Specific | Psychopathology | Effect Size ($r$) |
|---|---|---|---|---|---|---|---|---|---|---|
| Ueno (2009) | 84 | Journal Article | GSS | 18.3 | .00 | .00 | General | Y | Depression/ Anxiety | -.26 |
| Vale (2021) | 129 | Journal Article | - | 28.41 | .06 | .29 | Family | N | Depression | -.43 |
| | | | | | | | Peers | N | Depression | -.43 |
| | 104 | Journal Article | - | 56.64 | .05 | .10 | Family | N | Depression | -.44 |
| | | | | | | | Peers | N | Depression | -.55 |
| Valente (2020) | 330 | Journal Article | - | 34.4 | .81 | .64 | Family | N | Depression/ Anxiety | -.30 |
| van Stolk-Cooke (2024) | 235 | Journal Article | - | 23.93 | .00 | .63 | Partner | N | Anxiety | -.21 |
| | | | | | | | Family | N | Anxiety | -.17 |
| | | | | | | | Peers | N | Anxiety | -.17 |

| Study | N | Source | Structural Stigma | Age (Mean) | % Gender Minority | % Bisexual | Social Support | SGM-Specific | Psychopathology | Effect Size ($r$) |
|---|---|---|---|---|---|---|---|---|---|---|
| Van Beusekom (2018) | 395 | Dissertation | ESS | 31.42 | .00 | .07 | General | Y | Depression/ Anxiety | -.20 |
| | 329 | Dissertation | ESS | 31.42 | .00 | .27 | General | Y | Depression/ Anxiety | -.17 |
| VanDam (2014) | 265 | Journal Article | - | 43 | .00 | .00 | General | N | Depression | -.46 |
| Veale (2017) | 600 | Journal Article | - | NA | .40 | .00 | General | N | NSSI/Self-Harm | -.52 |
| Velasco (2024) | 110 | Journal Article | | 25.2 | .045 | 1 | General | N | Depression | -.50 |
| | 110 | Journal Article | | 25.2 | .045 | 1 | General | N | Anxiety | -.19 |
| Velez (2023) | 434 | Journal Article | - | 35.61 | .08 | .56 | General | Y | Depression | -.39 |

| Study | N | Source | Structural Stigma | Age (Mean) | % Gender Minority | % Bisexual | Social Support | SGM-Specific | Psychopathology | Effect Size (*r*) |
|---|---|---|---|---|---|---|---|---|---|---|
| | | | | | | | Work | Y | Depression | -.30 |
| | | | | | | | Family | N | Depression | -.31 |
| | | | | | | | Peers | N | Depression | -.27 |
| | | | | | | | Partner | N | Depression | -.68 |
| | | | | | | | General | Y | Anxiety | -.39 |
| | | | | | | | Work | Y | Anxiety | -.30 |
| | | | | | | | Family | N | Anxiety | -.24 |
| | | | | | | | Peers | N | Anxiety | -.13 |
| | | | | | | | Partner | N | Anxiety | -.67 |
| Vincke (2004) | 111 | Journal Article | - | NA | .00 | .13 | General | N | Depression | -.43 |
| Vogel (2021) | 302 | Journal Article | GSS | 21.9 | 1.00 | .66 | General | N | Depression | -.28 |

| Study | N | Source | Structural Stigma | Age (Mean) | % Gender Minority | % Bisexual | Social Support | SGM-Specific | Psychopathology | Effect Size (*r*) |
|---|---|---|---|---|---|---|---|---|---|---|
| Vogel (2024) | 5995 | Journal Article | - | 28.7 | .105 | .764 | General | N | Depression | -.287 |
| Wade (2022) | 603 | Contact | GSS | 24.46 | .00 | .16 | General | N | Depression | -.44 |
| Walsh (2020) | 500 | Dissertation | GSS | 35.4 | .05 | .31 | General | Y | Depression/ Anxiety | .34 |
| | | | | | | | School/Work | Y | Depression/ Anxiety | -.33 |
| Wang (2021) | 581 | Journal Article | - | 26.96 | .00 | .45 | General | Y | Depression | -.13 |
| Watts (2023) | 211 | Journal Article | - | 18.1 | .37 | .767 | Family | Y | Depression | -.24 |
| | | | | | | | Peers | Y | Depression | -.18 |
| | | | | | | | Family | Y | Anxiety | -.17 |
| | | | | | | | Peers | Y | Anxiety | -.11 |

| Study | N | Source | Structural Stigma | Age (Mean) | % Gender Minority | % Bisexual | Social Support | SGM-Specific | Psychopathology | Effect Size ($r$) |
|---|---|---|---|---|---|---|---|---|---|---|
| Wei (2024) | 693 | Journal Article | - | 27.1 | 0 | .268 | General | N | Depression | -.37 |
| Williams (2020) | 315 | Contact | GSS | 23.4 | .16 | .52 | General | N | Depression | -.26 |
| | | | | | | | General | N | Anxiety | -.18 |
| Woodford (2015) | 326 | Journal Article | - | 23 | .00 | .89 | General | Y | Depression | -.21 |
| | | | | | | | General | Y | Anxiety | -.17 |
| Woodward (2012) | 136 | Journal Article | GSS | 44.4 | .00 | .00 | General | N | Depression | -.22 |
| | | | | | | | General | N | Anxiety | -.22 |
| Wongsom (2024) | 205 | Journal Article | | 19.47 | .205 | .703 | General | N | Depression | -.33 |
| | | | | | | | | | Anxiety | -.16 |

| Study | N | Source | Structural Stigma | Age (Mean) | % Gender Minority | % Bisexual | Social Support | SGM-Specific | Psychopathology | Effect Size (*r*) |
|---|---|---|---|---|---|---|---|---|---|---|
| Wright (2022) | 466 | Journal Article | - | 15.76 | .02 | .00 | School/Work | Y | Depression | -.23 |
| | | | | | | | School/Work | Y | Anxiety | -.20 |
| Yang (2016) | 200 | Journal Article | - | 26.64 | 1.00 | .20 | General | N | Anxiety | -.31 |
| Zea (1999) | 106 | Journal Article | - | 33 | .00 | .00 | General | N | Depression | -.55 |
| Zhang (2021) | 361 | Journal Article | - | 25.13 | 1.00 | .00 | General | Y | Depression | -.32 |
| Zhou (2024) | 1014 | Journal Article | - | 26.2 | 0 | 0 | General | N | Depression | -.189 |

**S2b. References of studies included in the meta-analysis**

Abreu, R. L., Tyler Lefevor, G., Barrita, A. M., Gonzalez, K. A., & Watson, R. J. (2023). Intersectional microaggressions, depressive symptoms, and the role of LGBTQ-specific parental support in a sample of Latinx sexual and gender minority youth. *Journal of Adolescence, 95*(3), 584-595. https://doi.org/10.1002/jad.12139

Algarin, A. B., Smith, L. R., Pines, H. A., Zapien-Vasquez, M. F., Padilla-Garcia, R., Navarro-Alvarez, S., & Pitpitan, E. V. (2024). The gender minority stress and resilience measure: Psychometric validity for use in Spanish. *Transgender Health, 9*(1), 24-33. https://doi.org/10.1089/trgh.2021.0190

Al-Khouja, M., Weinstein, N., & Legate, N. (2021). Long-term mental health correlates of social supportive relationships in a lesbian, gay, and bisexual sample. *Psychology & Sexuality*, *12*(3), 180–192. https://doi.org/10.1080/19419899.2019.1687580

Allen, V. C., Myers, H. F., & Williams, J. K. (2014). Depression among black bisexual men with early and later life adversities. *Cultural Diversity and Ethnic Minority Psychology*, *20*(1), 128–137. https://doi.org/10.1037/a0034128

Alpert, C. (2024). The Impact of Religious Fundamentalism on Sexual Identity Development for Sexual Minority Individuals (Doctoral dissertation, Alliant International University). 31297622.

Aristegui, I., Radusky, P. D., Zalazar, V., Cardozo, N., Fabian, S., Duarte, M., Frola, C., Cahn, P., & Sued, O. (2021). Correlates of depressive symptoms in transgender women initiating HIV treatment in Argentina. *Journal of Gay & Lesbian Mental Health*, *25*(2), 208–225. https://doi.org/10.1080/19359705.2020.1868370

Arnett, J. E., Frantell, K. A., Miles, J. R., & Fry, K. M. (2019). Anti-bisexual

discrimination as insidious trauma and impacts on mental and physical health. *Psychology of Sexual Orientation and Gender Diversity*, *6*(4), 475–485. https://doi.org/10.1037/sgd0000344

Ayala, J., & Coleman, H. (2000). Predictors of depression among lesbian women. *Journal of Lesbian Studies*, *4*(3), 71–86. https://doi.org/10.1300/J155v04n03_04

Bartoshuk, M. R. (2009). *Minority coping: The role of interpersonal resiliency factors in gay men's experience of minority stress and depression.* [PhD Thesis, University of Toronto].

Bazargan, M., & Galvan, F. (2012). Perceived discrimination and depression among low-income Latina male-to-female transgender women. *BMC Public Health*, *12*(1), 663. https://doi.org/10.1186/1471-2458-12-663

Beggiato, E. (2024). The Impact of Social Support on Mental Health Outcomes Among Transgender and Gender Expansive Intimate Partner Violence Survivors (Doctoral dissertation, Alliant International University). 31485668.

Benibgui, M. (2010). *Mental health challenges and resilience in lesbian, gay, and bisexual young adults: Biological and psychological internalization of minority stress and victimization* [PhD Thesis, Concordia University]. https://spectrum.library.concordia.ca/id/eprint/979282/

Berg, R. C., Carter, D., & Ross, M. W. (2017). A mixed-method study on correlates of HIV-related stigma among gay and bisexual men in the southern United States. *Journal of the Association of Nurses in AIDS Care*, *28*(4), 532–544. https://doi.org/10.1016/j.jana.2017.02.004

Bergfeld, J. R., & Chiu, E. Y. (2017). Mediators in the relationship between minority stress and depression among young same-sex attracted women. *Professional*

*Psychology: Research and Practice*, *48*(5), 294–300. https://doi.org/10.1037/pro0000155

Berlin, S. (2007). *Psychological adjustment to aging among gay men over age fifty* [PhD Thesis, Michigan State University]. https://doi.org/10.25335/M5XS5JW7X

Bittner, K. E. (2016). *Trans*forming understanding of sexual orientation and gender variant minorities: Testing the minority stress model with a diverse sample* [PhD Thesis, Iowa State University]. https://doi.org/10.31274/etd-180810-5506

Bitran, A. M., Sritharan, A., Trivedi, E., Helgren, F., Buchanan, S. N., Durham, K., ... & Pagliaccio, D. (2024). The effects of family support and smartphone-derived homestay on daily mood and depression among sexual and gender minority adolescents. *Journal of Psychopathology and Clinical Science, 133*(5), 358. https://doi.org/10.1037/abn0000917

Bolwell, A. (2021). *Discrimination, mental health, and stress-related growth of sexual minorities* [PhD Thesis, University of La Verne]. https://search.proquest.com/openview/abe2a6b0b9a050f42bcda5bd63953fcb/1?pq-origsite=gscholar&cbl=18750&diss=y

Boyd, D. T., Jones, K. V., Hawthorne, D. J., Quinn, C. R., Mueller-Williams, A. C., Ramos, S. R., ... & Wilton, L. (2024). Examining developmental assets of young Black sexual gender minority males in preventing suicidal behaviors. Journal of *Psychiatric Research, 171*, 256-262. https://doi.org/10.1016/j.jpsychires.2024.01.035

Boyd, D. T., Jones, K. V., Quinn, C. R., Hill, M., Nelson, L. E., Beauchamp, G., ... & Wilton, L. (2024). Ethnic identity and social support as mediators between childhood sexual abuse and depression among black men who have sex with men.

*Child Abuse & Neglect, 157,* 107064. https://doi.org/10.1016/j.chiabu.2024.107064

Boyd, D. T., Quinn, C. R., Jones, K. V., Waller, B., Coker, E. J., Duprey, E. B., ... & McCoy, H. (2025). Building stronger bonds: The impact of family support and communication on suicidal behaviors among Black men who have sex with men. Suicide and Life? *Threatening Behavior, 55*(1), e13072. https://doi.org/10.1016/10.1111/sltb.13072

Boyd, D. T., Ramos, S. R., Quinn, C. R., Jones, K. V., Wilton, L., & Nelson, L. E. (2021). Family support and sociocultural factors on depression among black and Latinx sexual minority men. *International Journal of Environmental Research and Public Health*, *18*(13), 6759. https://doi.org/10.3390/ijerph18136759

Brady, J. P. (2023). *The Effect of Patient Navigation for PrEP-related Health Care on Mental Health among Latinx Sexual Minority Men: Mediation through Social Support*. [PhD Thesis, University of California, San Diego.] 30530875

Brandon-Friedman, R. A., & Kim, H.-W. (2016). Using social support levels to predict sexual identity development among college students who identify as a sexual minority. *Journal of Gay & Lesbian Social Services*, *28*(4), 292–316. https://doi.org/10.1080/10538720.2016.1221784

Budge, S. L., Adelson, J. L., & Howard, K. A. S. (2013). Anxiety and depression in transgender individuals: The roles of transition status, loss, social support, and coping. *Journal of Consulting and Clinical Psychology*, *81*(3), 545–557. https://doi.org/10.1037/a0031774

Budge, S. L., Rossman, H. K., & Howard, K. A. S. (2014). Coping and psychological distress among genderqueer individuals: The moderating effect of social support. *Journal of LGBT Issues in Counseling*, *8*(1), 95–117.

https://doi.org/10.1080/15538605.2014.853641

Burish, E., Wilcox, M. M., Pollard, E. M., & Sims, K. N. (2023). Differentiating protective factors for transgender individuals who experience suicidality: The role of optimism as a mediator. *Clinical Psychology & Psychotherapy, 30*(3), 702-713. https://doi.org/10.1002/cpp.2833

Cain, D. N., Mirzayi, C., Rendina, H. J., Ventuneac, A., Grov, C., & Parsons, J. T. (2017). Mediating effects of social support and internalized homonegativity on the association between population density and mental health among gay and bisexual men. *LGBT Health*, *4*(5), 352–359. https://doi.org/10.1089/lgbt.2017.0002

Carter, L. W., Mollen, D., & Smith, N. G. (2014). Locus of control, minority stress, and psychological distress among lesbian, gay, and bisexual individuals. *Journal of Counseling Psychology*, *61*(1), 169–175. https://doi.org/10.1037/a0034593

Cassidy, K. (2020). *Well-being in trans and gender diverse individuals: An investigation of chosen family support* [PhD Thesis, The University of Nebraska-Lincoln]. https://search.proquest.com/openview/67ee4b28ddc902737335d258a67fbc97/1

Cerezo, A., Rivera, D. B., Sanchez, D., Torres, L., Carlos Chavez, F. L., & Drabble, L. A. (2024). Examining COVID-19 pandemic-related economic and household stress and its association with mental health, alcohol, and substance use in a national sample of Latinx sexual minority and heterosexual adults. *Cultural Diversity & Ethnic Minority Psychology, 30*(2), 385. https://doi.org/10.1037/cdp0000583

Chakrapani, V., Vijin, P. P., Logie, C. H., Newman, P. A., Shunmugam, M., Sivasubramanian, M., & Samuel, M. (2017). Understanding how sexual and gender minority stigmas influence depression among trans women and men who have sex

with men in India. *LGBT Health*, *4*(3), 217–226. https://doi.org/10.1089/lgbt.2016.0082

Chang, C. J., Fischer, I. C., Depp, C. A., Norman, S. B., Livingston, N. A., & Pietrzak, R. H. (2023). A disproportionate burden: Prevalence of trauma and mental health difficulties among sexual minority versus heterosexual U.S. military veterans. *Journal of Psychiatric Research*, *161*, 477–482. https://doi.org/10.1016/j.jpsychires.2023.03.042

Chang, C. J., Kellerman, J. K., Fehling, K. B., Feinstein, B. A., & Selby, E. A. (2021). The roles of discrimination and social support in the associations between outness and mental health outcomes among sexual minorities. *American Journal of Orthopsychiatry*, *91*(5), 607–616. https://doi.org/10.1037/ort0000562

Chelliah, P., Lau, M., & Kuper, L. E. (2024). Changes in gender dysphoria, interpersonal minority stress, and mental health among transgender youth after one year of hormone therapy. *Journal of Adolescent Health, 74*(6), 1106-1111. https://doi.org/10.1016/j.jadohealth.2023.12.024

Chen, J.-K., & Hung, F. N. (2021). Sexual orientation victimization and depression among lesbian, gay and bisexual youths in Hong Kong: The mediating role of social support. *Journal of Aggression, Maltreatment & Trauma*, *30*(5), 679–693. https://doi.org/10.1080/10926771.2020.1821853

Cheung, D. H., Hall, C. D. X., Okantey, B., Meng, Z., Sabuncu, C., Lane, B., Millender, E., Queiroz, A., Kim, J. H., Okada, L., Gillespie, A., Simoncini, G., Barile, J. "J." P., Ma, G. X., & Wong, F. Y. (2025a). Mediating roles of social support in lives of men who have sex with men living with HIV. *Health Psychology, 44*(3), 234–246. https://doi.org/10.1037/hea0001490

Cheung, D. H., Waratworawan, W., Kongjareon, Y., Jonas, K. J., Lim, S. H., Reeves, A. N., & Guadamuz, T. E. (2025b). A Syndemic Clustering of Adversities on Suicide Risk among YMSM Living with HIV in Bangkok: A Causal Latent Class Analysis. *AIDS and Behavior, 29*(2), 420–434. https://doi.org/10.1007/s10461-024-04516-7

Chinazzo, Í. R., Fontanari, A. M. V., Costa, A. B., & Lobato, M. I. R. (2023). Factors associated with suicidal ideation and suicide attempt in Brazilian transgender youth. *International Journal of Environmental Research and Public Health, 20*(4), 3215. https://doi.org/10.3390/ijerph20043215

Christie, C. (2021). What is hidden can still hurt: Concealable stigma, psychological well-being, and social support among LGB college students. *Sexuality Research and Social Policy*, *18*(3), 693–701. https://doi.org/10.1007/s13178-020-00492-4

Clark, T. C., Lucassen, M. F., Bullen, P., Denny, S. J., Fleming, T. M., Robinson, E. M., & Rossen, F. V. (2014). The health and well-being of transgender high school students: Results from the New Zealand adolescent health survey (Youth'12). *Journal of Adolescent Health*, *55*(1), 93–99. https://doi.org/10.1016/j.jadohealth.2013.11.008

Craney, R. S., Watson, L. B., Brownfield, J., & Flores, M. J. (2018). Bisexual women's discriminatory experiences and psychological distress: Exploring the roles of coping and LGBTQ community connectedness. *Psychology of Sexual Orientation and Gender Diversity*, *5*(3), 324–337. https://doi.org/10.1037/sgd0000276

Dai, Z., Fu, J., Qu, Y., Wu, Y., Si, M., Chen, X., ... & Su, X. (2023). Depressive symptoms, perceived social support, and anticipated HIV stigma among HIV?negative/unknown men who have sex with men in China during the COVID?19 pandemic: A multicenter online cross-sectional study. *Brain and Behavior, 13*(4),

e2946. https://doi.org/10.1002/brb3.2946

Das, H. K., & Govindappa, L. (2023). Anxiety, depression and social support of LGBTIQ during COVID-19 in Kerala, India. *International Journal of Social Psychiatry, 69*(8), 1971-1978. https://doi.org/10.1177/00207640231183913

Dejanipont, B., Wang, C., Jenkins, S., Ta, A., Prybutok, V., & Vosvick, M. (2024). Stressor appraisals and moderating role of forgiveness, social support, and resilience as adaptive coping in stress and depression among older sexual minorities. *Sexuality Research and Social Policy, 21*(4), 1247-1258. https://doi.org/10.1007/s13178-023-00831-1

del Pino, H.E., Harawa, N.T., Shoptaw, S.J. et al. Drug use, family support, and depressive symptoms among latinx sexual minority men: A longitudinal analysis. *AIDS Behavior 27,* 3844–3851 (2023). https://doi.org/10.1007/s10461-023-04098-w

DeLaney, E. N., Williams, C. D., Jones, S. C., Hood, K. B., Cage, J., Coston, B. E., ... & Dick, D. M. (2024). Sexual Victimization and Mental Health Among LGBQ+ College Students: Examining Social Support and Trauma-Related Drinking as Mediators. *International Journal of Mental Health and Addiction, 22*(4), 2456-2471. https://doi.org/10.1007/s11469-022-00997-6

DeLaney, E. N., Williams, C. D., Mosley, D. V., Hawn, S. E., & Dick, D. M. (2022). The associations between sexual victimization and health outcomes among LGBQA college students: Examining the moderating role of social support. *Journal of Interpersonal Violence*, *37*(11–12), NP10393–NP10417. https://doi.org/10.1177/0886260520978179

Dellucci, T. V., & Starks, T. J. (2025). Relationship quality and minority stressors

predict mental health symptoms among partnered adolescent sexual minority males. *Couple and Family Psychology: Research and Practice, 14*(1), 80–91. https://doi.org/10.1037/cfp0000264.

Detwiler, B. P. (2015). *Minority stress in the sexual minority older adult population: Exploring the relationships among discrimination, mental health, and quality of life*. [PhD Thesis, Lehigh University]. https://search.proquest.com/openview/6acb269137b6664de935ae3f1f4e4d05/1?pq-origsite=gscholar&cbl=18750

Dickenson, J. A., & Huebner, D. M. (2016). The relationship between sexual activity and depressive symptoms in lesbian, gay, and bisexual youth: Effects of gender and family support. *Archives of Sexual Behavior*, *45*(3), 671–681. https://doi.org/10.1007/s10508-015-0571-8

Ding, C., Chen, X., Wang, W., Yu, B., Yang, H., Li, X., Deng, S., Yan, H., & Li, S. (2020). Sexual minority stigma, sexual orientation concealment, social support and depressive symptoms among men who have sex with men in China: A moderated mediation modeling analysis. *AIDS and Behavior*, *24*(1), 8–17. https://doi.org/10.1007/s10461-019-02713-3

Do, Q. A., Knopp, K., & Scott, S. B. (2022). Intimate partner violence in female same-gender couples: An investigation of actor–partner correlates within the past year. *Psychological Trauma: Theory, Research, Practice, and Policy*, *14*(5), 759–768. https://doi.org/10.1037/tra0001041

Dolezal, M. L., Decker, M., Higgins, M., & Littleton, H. (2023). Dolezal, M. L., Decker, M., Higgins, M., & Littleton, H. (2025). Evaluation of the gender minority stress model in transgender and gender diverse college students. *Psychology of Sexual*

*Orientation and Gender Diversity, 12*(2), 348–358. https://doi.org/10.1037/sgd0000669

Donnelly, R., Robinson, B. A., & Umberson, D. (2019). Can spouses buffer the impact of discrimination on depressive symptoms? An examination of same-sex and different-sex marriages. *Society and Mental Health*, *9*(2), 192–210. https://doi.org/10.1177/2156869318800157

Dowshen, N., Binns, H. J., & Garofalo, R. (2009). Experiences of HIV-related stigma among young men who have sex with men. *AIDS Patient Care and STDs*, *23*(5), 371–376. https://doi.org/10.1089/apc.2008.0256

Drydakis, N. (2022). Social rejection, family acceptance, economic recession, and physical and mental health of sexual minorities. *Sexuality Research and Social Policy*, *19*(3), 1318–1340. https://doi.org/10.1007/s13178-021-00640-4

Du, M., Zhao, J., Zhang, J., Lau, J. T. F., Mo, P. K. H., & Li, J. (2018). Depression and social support mediate the effect of HIV self-stigma on condom use intentions among Chinese HIV-infected men who have sex with men. *AIDS Care*, *30*(9), 1197–1206. https://doi.org/10.1080/09540121.2018.1487916

Dyar, C., Feinstein, B. A., & London, B. (2014). Dimensions of sexual identity and minority stress among bisexual women: The role of partner gender. *Psychology of Sexual Orientation and Gender Diversity*, *1*(4), 441–451. https://doi.org/10.1037/sgd0000063

Dyar, C., Feinstein, B. A., Sarno, E. L., Pirog, S., Newcomb, M. E., & Whitton, S. W. (2021). Prospective associations between bi+ minority stressors and internalizing symptoms: The mediating roles of general and group-specific processes. *Journal of Consulting and Clinical Psychology*, *89*(10), 845–855.

https://doi.org/10.1037/ccp0000689

Dyar, C., Sarno, E. L., Newcomb, M. E., & Whitton, S. W. (2020). Longitudinal associations between minority stress, internalizing symptoms, and substance use among sexual and gender minority individuals assigned female at birth. *Journal of Consulting and Clinical Psychology*, *88*(5), 389–401. https://doi.org/10.1037/ccp0000487

Eadeh, H. M., Breaux, R., Boyd-Rogers, C., Priest, J. B., & Nikolas, M. A. (2023). Self-regulation in Gender and Sexual Orientation Diverse Adults: Exploring Patterns of Risk and Resilience Using a Person-centered Approach. *Journal of LGBTQ Issues in Counseling*, *17*(1), 22–39. https://doi.org/10.1080/26924951.2022.2093310

Earle, H. A. (1999). *The relationship of internalized homophobia, level of outness, perceived social support, and self-esteem to depression in lesbians*. [PhD Thesis, The University of Wisconsin-Madison]. https://search.proquest.com/openview/9b4bf2c3457d6a40acb957155c014c84/1

Edwards, L. L., Torres Bernal, A., Hanley, S. M., & Martin, S. (2020). Resilience factors and suicide risk for a sample of transgender clients. *Family Process*, *59*(3), 1209–1224. https://doi.org/10.1111/famp.12479

Eng Hui, E., & Berezina, E. B. (2025). Burden of the fruity: family support and suicide ideation as mediators between discrimination and suicide behavior in LGBTQ+ Malaysians. *Journal of Homosexuality, 72*(5), 868-889. https://doi.org/10.1080/00918369.2024.2354409

Feinstein, B. A., Latack, J. A., Bhatia, V., Davila, J., & Eaton, N. R. (2016). Romantic relationship involvement as a minority stress buffer in gay/lesbian versus bisexual individuals. *Journal of Gay & Lesbian Mental Health*, *20*(3), 237–257.

https://doi.org/10.1080/19359705.2016.1147401

Feinstein, B. A., Wadsworth, L. P., Davila, J., & Goldfried, M. R. (2014). Do parental acceptance and family support moderate associations between dimensions of minority stress and depressive symptoms among lesbians and gay men? *Professional Psychology: Research and Practice*, *45*(4), 239–246. https://doi.org/10.1037/a0035393

Fiani, C. N. (2018). *Beyond the binary: Gender identity and mental health among transgender and gender non-conforming adults*. [PhD Thesis, The City University of New York]. https://academicworks.cuny.edu/gc_etds/2815/

Fingerhut, A. W. (2018). The role of social support and gay identity in the stress processes of a sample of Caucasian gay men. *Psychology of Sexual Orientation and Gender Diversity*, *5*(3), 294. https://doi.org/10.1037/sgd0000271

Fischer, S. N. (2011). *School-based supports for LGBT and other minority youth: Understanding the roles of teachers, staff, gay-straight alliances, and anti-harassment policies*. [PhD Thesis, New York University]. https://search.proquest.com/openview/1ff0e424a8667a97a1d6c73cee5881ee/1?pq-origsite=gscholar&cbl=18750

Frank, J. A. (2016). *Factors promoting risk and resilience for suicidal ideation among transgender youth*. [PhD Thesis, New York University]. https://search.proquest.com/openview/8253a65d0259a78fb751b3c50022bb84/1

Galarneau, E., & Andersen, J. P. (2025). Bisexual+ mental health disparities: The protective role of social support. *Psychology of Sexual Orientation and Gender Diversity*. Advanced online publication. https://doi.org/10.1037/sgd0000787

Gato, J., Leal, D., & Seabra, D. (2020). When home is not a safe haven: Effects of the

COVID-19 pandemic on sexual and gender minorities in Portugal. *PSICOLOGIA*, *34*(2), 89–100. https://doi.org/10.17575/psicologia.v34i2.1667

Gillig, T. K., Macary, J., & Price, R. (2022). Virtual camp: LGBTQ youths' collective coping during the COVID-19 pandemic. *International Journal of Communication*, *16*, 25. 1932-8036/20220005

Gilmore, T. C. (1996). *Stress, coping and adjustment among gay, lesbian, and bisexual youth*. [Doctoral dissertation, George Mason University]. https://search.proquest.com/openview/3e3b46e4d6e6c00c7e82bbc1eed8d421/1

Glenwright, B. J. (2023). *Mental Health, Coping, Resilience, and Community in Plurisexual Emerging Adults* [Doctoral dissertation, University of Toronto (Canada)]. http://hdl.handle.net/1807/128226

Glynn, T. R., Gamarel, K. E., Kahler, C. W., Iwamoto, M., Operario, D., & Nemoto, T. (2016). The role of gender affirmation in psychological well-being among transgender women. *Psychology of Sexual Orientation and Gender Diversity*, *3*(3), 336–344. https://doi.org/10.1037/sgd0000171

Golembiewski, Lee A.. "The Effect of Minority Stress on Sexual Minority College Students' Mental Health: The Role of General Social Support and Sexuality-Specific Social Support" (2023). [Doctoral dissertation Old Dominion University]. https://doi.org/10.25777/y975-4658

Gray, J., & Hedge, B. (1999). Psychological distress and coping in the partners of gay men with HIV-related disease. *British Journal of Health Psychology*, *4*(2), 117–126. https://doi.org/10.1348/135910799168515

Grocott, L. R., Schlechter, T. E., Wilder, S. M., O'Hair, C. M., Gidycz, C. A., & Shorey, R. C. (2023). Social support as a buffer of the association between sexual assault and

trauma symptoms among transgender and gender diverse individuals. *Journal of Interpersonal Violence, 38*(1-2), 1738-1761. https://doi.org/10.1177/08862605221092069

Greenblatt, C. (2018). *A mixed methods study of minority stress and social support among transgender and genderqueer adults*. [Doctoral Thesis, Adelphi University]. The Institute of Advanced Psychological Studies. https://search.proquest.com/openview/9b19000f26e98b689d628a697b09a39a/1

Grigoriou, J. A. (2014). Minority stress factors for same-sex attracted Mormon adults. *Psychology of Sexual Orientation and Gender Diversity*, *1*(4), 471–479. https://doi.org/10.1037/sgd0000078

Grossman, A. H., Park, J. Y., Frank, J. A., & Russell, S. T. (2021). Parental responses to transgender and gender nonconforming youth: Associations with parent support, parental abuse, and youths' psychological adjustment. *Journal of Homosexuality*, *68*(8), 1260–1277. https://doi.org/10.1080/00918369.2019.1696103

Gutiérrez, B. (2022). *Moderated Mediation of Risk and Protective Factors on Depression in LGBQ Young Adults* [Doctoral Thesis, Alliant International University]. https://search.proquest.com/openview/417c0b661f2dd4a406f9fc6ea3a02b3c/1

Guo, G., Hu, C., Liu, X., Zhang, Q., Huang, X., Chen, Z., ... Li, X. (2025). Latent Profiles of Perceived Social Support and Their Impact on Suicidal Behavior in Chinese Gender Minorities: A Mediated Model. *Issues in Mental Health Nursing*, *46*(5), 462–473. https://doi.org/10.1080/01612840.2025.2468452

Guzman-Parra, J., Sánchez-Álvarez, N., Guzik, J., Bergero-Miguel, T., de Diego-Otero, Y., & Pérez-Costillas, L. (2023). The impact of stressful life events on suicidal

ideation in gender dysphoria: A moderator effect of perceived social support. *Archives of Sexual Behavior, 52*(5), 2205-2213. https://doi.org/10.1007/s10508-023-02594-7

Hammack, P. L., Pletta, D. R., Hughes, S. D., Atwood, J. M., Cohen, E. M., & Clark, R. C. (2024). Community support for sexual and gender diversity, minority stress, and mental health: A mixed-methods study of adolescents with minoritized sexual and gender identities. *Psychology of Sexual Orientation and Gender Diversity, 11*(2), 250. https://doi.org/10.1037/sgd0000591

Handelman, M. S. (2016). *The role of minority stress in social anxiety and depression among lesbian and gay adults*. [PhD Thesis, Alliant International University]. https://search.proquest.com/openview/c71bd0773988d875ed8ccb6d7e5b9f2d/1?pq-origsite=gscholar&cbl=18750&casa_token=dJvi4C0PgLEAAAAA:On3kd4dg-zUaWbeA-AHF3DifDdY-iTGs2ilaAOoge34JMVq9lE_e6C0hFMc1Q0LDD_dkLTXk1YYN

Hatchel, T., Merrin, G. J., & Espelage, A. D. (2019). Peer victimization and suicidality among LGBTQ youth: The roles of school belonging, self-compassion, and parental support. *Journal of LGBT Youth*, *16*(2), 134–156. https://doi.org/10.1080/19361653.2018.1543036

Heiden-Rootes, K., Hartwell, E., & Nedela, M. (2021). Comparing the partnering, minority stress, and depression for bisexual, lesbian, and gay adults from religious upbringings. *Journal of Homosexuality*, *68*(14), 2323–2343. https://doi.org/10.1080/00918369.2020.1804255

Hershberger, S. L., & D'Augelli, A. R. (1995). The impact of victimization on the mental health and suicidality of lesbian, gay, and bisexual youths. *Developmental*

*Psychology*, *31*(1), 65. https://doi.org/10.1037/0012-1649.31.1.65

Hill, R. M., Rooney, E. E., Mooney, M. A., & Kaplow, J. B. (2017). Links between social support, thwarted belongingness, and suicide ideation among lesbian, gay, and bisexual college students. *Journal of Family Strengths*, *17*(2), 6. https://doi.org/10.58464/2168-670X.1350

Houston, E., & McKirnan, D. J. (2007). Intimate partner abuse among gay and bisexual men: Risk correlates and health outcomes. *Journal of Urban Health*, *84*(5), 681–690. https://doi.org/10.1007/s11524-007-9188-0

Huebner, D. M., Kegeles, S. M., Rebchook, G. M., Peterson, J. L., Neilands, T. B., Johnson, W. D., & Eke, A. N. (2014). Social oppression, psychological vulnerability, and unprotected intercourse among young Black men who have sex with men. *Health Psychology*, *33*(12), 1568–1578. https://doi.org/10.1037/hea0000031

Jeon, M. E., Robison, M., Robertson, L., Udupa, N. S., Potter, M. R., & Joiner, T. E. (2024). From identity-based distress to thinking "I am better off being dead:" Minority stress, posttraumatic cognitions, and suicidal ideation. *Journal of Affective Disorders*, *354*, 143–151. https://doi.org/10.1016/j.jad.2024.03.064

Johnson, J. G., Alloy, L. B., Panzarella, C., Metalsky, G. I., Rabkin, J. G., Williams, J. B. W., & Abramson, L. Y. (2001). Hopelessness as a mediator of the association between social support and depressive symptoms: Findings of a study of men with HIV. *Journal of Consulting and Clinical Psychology*, *69*(6), 1056–1060. https://doi.org/10.1037/0022-006X.69.6.1056

Kamen, C., Jabson, J. M., Mustian, K. M., & Boehmer, U. (2017). Minority stress, psychosocial resources, and psychological distress among sexual minority breast cancer survivors. *Health Psychology*, *36*(6), 529–537.

https://doi.org/10.1037/hea0000465

Katz-Wise, S. L., Sarda, V., Austin, S. B., & Harris, S. K. (2021). Longitudinal effects of gender minority stressors on substance use and related risk and protective factors among gender minority adolescents. *PLOS ONE*, *16*(6), e0250500. https://doi.org/10.1371/journal.pone.0250500

Kavanaugh, S. A., Taylor, A. B., Stuhlsatz, G. L., Neppl, T. K., & Lohman, B. J. (2020). Family and community support among sexual minorities of color: The role of sexual minority identity prominence and outness on psychological well-being. *Journal of GLBT Family Studies*, *16*(1), 1–17. https://doi.org/10.1080/1550428X.2019.1593279

Kecojevic, A., Basch, C. H., Kernan, W. D., Montalvo, Y., & Lankenau, S. E. (2019). Perceived social support, problematic drug use behaviors, and depression among prescription drugs-misusing young men who have sex with men. *Journal of Drug Issues*, *49*(2), 324–337. https://doi.org/10.1177/0022042619829246

Kim, S. Y., Velez, B., Daheim, J., & Lei, N. (2019). Validation of the work family conflict scale for sexual minority employees. *Journal of Career Assessment*, *27*(4), 594–609. https://doi.org/10.1177/1069072718788329

Kulick, A., Wernick, L. J., Woodford, M. R., & Renn, K. (2017). Heterosexism, depression, and campus engagement among LGBTQ college students: Intersectional differences and opportunities for healing. *Journal of Homosexuality*, *64*(8), 1125–1141. https://doi.org/10.1080/00918369.2016.1242333

Lampis, J., De Simone, S., & Belous, C. K. (2021). Relationship satisfaction, social support, and psychological well-being in a sample of Italian lesbian and gay individuals. *Journal of GLBT Family Studies*, *17*(1), 49–62.

https://doi.org/10.1080/1550428X.2020.1724844

Lee, E. A., Ashai, S., Teran, M., & Shin, R. Q. (2023). Intersectional microaggressions, mental health outcomes, and the role of social support among Black LGB adults. *Journal of Counseling Psychology, 70*(5), 464. https://doi.org/10.1037/cou0000684

Lefevor, G. T., & Skidmore, S. J. (2024). How can sexual and gender minority Latter-day Saints resolve ıdentity conflict and ımprove their mental health? results from a 2-to 4-year longitudinal study. *Behavior Therapy 56*(3), 580-593. https://doi.org/10.1016/j.beth.2024.08.006

Lehavot, K., & Simoni, J. M. (2011). The impact of minority stress on mental health and substance use among sexual minority women. *Journal of Consulting and Clinical Psychology*, *79*(2), 159–170. https://doi.org/10.1037/a0022839

Li, H., Liu, X., Zheng, Q., Zeng, S., & Luo, X. (2023). Minority stress, social support and mental health among lesbian, gay, and bisexual college students in China: a moderated mediation analysis. *BMC Psychiatry, 23*(1), 746. https://doi.org/10.1186/s12888-023-05202-z

Li, J., Mo, P. K. H., Wu, A. M. S., & Lau, J. T. F. (2017). Roles of self-stigma, social support, and positive and negative affects as determinants of depressive symptoms among HIV infected men who have sex with men in China. *AIDS and Behavior*, *21*(1), 261–273. https://doi.org/10.1007/s10461-016-1321-1

Liu, X., Li, H., Zeng, S., & Luo, X. (2024). Social support and cigarette smoking among homosexual college students in China: a moderated mediation model examining the roles of depression and disclosure. Current Psychology, 43(10), 8788-8798. https://doi.org/10.1007/s12144-023-04969-4

Liu, Y., Yang, Y., Fu, C., Lin, H., Wang, T., Wang, S., ... & Wang, J. (2023). Loneliness and depressive symptoms among men who have sex with men in China: a cross-sectional study. *Frontiers in Psychiatry, 14*, 1179703. https://doi.org/10.3389/fpsyt.2023.1179703

Liu, F., Wang, N., Chung, M. C., & Chui, H. (2024). Heterosexist events and psychological problems: internalized homophobia as a mediator and perceived parental support as a moderator. *Psychology & Sexuality, 15*(1), 82-96. https://doi.org/10.1080/19419899.2023.2209574.

Logie, C. H., Lacombe-Duncan, A., Poteat, T., & Wagner, A. C. (2017). Syndemic factors mediate the relationship between sexual stigma and depression among sexual minority women and gender minorities. *Women's Health Issues*, *27*(5), 592–599. https://doi.org/10.1016/j.whi.2017.05.003

Macbeth, A. J., Vidales, C. A., & Vogel, D. L. (2022). Perceived parental religiosity as a predictor of depression and substance use among LGBTQ+ individuals: The mediating role of perceived familial stigma. *Psychology of Religion and Spirituality*, *14*(1), 140–147. https://doi.org/10.1037/rel0000411

Mann, A. K., Fredrick, E. G., & Wilkins, B. T. (2024). Growing up in a rural area, social support, and internalized transphobia: Understanding Appalachian transgender mental and physical health. *Stigma and Health, 9*(4), 582–591. https://doi.org/10.1037/sah0000425

Marsland, S., Treyvaud, K., & Pepping, C. A. (2022). Prevalence and risk factors associated with perinatal depression in sexual minority women. *Clinical Psychology & Psychotherapy*, *29*(2), 611–621. https://doi.org/10.1002/cpp.2653

Mason, T. B., Lewis, R. J., & Heron, K. E. (2017). Indirect pathways connecting sexual

orientation and weight discrimination to disordered eating among young adult lesbians. *Psychology of Sexual Orientation and Gender Diversity*, *4*(2), 193–204. https://doi.org/10.1037/sgd0000220

Matijczak, A., McDonald, S. E., Tomlinson, C. A., Murphy, J. L., & O'Connor, K. (2020). The moderating effect of comfort from companion animals and social support on the relationship between microaggressions and mental health in LGBTQ+ emerging adults. *Behavioral Sciences*, *11*(1), 1. https://doi.org/10.3390/bs11010001

McCurdy, A. L., & Russell, S. T. (2023). Perceived parental social support and psychological control predict depressive symptoms for lesbian, gay, bisexual, transgender, queer, or questioning youth in the United States. *Child Development, 94*(3), 691-705. https://doi.org/10.1111/cdev.13894

McLaren, S., Schurmann, J., & Jenkins, M. (2015). The relationships between sense of belonging to a community GLB youth group; School, teacher, and peer connectedness; and depressive symptoms: Testing of a path model. *Journal of Homosexuality*, *62*(12), 1688–1702. https://doi.org/10.1080/00918369.2015.1078207

McLemore, K. A. (2018). A minority stress perspective on transgender individuals' experiences with misgendering. *Stigma and Health*, *3*(1), 53–64. https://doi.org/10.1037/sah0000070

Mehrtens, I. K. (2020). *The Relative Impact of Risk and Protective Factors on the Psychological Functioning of Sexual and Gender Minority Youth* (Doctoral dissertation, Louisiana State University). https://digitalcommons.lsu.edu/gradschool_dissertations/5324

Mereish, E. H., Cox, D. J., Harris, J. C., Anderson, Q. R., & Hawthorne, D. J. (2021).

Emerging ideas. Familial influences, shame, guilt, and depression among sexual minority adolescents. *Family Relations*, *70*(5), 1546–1555. https://doi.org/10.1111/fare.12514

Meza Lazaro, Y., & Bacio, G. A. (2023). Determinants of mental health outcomes among transgender Latinas: Minority stress and resilience processes. *Psychology of Sexual Orientation and Gender Diversity*, *10*(3), 451–460. https://doi.org/10.1037/sgd0000545

Milton, D. C., & Knutson, D. (2023). Family of origin, not chosen family, predicts psychological health in a LGBTQ+ sample. *Psychology of Sexual Orientation and Gender Diversity*, *10*(2), 269–278. https://doi.org/10.1037/sgd0000531

Mo, P. K. H., Chen, X., Lam, E. H. K., Li, J., Kahler, C. W., & Lau, J. T. F. (2020). The moderating role of social support on the relationship between anxiety, stigma, and intention to use illicit drugs among HIV-positive men who have sex with men. *AIDS and Behavior*, *24*(1), 55–64. https://doi.org/10.1007/s10461-019-02719-x

Moe, J., Sparkman-Key, N., Gantt-Howrey, A., Augustine, B., & Clark, M. (2023). Exploring the relationships between hope, minority stress, and suicidal behavior across diverse LGBTQ populations. *Journal of LGBTQ Issues in Counseling*, 17(1), 40–56. https://doi.org/10.1080/26924951.2022.2105773

Moody, C., & Smith, N. G. (2013). Suicide protective factors among trans adults. *Archives of Sexual Behavior*, *42*(5), 739–752. https://doi.org/10.1007/s10508-013-0099-8

Moran, T. E., Chen, C. Y., & Tryon, G. S. (2018). Bully victimization, depression, and the role of protective factors among college LGBTQ students. *Journal of Community Psychology*, *46*(7), 871–884. https://doi.org/10.1002/jcop.21978

Mustanski, B., & Liu, R. T. (2013). A longitudinal study of predictors of suicide attempts among lesbian, gay, bisexual, and transgender youth. *Archives of Sexual Behavior*, *42*(3), 437–448. https://doi.org/10.1007/s10508-012-0013-9

Mustanski, B., Newcomb, M. E., & Garofalo, R. (2011). Mental health of lesbian, gay, and bisexual youths: A developmental resiliency perspective. *Journal of Gay & Lesbian Social Services*, *23*(2), 204–225. https://doi.org/10.1080/10538720.2011.561474

Mutlu, Bağcı, Ece Sezen, & Cingoz-Ulu, Banu. (2023, December 9–10). *Self-silencing to heterosexism, minority stressors, and depression*. [Paper presentation]. International Gender Studies in Turkey Conference, Istanbul, Turkey.

Nelson, C. L. (2024). Exploring Resilience Among Midlife and Older Sexual Minority and Heterosexual Adults: A Multidimensional Analysis. *Journal of Applied Gerontology, 43*(11), 1716–1727. https://doi.org/10.1177/07334648241255496

Normansell-Mossa, K. M. (2023). *Exploration of the İmpact Of Gender-Affirming Care and Social Support on Executive Functioning and Mental Health in Gender-Diverse Autistic and Non-Autistic Adults*. [Doctoral dissertation, Brigham Young University]. https://scholarsarchive.byu.edu/etd/10445

Oetjen, H., & Rothblum, E. D. (2000). When lesbians aren't gay: Factors affecting depression among lesbians. *Journal of Homosexuality*, *39*(1), 49–73. https://doi.org/10.1300/J082v39n01_04

Ogunbajo, A., Iwuagwu, S., Williams, R., Biello, K. B., Kahler, C. W., Sandfort, T. G. M., & Mimiaga, M. J. (2020). Validation of depressive symptoms, social support, and minority stress scales among gay, bisexual, and other men who have with men (GBMSM) in Nigeria, Africa: A mixed methods approach. *BMC Public Health*,

*20*(1), 1023. https://doi.org/10.1186/s12889-020-09127-0

Otis, M. D., & Skinner, W. F. (1996). The prevalence of victimization and its effect on mental well-being among lesbian and gay people. *Journal of Homosexuality, 30*(3), 93–121. https://doi.org/10.1300/J082v30n03_05

Pachankis, J. E., Goldfried, M. R., & Ramrattan, M. E. (2008). Extension of the rejection sensitivity construct to the interpersonal functioning of gay men. *Journal of Consulting and Clinical Psychology*, *76*(2), 306–317. https://doi.org/10.1037/0022-006X.76.2.306

Pachankis, J. E., Hatzenbuehler, M. L., Klein, D. N., & Bränström, R. (2024). The role of shame in the sexual-orientation disparity in mental health: A prospective population-based study of multimodal emotional reactions to stigma. *Clinical Psychological Science, 12*(3), 486–504. 10.1177/21677026231177714

Pakenham, K. I., & Rinaldis, M. (2001). The role of illness, resources, appraisal, and coping strategies in adjustment to HIV/AIDS: The direct and buffering effects. *Journal of Behavioral Medicine*, *24*(3), 259–279. https://doi.org/10.1023/A:1010718823753

Palmer, C. W., & Francis, S. E. (2024). The role of family rejection of gender expression on minority stress and mental health of sexual and gender minority adolescents. *Sexuality Research and Social Policy, 21*(1), 998–1013. https://doi.org/10.1007/s13178-023-00881-5

Pate, A. R., DeShong, H. L., Stafford, T. W. D., & Nadorff, M. R. (2023). Impact of social support on suicidal ideation and attempts among gender minority adults. *The International Journal of Aging and Human Development, 96*(1), 117–130. https://doi.org/10.1177/00914150221128972

Patki, S. M., Gandhi, P., Walawalkar, A. P., & Goyal, A. A. (2024). Perceived social support, self-esteem, and depression among Indian trans men with and without sex reassignment surgery. *Palgrave Communications, 11*(1), 1-16. https://doi.org/10.1057/s41599-024-03270-4

Pereira, H. (2022). The impacts of sexual stigma on the mental health of older sexual minority men. *Aging & Mental Health*, *26*(6), 1281–1286. https://doi.org/10.1080/13607863.2021.1916883

Perry, N., Goldenberg, T., Huebner, D., Brown, A. L., Ware, D., Meanley, S., ... Plankey, M. (2022). Longitudinal associations of relationship support and strain and internalized homophobia with mental health among middle-aged and older gay and bisexual men. *Aging & Mental Health*, *27*(8), 1609–1618. https://doi.org/10.1080/13607863.2022.2146656

Pitoňák, M., Čihák, M., & Horne, S. G. (2024). LGBT+ people's vulnerability to suicide in Czechia: The role of minority stress-related factors in a high structural stigma context. *Psychology of Sexual Orientation and Gender Diversity*. Advance online publication. https://doi.org/10.1037/sgd0000785

Pollitt, A. M., Muraco, J. A., Grossman, A. H., & Russell, S. T. (2017). Disclosure stress, social support, and depressive symptoms among cisgender bisexual youth. *Journal of Marriage and Family*, *79*(5), 1278–1294. https://doi.org/10.1111/jomf.12418

Poteat, V. P., Mereish, E. H., DiGiovanni, C. D., & Koenig, B. W. (2011). The effects of general and homophobic victimization on adolescents' psychosocial and educational concerns: The importance of intersecting identities and parent support. *Journal of Counseling Psychology*, *58*(4), 597–609. https://doi.org/10.1037/a0025095

Puckett, J. A., Matsuno, E., Dyar, C., Mustanski, B., & Newcomb, M. E. (2019). Mental

health and resilience in transgender individuals: What type of support makes a difference? *Journal of Family Psychology*, *33*(8), 954–964. https://doi.org/10.1037/fam0000561

Ranjit, Y. S., Krishnan, A., Earnshaw, V. A., Weikum, D., Ferro, E. G., Sanchez, J., & Altice, F. L. (2023). Psychometric evaluation and validation of the HIV Stigma Scale in Spanish among men who have sex with men and transgender women. *Stigma and Health*, *8*(4), 437–444. https://doi.org/10.1037/sah0000302

Rimmer, S. E., Cohn, T. J., Hastings, S. L., Steele, J. C., & Woods, C. (2023). Does social support moderate the relationship between gender minority stress and suicide within a sample of transgender and gender diverse people? *Journal of Gay & Lesbian Mental Health*, *27*(3), 284–303. https://doi.org/10.1080/19359705.2021.1997855

Rivas-Koehl, M., Valido, A., Espelage, D. L., Robinson, L. E., Hong, J. S., Kuehl, T., Mintz, S., & Wyman, P. A. (2022). Understanding protective factors for suicidality and depression among U.S. sexual and gender minority adolescents: Implications for school psychologists. *School Psychology Review*, *51*(3), 290–303. https://doi.org/10.1080/2372966X.2021.1881411

Robles, G., Lee, Y. G., Hillesheim, J., Brusche, D., Lopez-Matos, J., Cain, D., & Starks, T. J. (2024). Peer social support moderates the impact of ethnoracial discrimination on mental health among young sexual minority men of color. *Journal of Social and Personal Relationships, 41*(9), 2455–2477. https://doi.org/10.1177/02654075241245735

Robles, G., Lee, J. J., Yu, M., & Starks, T. J. (2024). Multilevel analysis of sociopolitical contexts, social support, mental health, and alcohol use among partnered sexual

minority latino men in the U.S. *Journal of Racial and Ethnic Health Disparities, 11*, 1618–1627. https://doi.org/10.1007/s40615-023-01637-y

Rogowska, A. M., & Cisek, A. (2024). Minority stress, perceived social support, and depression in people diverse in sexual and gender minority status. *Psychology & Sexuality, 15*(4), 679–693. https://doi.org/10.1080/19419899.2024.2333858.

Rosario, M., Schrimshaw, E. W., & Hunter, J. (2005). Psychological distress following suicidality among gay, lesbian, and bisexual youths: Role of social relationships. *Journal of Youth and Adolescence*, *34*(2), 149–161. https://doi.org/10.1007/s10964-005-3213-y

Rotondi, N. K., Bauer, G. R., Travers, R., Travers, A., Scanlon, K., & Kaay, M. (2011). Depression in male-to-female transgender ontarians: Results from the Trans PULSE Project. *Canadian Journal of Community Mental Health*, *30*(2), 113–133. https://doi.org/10.7870/cjcmh-2011-0020

Salim, S. R., & Messman, T. L. (2024). Suicidal thoughts among young bisexual women: Sexual violence, minority stress, and interpersonal factors. *Journal of Counseling Psychology, 71*(5), 415–429. https://doi.org/10.1037/cou0000752

Samrock, S., Kline, K., & Randall, A. K. (2021). Buffering against depressive symptoms: Associations between self-compassion, perceived family support and age for transgender and nonbinary individuals. *International Journal of Environmental Research and Public Health*, *18*(15), 7938. https://doi.org/10.3390/ijerph18157938

Sarno, E. L., Dyar, C., Newcomb, M. E., & Whitton, S. W. (2022). Relationship quality and mental health among sexual and gender minorities. *Journal of Family Psychology*, *36*(5), 770–779. https://doi.org/10.1037/fam0000944

Sarno, E. L., Newcomb, M. E., & Mustanski, B. (2020). Rumination longitudinally

mediates the association of minority stress and depression in sexual and gender minority individuals. *Journal of Abnormal Psychology*, *129*(4), 355–363. https://doi.org/10.1037/abn0000508

Scandurra, C., Bochicchio, V., Dolce, P., Caravà, C., Vitelli, R., Testa, R. J., & Balsam, K. F. (2020). The Italian validation of the gender minority stress and resilience measure. *Psychology of Sexual Orientation and Gender Diversity*, *7*(2), 208–221. https://doi.org/10.1037/sgd0000366

Scheer, J. R., Clark, K. A., Talan, A., Cabral, C., Pachankis, J. E., & Rendina, H. J. (2021). Longitudinal associations between childhood sexual abuse-related PTSD symptoms and passive and active suicidal ideation among sexual minority men. *Child Abuse & Neglect*, *122*, 105353. https://doi.org/10.1016/j.chiabu.2021.105353

Seabra, D., Gato, J., Petrocchi, N., & do Céu Salvador, M. (2023). Shame experiences and psychopathology: The mediating role of self-compassion and social support in sexual minority individuals. *Journal of Evidence-Based Psychotherapies*, *22*(1), 137–152. https://doi.org/10.24193/jebp.2023.1.6

Sheets, R. L., & Mohr, J. J. (2009). Perceived social support from friends and family and psychosocial functioning in bisexual young adult college students. *Journal of Counseling Psychology*, *56*(1), 152–163. https://doi.org/10.1037/0022-0167.56.1.152

Shepherd, B. F., Chang, C. J., Dyar, C., Brochu, P. M., Selby, E. A., & Feinstein, B. A. (2024). Out of the closet, but not out of the woods: The longitudinal associations between identity disclosure, discrimination, and nonsuicidal self-injury among sexual minoritized young adults. *Psychology of Sexual Orientation and Gender Diversity, 11*(2), 294–304. https://doi.org/10.1037/sgd0000597

Shrader, C.-H., Salerno, J. P., Lee, J.-Y., Johnson, A. L., & Algarin, A. B. (2024). Mental health impact of multiple sexually minoritized and gender expansive stressors among LGBTQ+ young adults: a latent class analysis. Epidemiology and Psychiatric Sciences, 33, e22, 1–10https://doi.org/10.1017/S2045796024000118

Sigurvinsdottir, R., Gisladottir, B., Asgeirsdottir, B. B., & Sigfusdottir, I. D. (2024). Sexual attraction and non-suicidal self-harm: The role of stressors and psychological mediators. *Archives of Sexual Behavior, 53*, 1293–1306. https://doi.org/10.1007/s10508-023-02792-3

Skidmore, S. J., Lefevor, G. T., Huynh, K. D., & Berg, C. O. (2024). Development and initial validation of scales for coming out vigilance and positive coming out responses. *Sexuality & Culture, 28*(2), 771-793. https://doi.org/10.1007/s12119-023-10144-5

Smith, D. M., Wang, S. B., Carter, M. L., Fox, K. R., & Hooley, J. M. (2020). Longitudinal predictors of self-injurious thoughts and behaviors in sexual and gender minority adolescents. *Journal of Abnormal Psychology*, *129*(1), 114–121. https://doi.org/10.1037/abn0000483

Smith, E. R., & Perrin, P. B. (2017). Structural equation modeling linking perceived heterosexism, mental health, and nonsuicidal self-injury in ethnically diverse sexual minority men and women. *Traumatology*, *23*(3), 258–264. https://doi.org/10.1037/trm0000111

Soler, J. H., Caldwell, C. H., Córdova, D., Harper, G., & Bauermeister, J. A. (2018). Who counts as family? Family typologies, family support, and family undermining among young adult gay and bisexual men. *Sexuality Research and Social Policy*, *15*(2), 123–138. https://doi.org/10.1007/s13178-017-0288-7

Sullivan, K. S., Dodge, J., McNamara, K. A., Gribble, R., Keeling, M., Taylor-Beirne, S., Kale, C., Goldbach, J. T., Fear, N. T., & Castro, C. A. (2021). Perceptions of family acceptance into the military community among U.S. LGBT service members: A mixed-methods study. *Journal of Military, Veteran and Family Health*, *7*(s1), 90–101. https://doi.org/10.3138/jmvfh-2021-0019

Sutter, M., & Perrin, P. B. (2016). Discrimination, mental health, and suicidal ideation among LGBTQ people of color. *Journal of Counseling Psychology*, *63*(1), 98–105. https://doi.org/10.1037/cou0000126

Szymanski, D. M., Chung, Y. B., & Balsam, K. F. (2001). Psychosocial correlates of internalized homophobia in lesbians. *Measurement and Evaluation in Counseling and Development*, *34*(1), 27–38. https://doi.org/10.1080/07481756.2001.12069020

Szymanski, D. M., & Kashubeck-West, S. (2008). Mediators of the relationship between internalized oppressions and lesbian and bisexual women's psychological distress. *The Counseling Psychologist*, *36*(4), 575–594. https://doi.org/10.1177/0011000007309490

Tabaac, A., Perrin, P. B., & Benotsch, E. G. (2018). Discrimination, mental health, and body image among transgender and gender-non-binary individuals: Constructing a multiple mediational path model. *Journal of Gay & Lesbian Social Services*, *30*(1), 1–16. https://doi.org/10.1080/10538720.2017.1408514

Tabaac, A. R., Perrin, P. B., & Rabinovitch, A. E. (2016). The relationship between social support and suicide risk in a national sample of ethnically diverse sexual minority women. *Journal of Gay & Lesbian Mental Health*, *20*(2), 116–126. https://doi.org/10.1080/19359705.2015.1135842

Takeda, K., Rosenthal, L., & Arora, P. G. (2021). Internalizing symptoms, intersectional discrimination, and social support among Asian–Pacific Islander sexual and gender minority adults. *Cultural Diversity and Ethnic Minority Psychology*, *27*(3), 418–430. https://doi.org/10.1037/cdp0000442

Taliaferro, L. A., & Muehlenkamp, J. J. (2017). Nonsuicidal self-injury and suicidality among sexual minority youth: Risk factors and protective connectedness factors. *Academic Pediatrics*, *17*(7), 715–722. https://doi.org/10.1016/j.acap.2016.11.002

Tan, R. K. J., Yang, D. W. J., Le, D., Tan, A., Tyler, A., Tan, C., Kwok, C., Banerjee, S., & Wong, M. L. (2023). Minority statuses and mental health outcomes among young gay, bisexual and queer men in Singapore. *Journal of LGBT Youth*, *20*(1), 198–215. https://doi.org/10.1080/19361653.2021.1940414

Tantirattanakulchai, P., & Hounnaklang, N. (2022). Perceived social support and its relationship with depression among Bangkok's trans women. *Journal of Health Research*, *36*(2), 365–375. https://doi.org/10.1108/JHR-05-2020-0165

Tebbe, E. A., & Moradi, B. (2016). Suicide risk in trans populations: An application of minority stress theory. *Journal of Counseling Psychology*, *63*(5), 520–533. https://doi.org/10.1037/cou0000152

Testa, R. J., Habarth, J., Peta, J., Balsam, K., & Bockting, W. (2015). Development of the gender minority stress and resilience measure. *Psychology of Sexual Orientation and Gender Diversity*, *2*(1), 65–77. https://doi.org/10.1037/sgd0000081

Testa, R. J., Michaels, M. S., Bliss, W., Rogers, M. L., Balsam, K. F., & Joiner, T. (2017). Suicidal ideation in transgender people: Gender minority stress and interpersonal theory factors. *Journal of Abnormal Psychology*, *126*(1), 125–136. https://doi.org/10.1037/abn0000234

Thoma, B. C., Biernesser, C. L., & Win, E. (2025). Gender nonconformity, minority stress, and psychological distress among sexual minority adolescents. *LGBT health, 12*(4), 268-277. https://doi.org/10.1089/lgbt.2024.0059

Toomey, R. B., Trujillo, L., Abreu, R. L., Rios Garza, A., Hainsworth, S., & Zhao, Z. (2025). The potential harm of loss and grief narratives among families of transgender and nonbinary youth. *Journal of Counseling Psychology, 72*(3), 201–210. https://doi.org/10.1037/cou0000778

Tornello, S. L., & Patterson, C. J. (2016). Gay grandfathers: Intergenerational relationships and mental health. *Journal of Family Psychology*, *30*(5), 543–551. https://doi.org/10.1037/fam0000193

Travers, Á., Armour, C., Hansen, M., Cunningham, T., Lagdon, S., Hyland, P., Vallières, F., McCarthy, A., & Walshe, C. (2020). Lesbian, gay or bisexual identity as a risk factor for trauma and mental health problems in Northern Irish students and the protective role of social support. *European Journal of Psychotraumatology*, *11*(1), 1708144. https://doi.org/10.1080/20008198.2019.1708144

Trujillo, M. A., Perrin, P. B., Sutter, M., Tabaac, A., & Benotsch, E. G. (2017). The buffering role of social support on the associations among discrimination, mental health, and suicidality in a transgender sample. *International Journal of Transgenderism*, *18*(1), 39–52. https://doi.org/10.1080/15532739.2016.1247405

Tsai, C.-S., Huang, Y.-T., & Yen, C.-F. (2021). Experience of sexual orientation microaggression among young adult lesbian, gay, and bisexual individuals in Taiwan: Its related factors and association with mental health problems. *International Journal of Environmental Research and Public Health*, *18*(22), 11744. https://doi.org/10.3390/ijerph182211744

Tse, J., & Kwon, P. (2017). Extraversion as a moderator for resilience factors among gay men. *Journal of Gay & Lesbian Mental Health*, *21*(2), 115–131. https://doi.org/10.1080/19359705.2016.1267057

Tse, J., Kwon, P., & Faust, E. (2018). Extraversion as a moderator of resilience factors in lesbian, gay, and bisexual adults. *Journal of Gay & Lesbian Mental Health*, *22*(1), 36–51. https://doi.org/10.1080/19359705.2017.1374898

Turpin, R. E., Dyer, T. V., Dangerfield, D. T., Liu, H., & Mayer, K. H. (2020). Syndemic latent transition analysis in the HPTN 061 cohort: Prospective interactions between trauma, mental health, social support, and substance use. *Drug and Alcohol Dependence*, *214*, 108106. https://doi.org/10.1016/j.drugalcdep.2020.108106

Tüzün, Z., Başar, K., & Akgül, S. (2022). Social connectedness matters: Depression and anxiety in transgender youth during the COVID-19 pandemic. *The Journal of Sexual Medicine*, *19*(4), 650–660. https://doi.org/10.1016/j.jsxm.2022.01.522

Ueno, K., Gayman, M. D., Wright, E. R., & Quantz, S. D. (2009). Friends' sexual orientation, relational quality, and mental health among gay, lesbian, and bisexual youth. *Personal Relationships*, *16*(4), 659–670. https://doi.org/10.1111/j.1475-6811.2009.01245.x

Vale, M. T., & Bisconti, Toni. L. (2021). Age differences in sexual minority stress and the importance of friendship in later life. *Clinical Gerontologist*, *44*(3), 235–248. https://doi.org/10.1080/07317115.2020.1836107

Valente, P. K., Schrimshaw, E. W., Dolezal, C., LeBlanc, A. J., Singh, A. A., & Bockting, W. O. (2020). Stigmatization, resilience, and mental health among a diverse community sample of transgender and gender nonbinary individuals in the U.S. *Archives of Sexual Behavior*, *49*(7), 2649–2660. https://doi.org/10.1007/s10508-

020-01761-4

Van Dam, M. A. (2014). Lesbian disclosure, social support, and depression: A geopolitical perspective. *Sexuality Research and Social Policy*, *11*(3), 233–244. https://doi.org/10.1007/s13178-014-0160-y

Van Stolk-Cooke, K., Price, M., Dyar, C., Zimmerman, L., & Kaysen, D. (2024). Associations of past-year overall trauma, sexual assault and PTSD with social support for young adult sexual minority women. *European Journal of Psychotraumatology*, *15*(1), 2287911. https://doi.org/10.1080/20008066.2023.2287911

Veale, J. F., Peter, T., Travers, R., & Saewyc, E. M. (2017). Enacted stigma, mental health, and protective factors among transgender youth in Canada. *Transgender Health*, *2*(1), 207–216. https://doi.org/10.1089/trgh.2017.0031

Velasco, J., Miranda-Tena, A., & Sanmartín, F. J. (2024). Bisexual discrimination, ınternalized binegativity and their ımpact on mental health. *Sexuality Research and Social Policy, 21*(3), 1100-1111. https://doi.org/10.1007/s13178-024-00990-9

Velez, B. L., Kim, S. Y., Cox, R., Manosalvas, K., & Daheim, J. (2023). A test of the integrative mediation model of minority stress with sexual minority adults. *Psychology of Sexual Orientation and Gender Diversity*, *10*(1), 56–69. https://doi.org/10.1037/sgd0000498

Vincke, J., & Van Heeringen, K. (2004). Summer holiday camps for gay and lesbian young adults: An evaluation of their impact on social support and mental well-being. *Journal of Homosexuality*, *47*(2), 33–46. https://doi.org/10.1300/J082v47n02_02

Vogel, E. A., Flentje, A., Lunn, M. R., Obedin-Maliver, J., Capriotti, M. R., Ramo, D. E.,

& Prochaska, J. J. (2024). Active social media use and health indicators among sexual and gender minority adults. *LGBT health, 11*(4), 292-300. https://doi.org/10.1089/lgbt.2023.0170

Vogel, E. A., Ramo, D. E., Prochaska, J. J., Meacham, M. C., Layton, J. F., & Humfleet, G. L. (2021). Problematic social media use in sexual and gender minority young adults: Observational study. *JMIR Mental Health*, *8*(5), e23688. https://doi.org/10.2196/23688

Wang, Y., Miao, N., & Chang, S. (2021). Internalized homophobia, self-esteem, social support and depressive symptoms among sexual and gender minority women in Taiwan: An online survey. *Journal of Psychiatric and Mental Health Nursing*, *28*(4), 601–610. https://doi.org/10.1111/jpm.12705

Watts, K. J., Wagaman, M. A., Eaton, A. D., Leung, V. W., & Craig, S. L. (2025). Examining the role of peer and family belongingness in the mental health of Black LGBTQ+ Youth. *Child & Youth Services, 46*(1), 153-172. https://doi.org/10.1080/0145935X.2023.2261364

Wei, L., Yu, B., Zhai, M., Li, J., Liu, D., Liu, X., ... & Yan, H. (2024). Adverse childhood experiences, sexual minority stigma, social support and depressive symptoms among Chinese men who have sex with men: A moderated mediation modelling analysis. *Current Psychology, 43*(2), 1169-1178. https://doi.org/10.1007/s12144-023-04324-7

Woodford, M. R., Kulick, A., & Atteberry, B. (2015). Protective factors, campus climate, and health outcomes among sexual minority college students. *Journal of Diversity in Higher Education*, *8*(2), 73–87. https://doi.org/10.1037/a0038552

Woodward, E. N., & Pantalone, D. W. (2012). The role of social support and negative

affect in medication adherence for HIV-infected men who have sex with men. *Journal of the Association of Nurses in AIDS Care*, *23*(5), 388–396. https://doi.org/10.1016/j.jana.2011.09.004

Wright, M. F., Wachs, S., & Gámez-Guadix, M. (2022). The role of perceived gay-straight alliance social support in the longitudinal association between homophobic cyberbullying and LGBTQIA adolescents' depressive and anxiety symptoms. *Journal of Youth and Adolescence*, *51*(7), 1388–1396. https://doi.org/10.1007/s10964-022-01585-6

Yang, X., Wang, L., Gu, Y., Song, W., Hao, C., Zhou, J., Zhang, Q., & Zhao, Q. (2016). A cross-sectional study of associations between casual partner, friend discrimination, social support and anxiety symptoms among Chinese transgender women. *Journal of Affective Disorders*, *203*, 22–29. https://doi.org/10.1016/j.jad.2016.05.051

Zea, M. C., Reisen, C. A., & Poppen, P. J. (1999). Psychological well-being among Latino lesbians and gay men. *Cultural Diversity and Ethnic Minority Psychology*, *5*(4), 371–379. https://doi.org/10.1037/1099-9809.5.4.371

Zhang, J., Lo, H. H., & Au, A. M. (2021). The buffer of resilience in the relations of gender-related discrimination, rejection, and victimization with depression among Chinese transgender and gender non-conforming individuals. *Journal of Affective Disorders*, *283*, 335–343. https://doi.org/10.1016/j.jad.2021.01.059

Zhou, T., Chen, Q., & Zhong, X. (2024). Associations between sexual identity stigma and positive sexual identity, and depression among men who have sex with men in China: A mediation analysis using structural equation modelling. *Annals of Epidemiology, 96*, 32-39. https://doi.org/10.1016/j.annepidem.2024.05.010

**Supplemental Material 3. Effect sizes by sub-categories of social support**

| Category | # studies | *k* effects | *N* | *r* | 95% CI |
|---|---|---|---|---|---|
| Total | 253 | 689 | 111188 | -.26 | [-.27; -.24] |
| Family (supercategory) | 104 | 183 | 39122 | -.26 | [-.28; -.23] |
| Parents | 21 | 41 | 10727 | -.21 | [-.26; -.16] |
| Siblings | 1 | 2 | 153 | -.22 | [-.42; -.02] |
| Peers (supercategory) | 73 | 142 | 26501 | -.22 | [-.28; -.17] |
| Peers (cishetero) | 14 | 21 | 4396 | -.16 | [-.37; .05] |
| Peers (SGM) | 8 | 16 | 3433 | -.19 | [-.3; -.08] |
| Partner (supercategory) | 35 | 63 | 12747 | -.23 | [-.3; -.17] |
| School/Work (supercategory) | 22 | 47 | 11037 | -.21 | [-.29; -.14] |
| School | 12 | 23 | 7706 | -.22 | [-.27; -.16] |
| Work | 11 | 24 | 3831 | -.23 | [-.36; -.10] |

*Note*. All effects were significant

**Supplemental Material 4. Associations between structural stigma and size of the association between social support and internalizing psychopathology**

S4a. Moderator effect in European geolocated studies

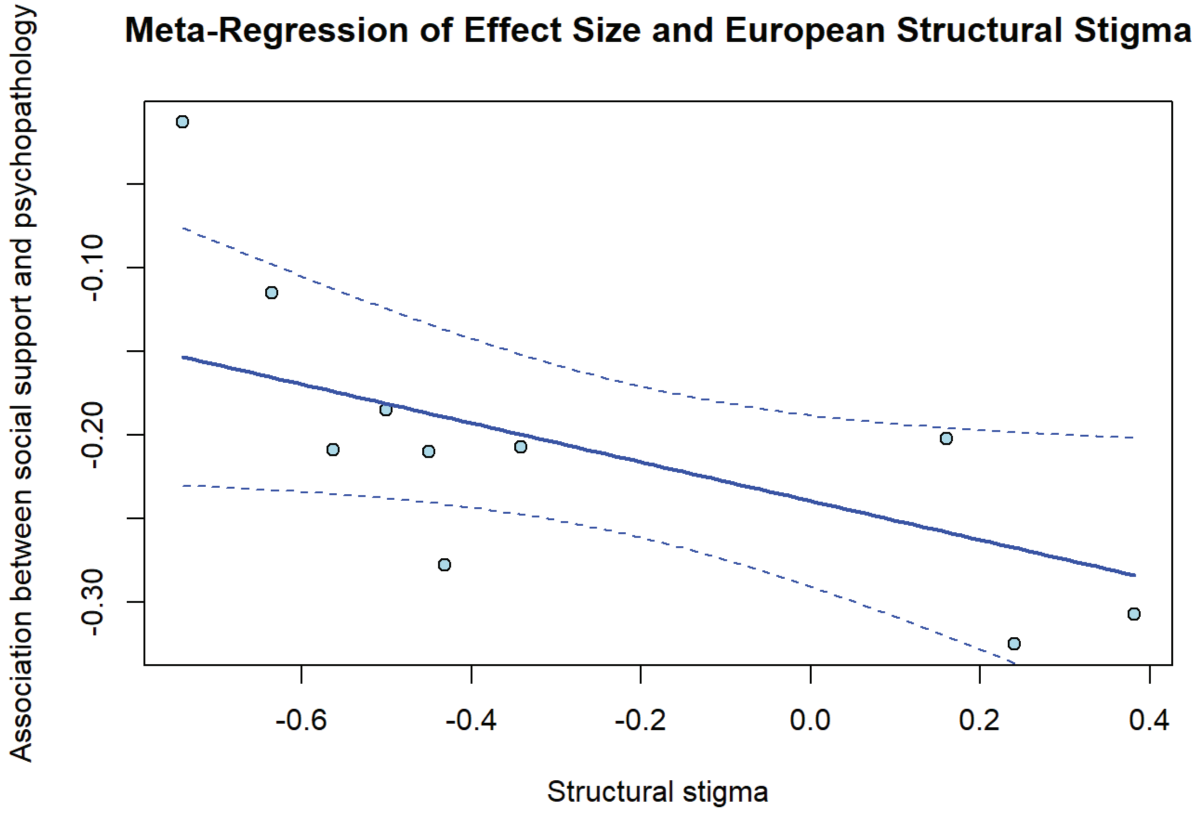


$B$ = -.12, $p$ = .051

S4b. Moderator effect in American geolocated studies

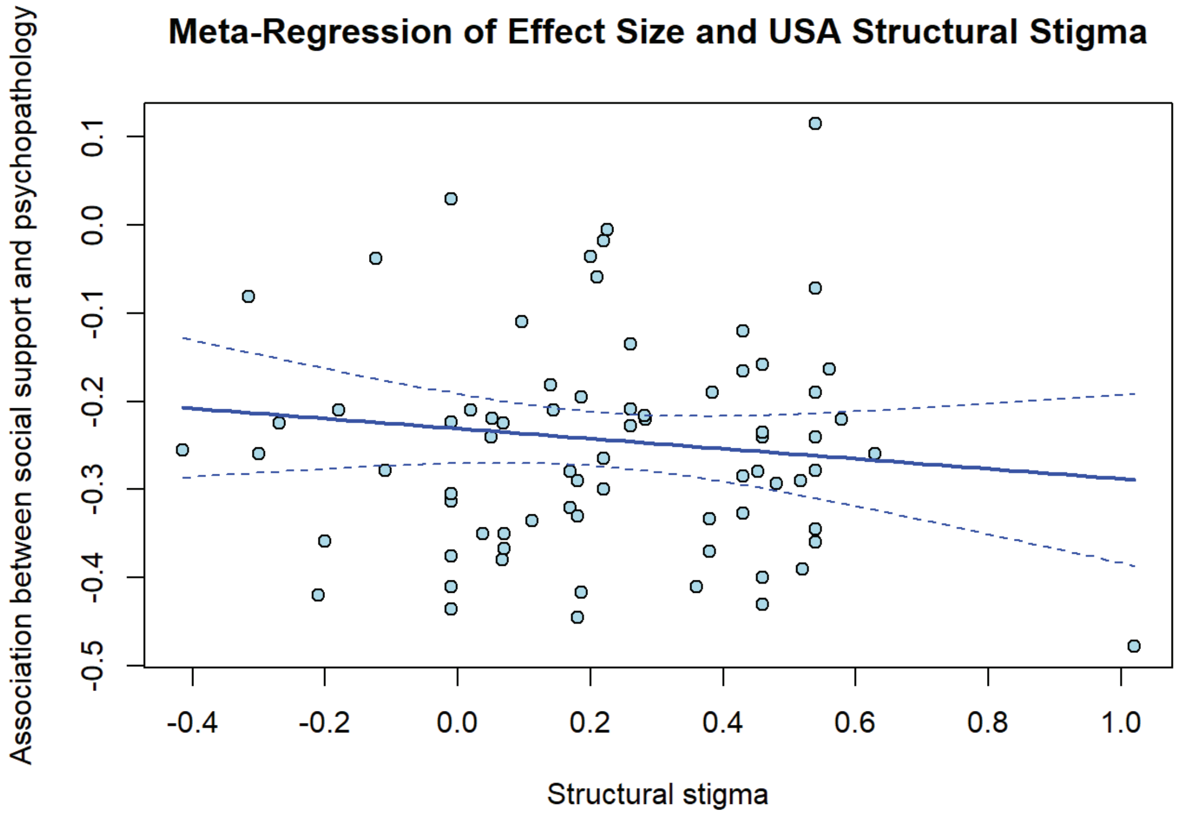


$B$ = -.06, $p$ = .332
